# Synthesis, biological evaluation and computational studies of pyrazole derivatives as *Mycobacterium tuberculosis* CYP121A1 inhibitors†

**DOI:** 10.1039/d2md00155a

**Published:** 2022-08-16

**Authors:** Lama A. Alshabani, Amit Kumar, Sam J. Willcocks, Gayathri Srithiran, Sanjib Bhakta, D. Fernando Estrada, Claire Simons

**Affiliations:** School of Pharmacy & Pharmaceutical Sciences, Cardiff University King Edward VII Avenue Cardiff CF10 3NB UK simonsc@cardiff.ac.uk; Department of Biochemistry, Jacobs School of Medicine and Biomedical Science, University at Buffalo Buffalo New York-14203 USA; Department of Infection Biology, The London School of Hygiene and Tropical Medicine London WC1E 7HT UK; Mycobacteria Research Laboratory, Institute of Structural and Molecular Biology, Department of Biological Sciences, Birkbeck, University of London London WC1E 7HX UK

## Abstract

A series of imidazole and triazole diarylpyrazole derivatives were prepared using an efficient 5-step synthetic scheme and evaluated for binding affinity with *Mycobacterium tuberculosis* (Mtb) CYP121A1 and antimycobacterial activity against Mtb H37Rv. Antimycobacterial susceptibility was measured using the spot-culture growth inhibition assay (SPOTi): the imidazoles displayed minimum inhibitory concentration (MIC_90_) in the range of 3.95–12.03 μg mL^−1^ (10.07–33.19 μM) with 11f the most active, while the triazoles displayed MIC_90_ in the range of 4.35–25.63 μg mL^−1^ (11.88–70.53 μM) with 12b the most active. Assessment of binding affinity using UV-vis spectroscopy showed that for the imidazole series, the propyloxy (11f) and isopropyloxy (11h) derivatives of the 4-chloroaryl pyrazoles displayed Mtb CYP121A1 type II binding affinity with *K*_d_ 11.73 and 17.72 μM respectively compared with the natural substrate cYY (*K*_d_ 12.28 μM), while in the triazole series, only the methoxy substitution with the 4-chloroaryl pyrazole (12b) showed good type II Mtb CYP121A1 binding affinity (*K*_d_ 5.13 μM). Protein-detected 1D ^19^F-NMR spectroscopy as an orthogonal strategy was used to evaluate ligand binding independent of perturbations at the haem. For imidazole and triazole compounds, perturbations were more intense than cYY indicating tighter binding and confirming that ligand coordination occurs in the substrate-binding pocket despite very modest changes in UV-vis absorbance, consistent with computational studies and the demonstrated potential anti-tuberculosis properties of these compounds.

## Abstract 2

Pyrazole derivatives were evaluated using UV-vis and protein-detected 1D ^19^F-NMR (Mtb CYP121A1 binding affinity), antimycobacterial determination (MIC) and computational studies illustrating the potential anti-TB properties of the compounds.

## Graphical abstract

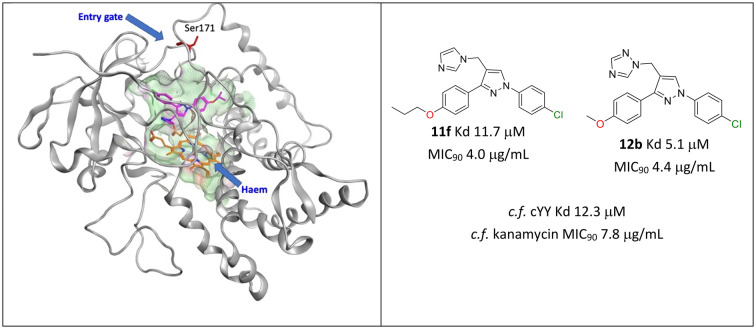

## Introduction

Tuberculosis (TB) was, prior to COVID-19, the leading cause of death from a single infectious agent. The impact of COVID-19 on TB has been substantial, most notably with TB resources, funding and facilities being redirected for COVID-19 and also a reduction in reporting data for newly diagnosed TB patients.^1^ Reduced access to testing facilities has resulted in an increase in TB related deaths in 2020 with estimates of 1.3 million deaths in HIV-negative people and 214 000 deaths in HIV-positive people.^1^ The rise in drug-resistant strains of *Mycobacterium tuberculosis* (Mtb), the main causative agent of TB, continues to be of concern with rifampicin resistant TB (RR-TB), multidrug resistant TB (MDR-TB) which is resistant to both first line anti-TB drugs rifampicin and isoniazid, and extremely drug resistant TB (XDR-TB) which is resistant against rifampicin and any second line fluoroquinolone plus one second line injectable anti-TB drug, exacerbating the challenge of effectively treating TB.^2^ Therefore, research looking at alternative Mtb targets, for which no resistance is already present in the population, is of interest to provide additional therapeutic drugs as part of the combinatorial drug approach used for TB treatment.

**Fig. 1:**
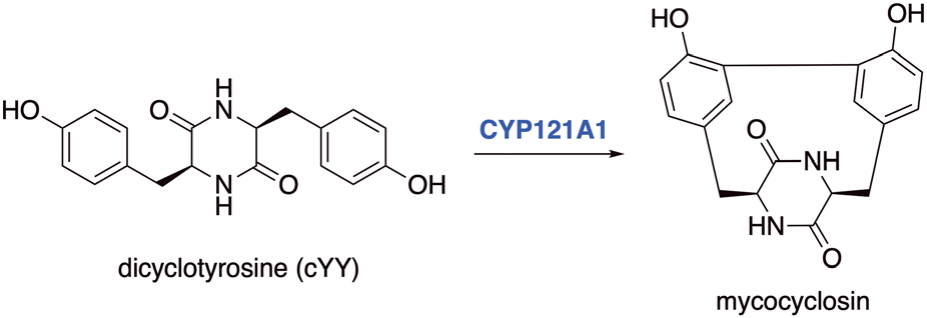
Mycocyclosin formed through a CYP121A1 mediated C–C bond formation between carbon atoms in the *ortho*-positions of two tyrosine groups in the cyclodipeptide dicyclotyrosine (cYY).

A target of interest is cytochrome P450 121A1 (CYP121A1, mycocyclosin synthase), an essential enzyme for Mtb viability,^3^ which catalyses the biotransformation of the tyrosine dipeptide cyclo-l-Tyr-l-Tyr (cYY) to mycocyclosin (Fig. 1).^4^ The exact role of mycocyclosin is not known, however it has been suggested that it may have an essential role in cell growth or structural stability, alternatively inhibition of CYP121A1 could result in an accumulation of cYY, which may be toxic to Mtb.^4,5^

**Fig. 2:**
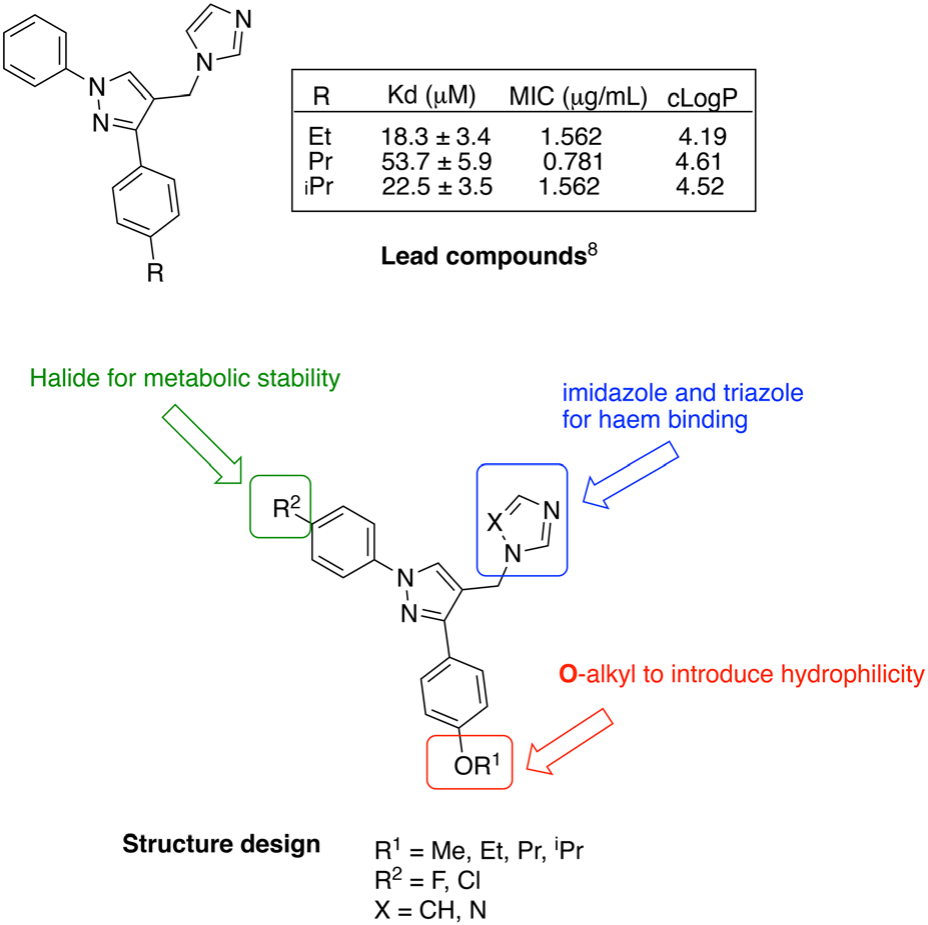
Lead diarylpyrazole derivatives^9^ and structure design of new compounds to improve drug likeness.

CYP121A1 inhibitors with a range of structural scaffolds have been described by us^6–9^ and others.^10–12^ In all cases a basic nitrogen containing group with the potential to bind to the iron of the haem group is included, although indirect water-mediated binding with the haem is occasionally observed and/or binding of the nitrogen containing group with Gln395/Arg386, which would block access of cYY to the active site.^6–12^ One of the more promising scaffolds is the diarylpyrazole series^6,9^ with optimal binding observed for imidazole as the nitrogen containing binding group and substitution of one of the aryl rings with lipophilic alkyl groups (Fig. 2).

Presented here is a further design of these lead pyrazole derivatives to consider drug likeness, incorporating *O*-alkyl substitutions in one aryl ring to improve hydrophilic properties compared with the lead compounds, and the addition of a halide (F or Cl) in the second aryl ring for metabolic stability (Fig. 2). Imidazole and 1,2,4-triazole azole moieties are included as the basic nitrogen binding groups to evaluate effect on binding affinity and antimycobacterial activity.

## Results and discussion

### Chemistry

**Scheme 1:**
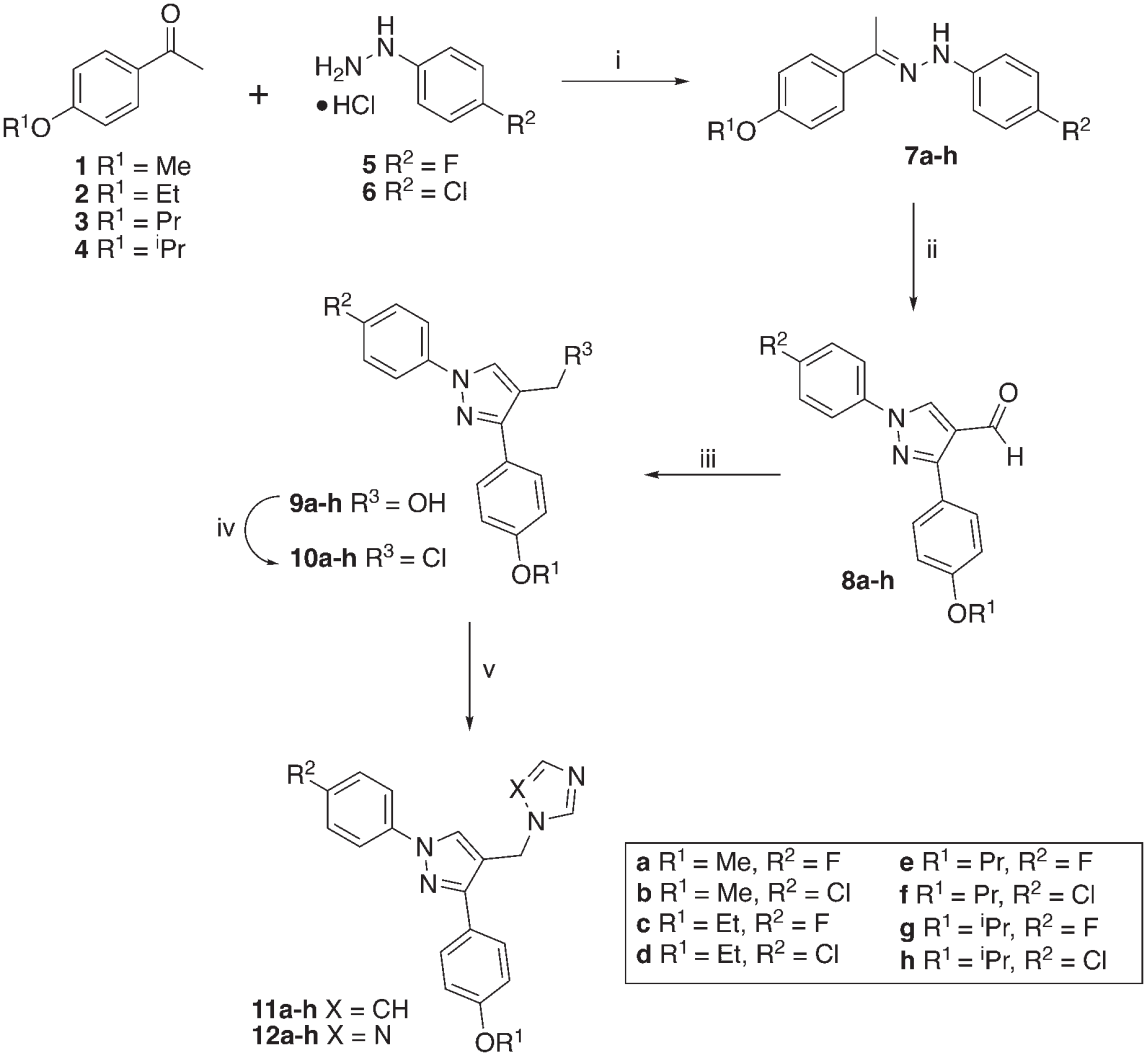
Reagents and conditions: (i) Et_3_N, AcOH, EtOH, 83 °C, 1 h, 79–100%; (ii) POCl_3_, DMF, 60 °C, 3 h, 65–100%; (iii) NaBH_4_, EtOH, r.t, 1 h, 68–98%; (iv) SOCl_2_, CH_2_Cl_2_, 40 °C, 4 h; (v) imidazole or triazole, K_2_CO_3_, CH_3_CN, 45 °C, 1 h then 70 °C, overnight, yields see Table 1.

The hydrazines (7) were prepared following previously described methodology^6,9^ by reaction of the 4-alkoxy-acetophenones (1–4) with either 4-fluoro or 4-chlorophenylhydrazine hydrochloride (5 or 6) in EtOH with acetic acid as the acid catalyst (Scheme 1). The hydrazines (7) were converted to the aldehydes (8) on reaction with Vilsmeier–Haack reagent, generated *in situ* from DMF and POCl_3_, and obtained as solids in good yields (65–100%).

The aldehydes (8) were reduced with NaBH_4_ and the alcohols (9), obtained after extraction with EtOAc in yields of 68–98%, were converted to the chlorides (10) on treatment with SOCl_2_ in CH_2_Cl_2_ at 40 °C for 4 h. The crude chlorides (10) were obtained in very high yields (93–100%) except for the isopropyl/fluoride derivative (48%), and were used immediately in the next reaction, which involved the generation of the imidazole or triazole anion by reaction of imidazole or triazole with K_2_CO_3_ in CH_3_CN at 45 °C for 1 h, followed by the addition of the chlorides and heating at 70 °C overnight (Scheme 1). The final imidazole (11) and triazole (12) products were obtained after purification by gradient column chromatography with generally good yields (Table 1).

**Table 1:** Yields and mp data for imidazoles (11) and triazoles (12)

Compd	R^1^	R^2^	X	Yield (%)	m.p. (°C)
11a	Me	F	CH	70	150–152
11b	Me	Cl	CH	61	136–138
11c	Et	F	CH	45	118–120
11d	Et	Cl	CH	22	124–126
11e	Pr	F	CH	66	120–122
11f	Pr	Cl	CH	63	90–92
11g	^i^Pr	F	CH	58	96–98
11h	^i^Pr	Cl	CH	94	98–100
12a	Me	F	N	58	150–152
12b	Me	Cl	N	60	124–126
12c	Et	F	N	52	140–142
12d	Et	Cl	N	60	122–124
12e	Pr	F	N	58	120–122
12f	Pr	Cl	N	72	114–116
12g	^i^Pr	F	N	53	118–120
12h	^i^Pr	Cl	N	81	98–100

### Antimycobacterial activity (MIC_90_)

All compounds were evaluated for MIC_90_ against Mtb H37Rv using the SPOTi assay as previously described.^13^ The imidazoles all showed good antimycobacterial activity (Table 2) with optimal activity observed for the propyloxy/Cl (11f, MIC_90_ 4.0 μg mL^−1^, 10.1 μM) and isopropyloxy/F (11h, MIC_90_ 4.4 μg mL^−1^, 11.9 μM) derivatives. The activity observed for all the imidazole derivatives (except 11c) and the triazoles 12b, 12d and 12f was comparable with the standard kanamycin (MIC_90_ 7.8 μg mL^−1^, 16.1 μM), which is an important second line drug for the treatment of MDR-TB.^14^

**Table 2:** MIC_90_ of compounds against Mtb H37Rv

Compd	MIC_90_^a^ (μg mL^−1^)	MIC_90_^a^ (μM)	cLog *P*^b^
11a	4.3	12.3	3.32
11b	6.0	16.5	3.72
11c	12.0	33.2	3.66
11d	5.0	13.3	4.06
11e	6.0	15.9	4.15
11f	4.0	10.1	4.55
11g	4.3	11.4	3.98
11h	6.0	15.3	4.38
12a	12.8	36.7	2.83
12b	4.4	11.9	3.23
12c	25.6	70.5	3.17
12d	6.4	16.8	3.57
12e	20.9	55.5	3.65
12f	6.4	16.2	4.05
12g	20.9	55.5	3.49
12h	25.6	65.1	3.89
*Isoniazid*	*0.2*	*1.8*	*−0.64*
*Kanamycin*	*7.8*	*16.1*	*−7.58*
*Rifampicin*	*0.2*	*0.3*	*—*

a Results are the average of two independent experiments.

b Crippen's fragmentation.^15^

### Mtb CYP121A1 binding affinity

All sixteen final compounds (11a–h and 12a–h) were subject to a preliminary binding affinity screen, which identified seven imidazole (11a, 11c–h) and one triazole derivatives (12b) for further evaluation. Evaluation of inhibitor affinity was carried out in a 96-well plate format. The *K*_d_ of cYY in this assay (12 μM) was consistent with its previously reported value.^4,16^ As a comparison, all inhibitors were titrated manually in a 1 cm quartz cuvette for a single trial. The calculated *K*_d_ values closely matched the results from the plate-reader assay.

The methoxy triazole derivative with a chloroaryl group (12b) displayed the tightest binding affinity with *K*_d_ 5.1 ± 1.5 μM, and in the imidazole series the derivatives with a chloroaryl group were generally more effective in binding to Mtb CYP121A1 compared with the compounds with a fluoroaryl group. The propyloxy derivative (11f, *K*_d_ 11.7 ± 5.4 μM) displayed binding affinity comparable with cYY (*K*_d_ 12.3 ± 1.1 μM) (Table 3).

**Table 3:** Mtb CYP121A1 binding affinity and Soret band shift

Compd	CYP121A1 *K*_d_ (μM)	Soret shift (nm)
11a	65.0 ± 16.6	417 to 421
11c	54.0 ± 5.2	417 to 422
11d	18.8 ± 10.2	417 to 420
11e	22.3 ± 4.2	417 to 420
11f	11.7 ± 5.4	417 to 419
11g	38.5 ± 8.7	417 to 419
11h	17.7 ± 6.9	417 to 419
12b	5.1 ± 1.5	417 to 417
cYY	12.3 ± 1.1	417 to 395

As previously stated cYY in the co-crystal structure displays indirect binding with the haem *via* interstitial water molecules.^4,16^

**Fig. 3:**
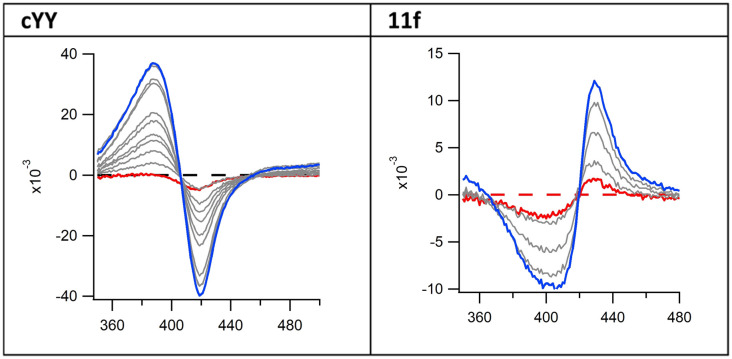
Binding difference spectra of cYY (type I) and pyrazole exemplar 11f (type II).

However, absorbance changes from cYY titration in solution produce a typical type-I spectral response, indicating displacement of the haem water molecule(s) and induction of a high-spin form of the iron. The pyrazoles described here displayed a type II binding profile suggesting direct binding with the haem (Fig. 3 and S1 and S2†).

**Fig. 4:**
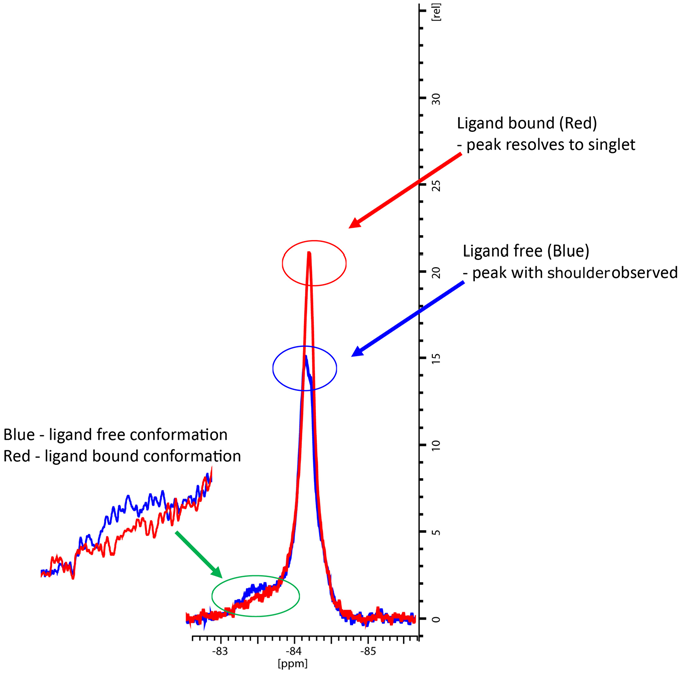
^19^F NMR spectra of BTFA-labelled CYP121_S171C (blue) alone and with cYY bound (red).

In the co-crystal structure with cYY, CYP121A1 coordinates the substrate within the active site while also preserving the space and hydrogen-bonding necessary to maintain a two-molecule water bridge to the haem.^4^ CYP121A1 is also known to coordinate the inhibitor fluconazole concomitantly with the haem water ligand.^17^ In practice, such coordination is difficult to detect by absorbance difference spectra alone, which rely on ligand-induced displacement of the water molecule followed by direct azole ligation to the haem. In light of this possibility, protein-detected 1D ^19^F-NMR spectroscopy was utilised as an orthogonal strategy to evaluate ligand binding independent of perturbations at the haem. As described recently,^18^ BTFA labelling of an S171C mutation located within the CYP121A1 FG-loop serves as a direct reporter of protein–ligand interactions. The FG-loop in CYPs is known to undergo extensive structural remodelling in response to ligand binding.^19–21^ In ligand-free CYP121A1, the conformational heterogeneity of the loop is indicated by at least three separate resonances (Fig. 4). As reported previously,^18^ a 3-fold addition of cYY reduces the intensity of the broad down-field resonance at −83.5 ppm while increasing the intensity of the major upfield resonance near −84.5 ppm, thus indicating reduced heterogeneity of the FG-loop conformation and an increase in the ligand-bound state (Fig. 4).

To evaluate the interaction with the compounds, ^19^F-NMR spectra were acquired for BTFA-labelled CYP121_S171C in the presence of 3-fold excess concentrations of representative imidazole and triazole derivatives. For comparison, spectra were overlayed with ligand-free and cYY-bound spectra recorded from samples from the same batch of labelled protein and at matching protein concentrations.

**Fig. 5:**
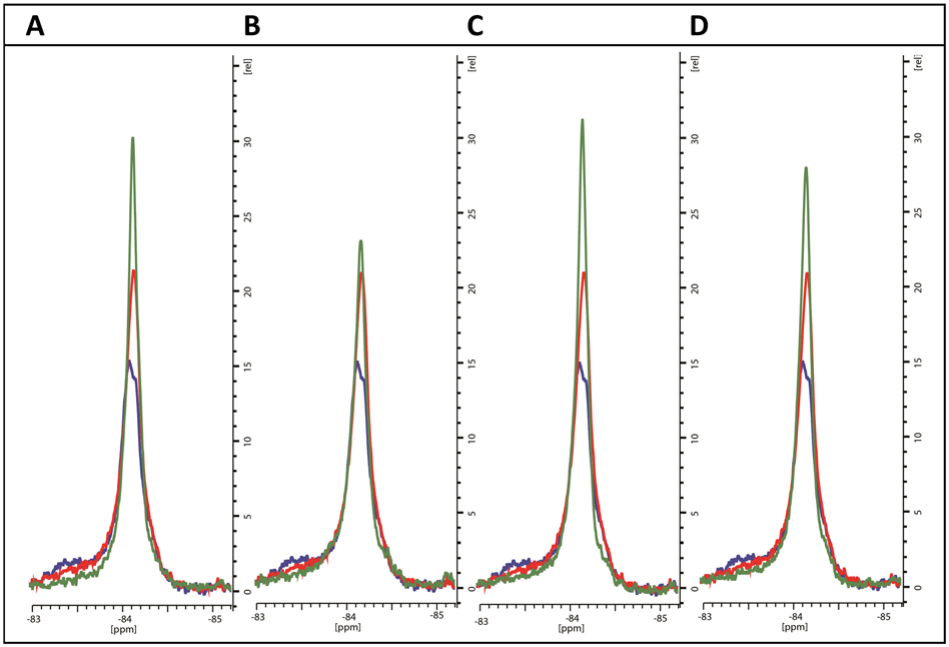
^19^F NMR spectra of BTFA-labelled CYP121_S171C (blue) alone, with cYY bound (red) and with exemplar pyrazole ligands (green) (A) 11a, (B) 11g, (C) 12b and (D) 12d.

As expected, the perturbations induced by the imidazole derivatives 11a and 11g are consistent with an interaction that occurs within the CYP121 active site, since both compounds elicit similar changes in the NMR spectrum of the FG-loop when compared to cYY (Fig. 5A and B). Moreover, the relative extent of these perturbations aligns with the change in the Soret peak of absorbance assays (Table 3). Interestingly, NMR spectra recorded in the presence of the triazole derivatives 12b and 12d were generally not in agreement with the absorbance data. These compounds elicited a weak spectral response in the UV-vis assay when compared with the imidazole derivatives. However, their presence induced all the characteristic changes of ligand-binding in ^19^F-NMR spectra. For both compounds, perturbations were more intense than that of cYY (Fig. 5C and D). Our interpretation of this apparent disagreement in binding behaviour is that weaker triazole-coordination of the haem may also allow for preservation of the haem-coordinated water ligand, as indicated by weaker absorbance data overall, and similar to the non-haem binding mode observed with fluconazole. Hence, the detection of ligand-binding by ^19^F-NMR, and thus confirmation that ligand coordination occurs in the substrate-binding pocket despite very modest changes in absorbance, is consistent with the demonstrated growth inhibition potential of these compounds.

### Computational studies

Mtb CYP121A1 protein-ligand complexes were generated through docking of compounds 11 and 12 with the crystal structure of Mtb CYP121A1 co-crystallised with cYY (pdb 3G5H^4^) using Molecular Operating Environment (MOE) software.^22^ The protein–ligand complexes were then subject to 150 ns molecular dynamics (MD) simulations using the Desmond programme of Schrödinger software.^23^

**Fig. 6:**
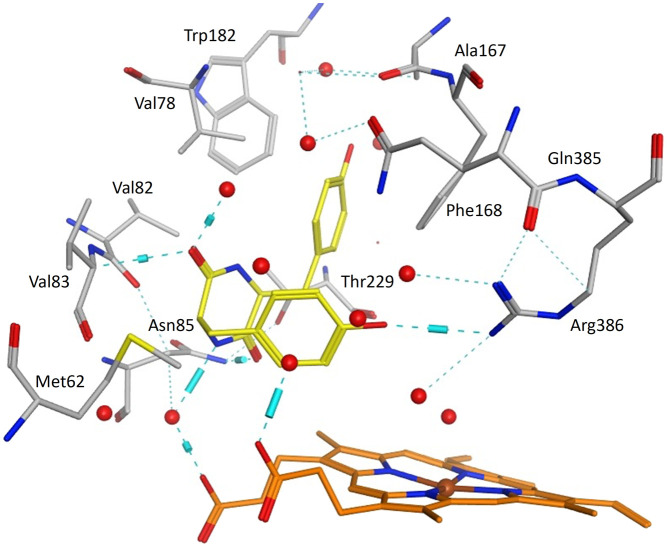
cYY (yellow) binding in Mtb CYP121A1 active site (pdb 3G5H). Haem shown in orange, water molecules as red spheres, H-bonding as blue lines/cylinders.

cYY sits to one side of the haem and is tethered through a H-bonding interaction between one phenolic OH and Arg386 on one side of the active site and through H-bonding interactions between the diketopiperazine ring C=O and NH groups with Val83 and Asn85 on the opposite side of the active site (Fig. 6).

**Fig. 7:**
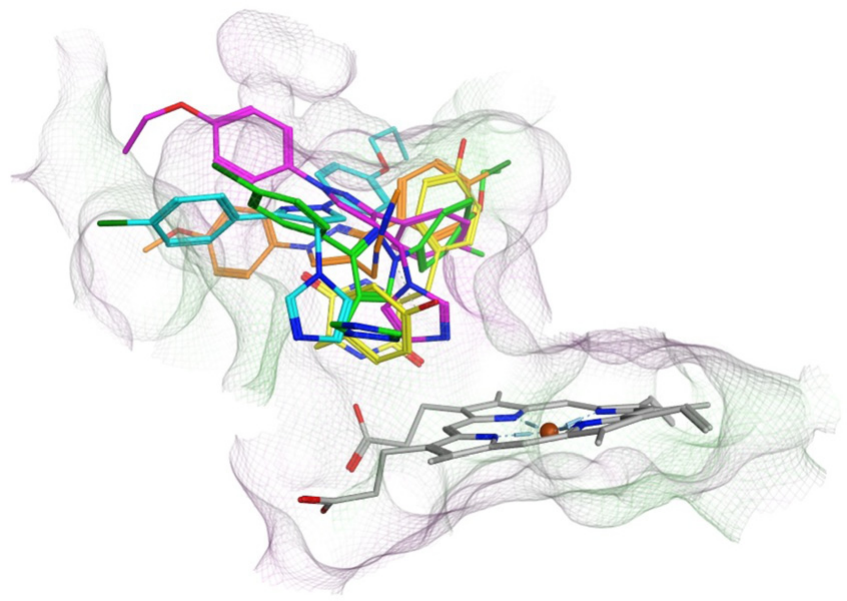
Comparison of binding position of pyrazoles 11c (magenta), 11f (cyan), 12b (orange) and 12d (green) with cYY (yellow).

**Fig. 8:**
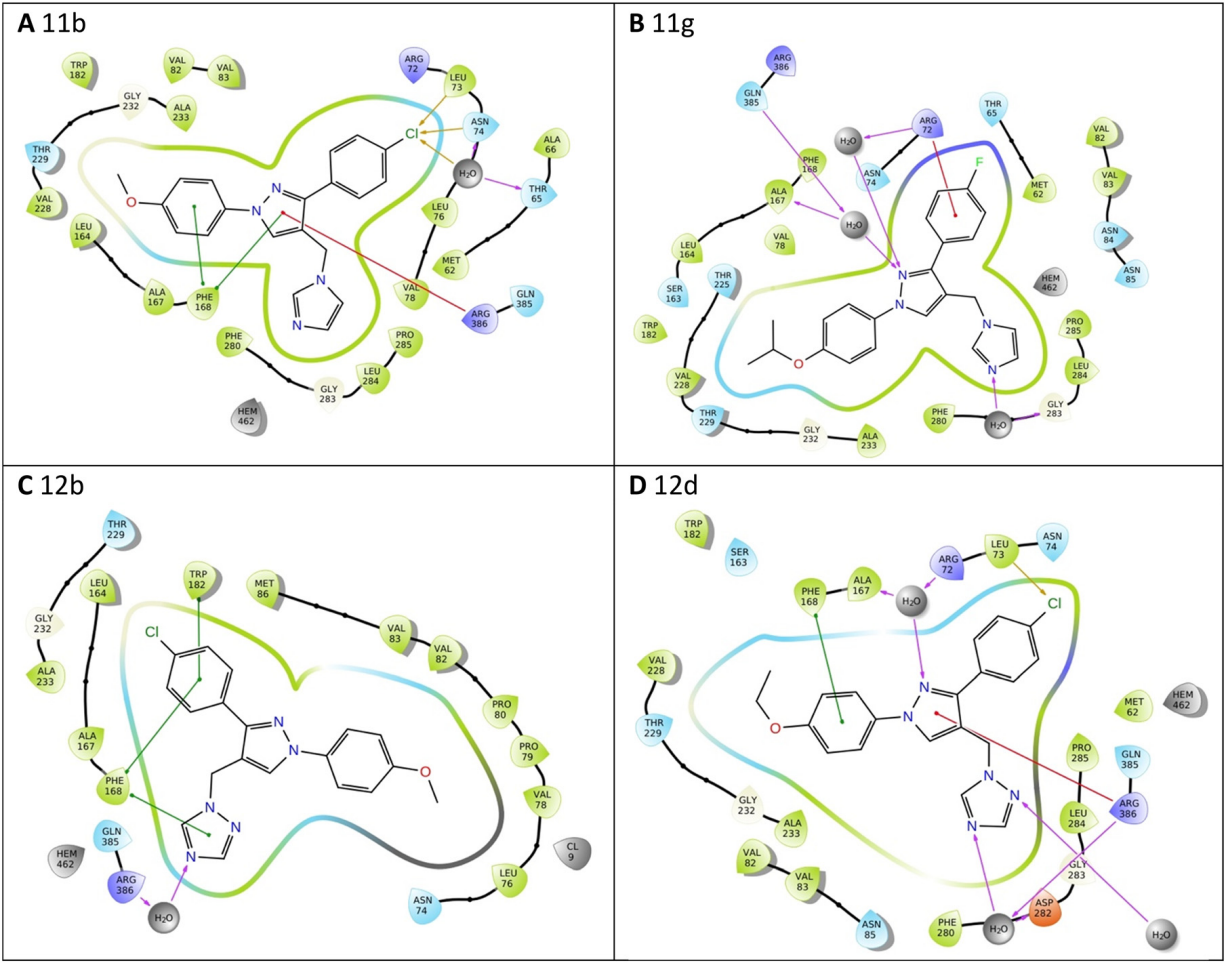
Exemplar 2D ligand interactions with Mtb CYP121A1. (A) 11b, (B) 11g, (C) 12b and (D) 12d.

All the pyrazole derivatives were positioned in a comparable position occupied by cYY, to the side of the haem group (Fig. 7). Water-mediated binding interactions were observed with either the imidazole, triazole or pyrazole ring with Gln385/Arg386 and additional hydrophobic/π–π stacking between one of the benzene rings of the pyrazole and Phe168 (*e.g.*11b, 12b and 12d) (Fig. 8). A halide interaction was observed for some of the pyrazole derivatives with Leu73 (*e.g.*11b and 12d) and additional water mediated and multiple hydrophobic interactions (Fig. 8).

## Conclusions

In this pyrazole series, the imidazole derivatives were generally more active against Mtb with optimal activity observed for the longer/branched chain alkoxy substitutions (*e.g.*11f and 11g). For the triazole derivatives, the compounds with a chlorobenzene ring were more active against Mtb compared with triazole derivatives containing a fluorobenzene ring. Generally, UV-vis spectral binding assay, which measures binding to the haem, either direct (type II) or indirect (type I through displacement of the 6th axial water ligand of the haem, confirmed type II binding for the imidazole derivatives and reflected Mtb inhibitory (MIC) activity. Compared with lead imidazole compounds (Fig. 2), binding affinity was either comparable or improved but the Mtb inhibitory activity was reduced (*e.g.*Fig. 2, lead compound R = Pr MIC 0.78 μg mL^−1^ compared with propyloxy derivative 11f MIC 4.0 μg mL^−1^). Possibly the reduction in lipophilicity compared with the lead compounds, owing to addition of the oxygen from the alkoxy substitution may be sufficient to reduce uptake across the Mtb cell membrane.

Previously we prepared methoxy derivatives with both imidazole and triazole haem binding groups (P1 and P2, Table 4), both of which had MIC_90_ = 25 μg mL^−1^.^6^ Comparison with the methoxy derivatives (11a–b, 12a–b) with the halide substitutions in the second aryl ring showed improved antimycobacterial activity), which may also be related to the increase in lipophilicity and would be consistent with our previous studies.^6–9^

**Table 4:** MIC_90_ of methoxy compounds against Mtb H37Rv

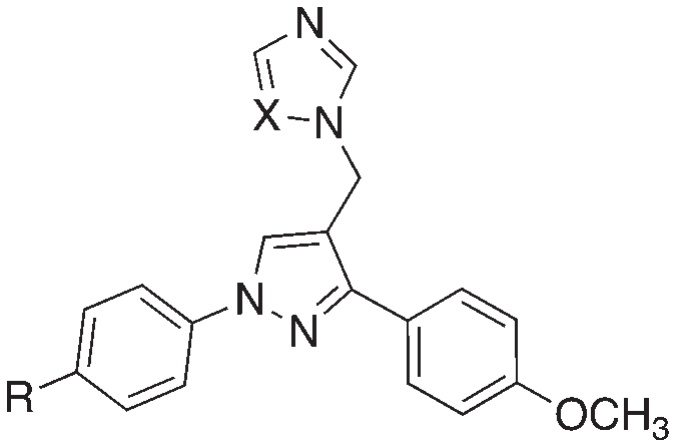				
Compd	R	X	MIC_90_^a^ (μg mL^−1^)	cLog *P*^b^
P1	H	CH	25	3.16
11a	F	CH	4.3	3.32
11b	Cl	CH	6.0	3.72
P2	H	N	25	2.67
12a	F	N	12.8	2.83
12b	Cl	N	4.4	3.23

a Results are the average of two independent experiments.

b Crippen's fragmentation.^15^

Only one triazole (12b) had a measurable binding affinity using the UV-vis spectral assay, which did not correlate with the Mtb inhibitory activity observed (MIC). The use of the ^19^F NMR assay to measure changes observed in the FG loop upon binding of ligands using BTFA ^19^F labelled CYP121_S171C, provided support that for the triazole derivatives (and imidazole derivatives) ligand coordination occurs in the substrate-binding pocket and in all cases with tighter binding than cYY (Fig. 5). Computational studies also support the pyrazole compounds binding in the substrate (cYY) binding pocket (Fig. 7) at the side of the haem rather than perpendicular, which may for some derivatives mean that binding or displacement of the haem water ligand may not occur even though binding in the substrate and access channel is efficient. If no perturbation of the haem/water ligand occurs this reduces the suitability of the UV-vis absorption method and highlights the importance of the results of the ^19^F NMR assay, which support the use of this method as an additional qualitative assay to determine ligand-CYP121A1 binding. This manuscript describes active triazole derivatives with good MIC values (some comparable with or better than the equivalent imidazole), which was not previously observed^6–9^ and, having established 1D ^19^F-NMR spectroscopy as an orthogonal strategy to evaluate ligand binding independent of perturbations at the haem, binding can now be confirmed. Further studies will focus on pyrazole derivative containing a triazole or alternative N-containing heterocycles (*e.g.* pyridines) as the haem binding moiety, as well as selectivity (human CYP panel) and toxicity assays.

## Experimental

### General

All chemicals, reagents and solvents were purchased from Sigma-Aldrich, Alfa Aesar, VWR, Acros and Fluka. Solvents were dried prior to use over molecular sieves (4 Å). For column chromatography, a glass column was slurry packed in the appropriate eluent with silica gel (Fluka Kieselgel 60). TLC was performed on pre-coated silica plates (dimension 20 × 20 cm) (ALUGRAM® SIL G/UV_254_) with visualisation *via* UV light (254 nm). Melting points were determined on an electrothermal instrument (Gallenkamp) and were uncorrected. ^1^H, ^13^C, and ^19^F NMR spectra were recorded on a Bruker Advance DP500 spectrometer operating at 500, 125 and 470 MHz, respectively. Chemical shifts are given in parts per million (ppm) relative to the internal standard tetramethylsilane (Me_4_Si). Elemental analysis was performed by MEDAC Ltd (Chobham, Surrey, UK) and HPLC-HRMS was performed at the Department of Pharmacy & Pharmacology, University of Bath, Bath, UK. on a Zorbax Eclipse Plus C18 Rapid Resolution 2.1 × 50 mm, 1.8 μm particle size using a 7.5 minutes gradient method 5 : 95 v/v water : methanol with 0.1% formic acid as additive. Experimental data for compounds 7, 8, 9 and 10 are provided in ESI.†

### Chemistry

#### General procedure for the preparation of imidazole and triazole derivatives (11 and 12) from the chloride derivatives (10)

To a stirred suspension of K_2_CO_3_ (4 equiv.) in dry CH_3_CN (2.5 mL mmol^−1^) was added imidazole or triazole (4 equiv.). The reaction mixture was heated at 45 °C for 1 h. After cooling to room temperature, the chloro derivative (10) (1 equiv.) was added and the reaction mixture heated at 70 °C overnight. The solvent was evaporated under reduced pressure and the resulting mixture was diluted with EtOAc (50 mL) and washed with brine (3 × 20 mL) and H_2_O (3 × 20 mL). The organic layer was dried (MgSO_4_) and evaporated under reduced pressure to give the crude product, which was purified by gradient column chromatography (CH_2_Cl_2_–MeOH).

#### 4-((1*H*-Imidazol-1-yl)methyl)-1-(4-fluorophenyl)-3-(4-methoxyphenyl)-1*H*-pyrazole (11a)

Prepared from 4-(chloromethyl)-1-(4-fluorophenyl)-3-(4-methoxyphenyl)-1*H*-pyrazole (10a) (0.5 g, 1.6 mmol). The pure compound was eluted with CH_2_Cl_2_–MeOH 97 : 3 v/v and obtained as a light brown solid. Yield 0.39 g (70%), m.p. 150–152 °C, TLC (CH_2_Cl_2_–MeOH 95 : 5 v/v) R*f* 0.50. ^1^H NMR (DMSO-d_6_): *δ* 8.49 (s, 1H, pyrazole), 7.90 (m, 2H, Ar), 7.65 (s, 1H, imidazole), 7.56 (d, *J* = 8.5 Hz, 2H, Ar), 7.37 (m, 2H, Ar), 7.12 (s, 1H, imidazole), 7.02 (d, *J* = 9.0 Hz, 2H, Ar), 6.89 (s, 1H, imidazole), 5.27 (s, 2H, CH_2_-imidazole), 3.81 (s, 3H, OCH_3_). ^19^F NMR: (DMSO-d_6_): −116.49 (F–Ar). ^13^C NMR (DMSO-d_6_): *δ* 159.81 and 161.74 (d, ^1^*J*_CF_ = 242.7 Hz, C–F, Ar), 159.76 (C, Ar), 150.69 (C, pyrazole), 137.45 (CH, imidazole), 136.41 (d, ^4^*J*_CF_ = 2.5 Hz, C, Ar), 130.14 (CH, pyrazole), 129.22 (2 × CH, Ar), 129.08 (CH, imidazole), 125.20 (C, Ar), 120.76 and 120.82 (d, ^3^*J*_CF_ = 7.5 Hz, 2 × CH, Ar), 119.61 (CH, imidazole), 116.95 (C, pyrazole), 116.67 and 116.85 (d, ^2^*J*_CF_ = 22.6 Hz, 2 × CH, Ar), 114.61 (2 × CH, Ar), 55.64 (OCH_3_), 40.95 (CH_2_). Anal. calcd. for C_20_H_17_FN_4_O (348.3814): C 68.59%, H 4.89%, N 15.99%. Found: C 68.54%, H 4.73%, N 16.07%.

#### 4-((1*H*-Imidazol-1-yl)methyl)-1-(4-chlorophenyl)-3-(4-methoxyphenyl)-1*H*-pyrazole (11b)

Prepared from 4-(chloromethyl)-1-(4-chlorophenyl)-3-(4-methoxyphenyl)-1*H*-pyrazole (10b) (0.35 g, 1.05 mmol). The pure compound was eluted with CH_2_Cl_2_–MeOH 97 : 3 v/v and obtained as a light orange solid. Yield 0.23 g (61%), m.p. 136–138 °C, TLC (CH_2_Cl_2_–MeOH 95 : 5 v/v) R*f* 0.44. ^1^H NMR (DMSO-d_6_): *δ* 8.53 (s, 1H, pyrazole), 7.90 (d, *J* = 9.0 Hz, 2H, Ar), 7.65 (s, 1H, imidazole), 7.58 (d, *J* = 9.0 Hz, 2H, Ar), 7.56 (d, *J* = 9.0 Hz, 2H, Ar), 7.12 (s, 1H, imidazole), 7.02 (d, *J* = 9.0 Hz, 2H, Ar), 6.88 (s, 1H, imidazole), 5.28 (s, 2H, CH_2_-imidazole), 3.81 (s, 3H, CH_2_CH_3_). ^13^C NMR (DMSO-d_6_): *δ* 159.83 (C, Ar), 150.94 (C, pyrazole), 138.60 (C, Ar), 137.47 (CH, imidazole), 130.86 (C, Ar), 130.10 (CH, imidazole), 129.96 (2 × CH, Ar), 129.25 (2 × CH, Ar), 129.08 (CH, pyrazole), 125.06 (C, pyrazole), 120.33 (2 × CH, Ar) 119.63 (CH, imidazole), 117.32 (C, Ar), 114.63 (2 × CH, Ar), 55.65 (CH_3_), 40.94 (CH_2_). Anal. calcd. for C_20_H_17_ClN_4_O (364.8330): C 65.84%, H 4.70%, N 15.35%. Found: C 65.97%, H 4.59%, N 15.38%.

#### 4-((1*H*-Imidazol-1-yl)methyl)-3-(4-ethoxyphenyl)-1-(4-fluorophenyl)-1*H*-pyrazole (11c)

Prepared from 4-(chloromethyl)-3-(4-ethoxyphenyl)-1-(4-fluorophenyl)-1*H*-pyrazole (10c) (0.295 g, 0.89 mmol). The pure compound was eluted with CH_2_Cl_2_–MeOH 98 : 2 v/v and obtained as a light brown solid. Yield 0.146 g (45%), m.p. 118–120 °C, TLC (CH_2_Cl_2_–MeOH 95 : 5 v/v) R*f* 0.47. ^1^H NMR (DMSO-d_6_): *δ* 8.48 (s, 1H, pyrazole), 7.90 (dd, *J* = 4.5, 9.0 Hz, 2H, Ar), 7.64 (s, 1H, imidazole), 7.55 (d, *J* = 9.0 Hz, 2H, Ar), 7.37 (t, *J* = 8.5 Hz, 2H, Ar), 7.12 (s, 1H, imidazole), 7.0 (d, *J* = 8.5 Hz, 2H, Ar), 6.88 (s, 1H, imidazole), 5.27 (s, 2H, CH_2_-imidazole), 4.07 (q, *J* = 7.0 Hz, 2H, CH_2_CH_3_), 1.35 (t, *J* = 7.0 Hz, 3H, CH_2_CH_3_). ^19^F NMR: (DMSO-d_6_): −116.52 (F–Ar). ^13^C NMR (DMSO-d_6_): *δ* 159.80 and 161.73 (d, ^1^*J*_CF_ = 242.7 Hz, C–F, Ar), 159.04 (C, Ar), 150.71 (C, pyrazole), 136.42 (d, ^4^*J*_CF_ = 2.5 Hz, C, Ar), 130.10 (CH, pyrazole), 129.22 (2 × CH, Ar), 129.08 (CH, imidazole), 125.06 (C, Ar), 120.74 and 120.81 (d, ^3^*J*_CF_ = 8.8 Hz, 2 × CH, Ar), 119.61 (CH, imidazole), 116.96 (C, pyrazole), 116.67 and 116.85 (d, ^2^*J*_CF_ = 22.6 Hz, 2 × CH, Ar), 115.04 (2 × CH, Ar), 63.57 (CH_2_CH_3_), 40.96 (CH_2_), 15.12 (CH_2_CH_3_). Anal. calcd. for C_21_H_19_FN_4_O (362.4084): C 69.25%, H 5.26%, N 15.38%. Found: C 68.86%, H 5.06%, N 15.16%.

#### 4-((1*H*-Imidazol-1-yl)methyl)-1-(4-chlorophenyl)-3-(4-ethoxyphenyl)-1*H*-pyrazole (11d)

Prepared from 4-(chloromethyl)-1-(4-chlorophenyl)-3-(4-ethoxyphenyl)-1*H*-pyrazole (10d) (0.17 g, 0.49 mmol). The pure compound was eluted with CH_2_Cl_2_–MeOH 97.5 : 2.5 v/v and obtained as a white solid. Yield 0.04 g (22%), m.p. 124–126 °C, TLC (CH_2_Cl_2_–MeOH 95 : 5 v/v) R*f* 0.55. ^1^H NMR (DMSO-d_6_): *δ* 8.53 (s, 1H, pyrazole), 7.90 (d, *J* = 9.0 Hz, 2H, Ar), 7.64 (s, 1H, imidazole), 7.58 (d, *J* = 9.0 Hz, 2H, Ar), 7.56 (d, *J* = 8.8 Hz, 2H, Ar), 7.12 (s, 1H, imidazole), 7.01 (d, *J* = 8.8 Hz, 2H, Ar), 6.88 (s, 1H, imidazole), 5.28 (s, 2H, CH_2_-imidazole), 4.08 (q, *J* = 7.0 Hz, 2H, CH_2_CH_3_), 1.35 (t, *J* = 7.0 Hz, 3H, CH_2_CH_3_). ^13^C NMR (DMSO-d_6_): *δ* 159.11 (C, Ar), 150.95 (C, pyrazole), 138.61 (C, Ar), 137.47 (CH, imidazole), 130.83 (C, Ar), 130.06 (CH, imidazole), 129.96 (2 × CH, Ar), 129.24 (2 × CH, Ar), 129.09 (CH, pyrazole), 124.93 (C, pyrazole), 120.31 (2 × CH, Ar) 119.63 (CH, imidazole), 117.34 (C, Ar), 115.06 (2 × CH, Ar), 63.58 (CH_2_CH_3_), 40.96 (CH_2_), 15.11 (CH_2_CH_3_). HPLC: 97.8%, RT = 4.48 min. HRMS (ESI) *m*/*z*: calculated, 401.1145 [M + Na]^+^, Found, 401.1147 [M + Na]^+^.

#### 4-((1*H*-Imidazol-1-yl)methyl)-1-(4-fluorophenyl)-3-(4-propoxyphenyl)-1*H*-pyrazole (11e)

Prepared from 4-(chloromethyl)-1-(4-fluorophenyl)-3-(4-propoxyphenyl)-1*H*-pyrazole (10e) (0.30 g, 0.87 mmol). The pure compound was eluted with CH_2_Cl_2_–MeOH 98 : 2 v/v and obtained as a light orange solid. Yield 0.20 g (66%), m.p. 120–122 °C, TLC (CH_2_Cl_2_–MeOH 95 : 5 v/v) R*f* 0.51. ^1^H NMR (DMSO-d_6_): *δ* 8.48 (s, 1H, pyrazole), 7.90 (m, 2H, Ar), 7.64 (s, 1H, imidazole), 7.56 (d, *J* = 9.0 Hz, 2H, Ar), 7.36 (t, *J* = 9.0 Hz, 2H, Ar), 7.12 (s, 1H, imidazole), 7.02 (d, *J* = 8.50 Hz, 2H, Ar), 6.88 (s, 1H, imidazole), 5.27 (s, 2H, CH_2_-imidazole), 3.97 (t, *J* = 6.5 Hz, 2H, OCH_2_), 1.76 (m, 2H, CH_2_), 1.0 (t, *J* = 7.0 Hz, 3H, CH_3_). ^19^F NMR: (DMSO-d_6_): −116.51 (F–Ar). ^13^C NMR (DMSO-d_6_): *δ* 159.80 and 161.73 (d, ^1^*J*_CF_ = 242.7 Hz, C–F, Ar), 159.20 (C, Ar), 150.71 (C, pyrazole), 137.45 (CH, imidazole), 136.42 (d, ^4^*J*_CF_ = 2.5 Hz, C, Ar), 130.10 (CH, pyrazole), 129.21 (2 × CH, Ar), 129.08 (CH, imidazole), 125.05 (C, Ar), 120.81 and 120.74 (d, ^3^*J*_CF_ = 8.8 Hz, 2 × CH, Ar), 119.62 (CH, imidazole), 116.96 (C, pyrazole), 116.67 and 116.85 (d, ^2^*J*_CF_ = 22.6 Hz, 2 × CH, Ar), 115.08 (2 × CH, Ar), 69.46 (OCH_2_), 40.96 (imidazole-CH_2_), 22.50 (CH_2_), 10.87 (CH_3_). Anal. calcd. for C_22_H_21_FN_4_O (376.4354): C 70.20%, H 5.62%, N 14.88%. Found: C 70.00%, H 5.54%, N 14.97%.

#### 4-((1*H*-Imidazol-1-yl)methyl)-1-(4-chlorophenyl)-3-(4-propoxyphenyl)-1*H*-pyrazole (11f)

Prepared from 4-(chloromethyl)-1-(4-chlorophenyl)-3-(4-propoxyphenyl)-1*H*-pyrazole (10f) (0.30 g, 0.83 mmol). The pure compound was eluted with CH_2_Cl_2_–MeOH 98 : 2 v/v and obtained as a light brown solid. Yield 0.20 g (63%), m.p. 90–92 °C, TLC (CH_2_Cl_2_–MeOH 95 : 5 v/v) R*f* 0.60. ^1^H NMR (DMSO-d_6_): *δ* 8.53 (s, 1H, pyrazole), 7.91 (d, *J* = 9.0 Hz, 2H, Ar), 7.65 (s, 1H, imidazole), 7.58 (d, *J* = 9.0 Hz, 2H, Ar), 7.56 (d, *J* = 8.50 Hz, 2H, Ar), 7.12 (s, 1H, imidazole), 7.02 (d, *J* = 9.0 Hz, 2H, Ar), 6.89 (s, 1H, imidazole), 5.28 (s, 2H, CH_2_-imidazole), 3.98 (t, *J* = 6.5 Hz, 2H, OCH_2_), 1.76 (m, 2H, CH_2_), 1.0 (t, *J* = 7.0 Hz, 3H, CH_3_). ^13^C NMR (DMSO-d_6_): *δ* 159.27 (C, Ar), 150.95 (C, pyrazole), 138.61 (C, Ar), 137.48 (CH, imidazole), 130.83 (C, Ar), 130.05 (CH, imidazole), 129.95 (2 × CH, Ar), 129.24 (2 × CH, Ar), 129.10 (CH, pyrazole), 124.93 (C, pyrazole), 120.30 (2 × CH, Ar) 119.63 (CH, imidazole), 117.34 (C, Ar), 115.09 (2 × CH, Ar), 69.46 (OCH_2_), 40.97 (imidazole-CH_2_), 22.50 (CH_2_), 10.87 (2 × CH_3_). Anal. calcd. for C_22_H_21_ClN_4_O (392.1404): C 67.26%, H 5.39%, N 14.25%. Found: C 67.25%, H 5.19%, N 14.27%.

#### 4-((1*H*-Imidazol-1-yl)methyl)-1-(4-fluorophenyl)-3-(4-isopropoxyphenyl)-1*H*-pyrazole (11g)

Prepared from 4-(chloromethyl)-1-(4-fluorophenyl)-3-(4-isopropoxyphenyl)-1*H*-pyrazole (10g) (0.15 g, 0.44 mmol). The pure compound was eluted with CH_2_Cl_2_–MeOH 98.5 : 1.5 v/v and obtained as a light brown solid. Yield 0.096 g (58%), m.p. 96–98 °C, TLC (CH_2_Cl_2_–MeOH 95 : 5 v/v) R*f* 0.44. ^1^H NMR (DMSO-d_6_): *δ* 8.48 (s, 1H, pyrazole), 7.90 (m, 2H, Ar), 7.65 (s, 1H, imidazole), 7.54 (d, *J* = 9.0 Hz, 2H, Ar), 7.36 (t, *J* = 9.0 Hz, 2H, Ar), 7.13 (s, 1H, imidazole), 7.0 (d, *J* = 9.0 Hz, 2H, Ar), 6.89 (s, 1H, imidazole), 5.27 (s, 2H, CH_2_-imidazole), 4.67 (m, 1H, CH), 1.30 (d, *J* = 6.0 Hz, 6H, 2CH_3_). ^19^F NMR: (DMSO-d_6_): −116.52 (F–Ar). ^13^C NMR (DMSO-d_6_): *δ* 159.80 and 161.73 (d, ^1^*J*_CF_ = 242.7 Hz, C–F, Ar), 157.99 (C, Ar), 150.72 (C, pyrazole), 137.46 (CH, imidazole), 136.43 (d, ^4^*J*_CF_ = 2.5 Hz, C, Ar), 130.06 (CH, pyrazole), 129.26 (2 × CH, Ar), 129.08 (CH, imidazole), 124.89 (C, Ar), 120.79 and 120.72 (d, ^3^*J*_CF_ = 8.8 Hz, 2 × CH, Ar), 119.63 (CH, imidazole), 116.96 (C, pyrazole), 116.85 and 116.67 (d, ^2^*J*_CF_ = 22.6 Hz, 2 × CH, Ar), 116.12 (2 × CH, Ar), 69.67 (CH), 40.97 (CH_2_), 22.29 (2 × CH_3_). Anal. calcd. for C_22_H_21_FN_4_O (376.4354): C 70.20%, H 5.62%, N 14.88%. Found: C 69.84%, H 5.48%, N 14.83%.

#### 4-((1*H*-Imidazol-1-yl)methyl)-1-(4-chlorophenyl)-3-(4-isopropoxyphenyl)-1*H*-pyrazole (11h)

Prepared from 4-(chloromethyl)-1-(4-chlorophenyl)-3-(4-isopropoxyphenyl)-1*H*-pyrazole (10h) (0.30 g, 0.83 mmol). The pure compound was eluted with CH_2_Cl_2_–MeOH 97.5 : 2.5 v/v and obtained as a beige solid. Yield 0.30 g (94%), m.p. 98–100 °C, TLC (CH_2_Cl_2_–MeOH 95 : 5 v/v) R*f* 0.45. ^1^H NMR (DMSO-d_6_): *δ* 8.52 (s, 1H, pyrazole), 7.90 (d, *J* = 9.0 Hz, 2H, Ar), 7.65 (s, 1H, imidazole), 7.58 (d, *J* = 9.0 Hz, 2H, Ar), 7.55 (d, *J* = 9.0 Hz, 2H, Ar), 7.13 (s, 1H, imidazole), 7.0 (d, *J* = 8.50 Hz, 2H, Ar), 6.89 (s, 1H, imidazole), 5.28 (s, 2H, CH_2_-imidazole), 4.67 (m, 1H, CH), 1.30 (d, *J* = 6.0 Hz, 6H, 2 × CH_3_). ^13^C NMR (DMSO-d_6_): *δ* 158.06 (C, Ar), 150.96 (C, pyrazole), 138.61 (C, Ar), 137.48 (CH, imidazole), 130.82 (C, Ar), 130.01 (CH, imidazole), 129.95 (2 × CH, Ar), 129.29 (2 × CH, Ar), 129.10 (CH, pyrazole), 124.76 (C, pyrazole), 120.29 (2 × CH, Ar) 119.64 (CH, imidazole), 117.34 (C, Ar), 116.12 (2 × CH, Ar), 69.68 (CH), 40.98 (CH_2_), 22.28 (2 × CH_3_). Anal. calcd. for C_22_H_21_ClN_4_O (392.1404): C 67.26%, H 5.39%, N 14.26%. Found: C 67.03%, H 5.16%, N 14.21%.

#### 1-((1-(4-Fluorophenyl)-3-(4-methoxyphenyl)-1*H*-pyrazol-4-yl)methyl)-1*H*-1,2,4-triazole (12a)

Prepared from 4-(chloromethyl)-1-(4-fluorophenyl)-3-(4-methoxyphenyl)-1*H*-pyrazole (10a) (0.44 g, 1.3 mmol). The pure compound was eluted with CH_2_Cl_2_–MeOH 97.5 : 2.5 v/v and obtained as an orange solid. Yield 0.26 g (58%), m.p. 150–152 °C, TLC (CH_2_Cl_2_–MeOH 95 : 5 v/v) R*f* 0.63. ^1^H NMR (DMSO-d_6_): *δ* 8.54 (s, 1H, triazole), 8.54 (s, 1H, pyrazole), 7.99 (s, 1H, triazole), 7.90 (m, 2H, Ar), 7.67 (d, *J* = 8.50 Hz, 2H, Ar), 7.36 (m, 2H, Ar), 7.04 (d, *J* = 9.0 Hz, 2H, Ar), 5.48 (s, 2H, CH_2_-triazole), 3.81 (s, 3H, CH_3_). ^19^F NMR: (DMSO-d_6_): −116.42 (F–Ar). ^13^C NMR (DMSO-d_6_): *δ* 161.77 and 159.83 (d, ^1^*J*_CF_ = 242.7 Hz, C–F, Ar), 159.80 (C, Ar), 151.99 (CH, triazole), 150.89 (C, pyrazole), 144.36 (CH, triazole), 136.39 and 136.36 (d, ^4^*J*_CF_ = 2.5 Hz, C, Ar), 130.40 (CH, pyrazole), 129.37 (2 × CH, Ar), 125.04 (C, pyrazole), 120.83 and 120.76 (d, ^3^*J*_CF_ = 8.7 Hz, 2 × CH, Ar), 116.86 and 116.68 (d, ^2^*J*_CF_ = 22.6 Hz, 2 × CH, Ar), 115.69 (C, Ar), 114.59 (2 × CH, Ar), 55.65 (CH_3_), 43.81 (CH_2_). Anal. calcd. for C_19_H_16_FN_5_O (349.3694): C 64.99%, H 4.59%, N 19.94%. Found: C 64.67%, H 4.41%, N 19.95%.

#### 1-((1-(4-Chlorophenyl)-3-(4-methoxyphenyl)-1*H*-pyrazol-4-yl)methyl)-1*H*-1,2,4-triazole (12b)

Prepared from 4-(chloromethyl)-1-(4-chlorophenyl)-3-(4-methoxyphenyl)-1*H*-pyrazole (10b) (0.35 g, 1.05 mmol). The pure compound was eluted with CH_2_Cl_2_–MeOH 97 : 3 v/v and obtained as a light orange solid. Yield 0.32 g (60%), m.p. 124–126 °C, TLC (CH_2_Cl_2_–MeOH 95 : 5 v/v) R*f* 0.65. ^1^H NMR (DMSO-d_6_): *δ* 8.59 (s, 1H, triazole), 8.54 (s, 1H, pyrazole), 7.99 (s, 1H, triazole), 7.90 (d, *J* = 9.0 Hz, 2H, Ar), 7.67 (d, *J* = 8.5 Hz, 2H, Ar), 7.58 (d, *J* = 9.0 Hz, 2H, Ar), 7.03 (d, *J* = 9.0 Hz, 2H, Ar), 5.48 (s, 2H, CH_2_-triazole), 3.81 (s, 3H, CH_3_). ^13^C NMR (DMSO-d_6_): *δ* 159.87 (C, Ar), 152.01 (CH, triazole), 151.13 (C, pyrazole), 144.39 (CH, triazole), 138.58 (C, Ar), 130.90 (C, Ar), 130.37 (CH, pyrazole), 129.96 (2 × CH, Ar), 129.39 (2 × CH, Ar), 124.92 (C, pyrazole), 120.32 (2 × CH, Ar), 116.06 (C, Ar), 114.61 (2 × CH, Ar), 55.66 (CH_3_), 43.79 (CH_2_). Anal. calcd. for C_19_H_16_ClN_5_O (365.8210): C 62.08%, H 4.39%, N 19.05%. Found: C 61.77%, H 4.14%, N 19.00%.

#### 1-((3-(4-Ethoxyphenyl)-1-(4-fluorophenyl)-1*H*-pyrazol-4-yl)methyl)-1*H*-1,2,4-triazole (12c)

Prepared from 4-(chloromethyl)-3-(4-ethoxyphenyl)-1-(4-fluorophenyl)-1*H*-pyrazole (10c) (0.40 g, 1.2 mmol). The pure compound was eluted with CH_2_Cl_2_–MeOH 98.5 : 1.5 v/v and obtained as a light brown solid. Yield 0.23 g (52%), m.p. 140–142 °C, TLC (CH_2_Cl_2_–MeOH 95 : 5 v/v) R*f* 0.56. ^1^H NMR (DMSO-d_6_): *δ* 8.53 (s, 1H, pyrazole), 8.53 (s, 1H, triazole), 7.98 (s, 1H, triazole), 7.90 (m, 2H, Ar), 7.66 (d, *J* = 9.0 Hz, 2H, Ar), 7.36 (t, *J* = 8.50 Hz, 2H, Ar), 7.02 (d, *J* = 9.0 Hz, 2H, Ar), 5.48 (s, 2H, CH_2_-triazole), 4.08 (q, *J* = 7.0 Hz, 2H, OCH_2_), 1.35 (t, *J* = 7.0 Hz, 3H, CH_3_). ^19^F NMR: (DMSO-d_6_): −116.44 (F–Ar). ^13^C NMR (DMSO-d_6_): *δ* 161.76 and 159.82 (d, ^1^*J*_CF_ = 243.9 Hz, C–F, Ar), 159.08 (C, Ar), 151.99 (CH, triazole), 150.91 (C, pyrazole), 144.36 (CH, triazole), 136.39 and 136.37 (d, ^4^*J*_CF_ = 2.5 Hz, C, Ar), 130.37 (CH, pyrazole), 129.36 (2 × CH, Ar), 124.90 (C, pyrazole), 120.81 and 120.74 (d, ^3^*J*_CF_ = 8.80 Hz, 2 × CH, Ar), 116.85 and 116.67 (d, ^2^*J*_CF_ = 22.6 Hz, 2 × CH, Ar), 115.68 (C, Ar), 115.02 (2 × CH, Ar), 63.57 (OCH_2_), 43.81 (triazole-CH_2_), 15.11 (CH_3_). Anal. calcd. for C_20_H_18_FN_5_O (363.3964): C 66.10%, H 4.99%, N 19.26%. Found: C 66.21%, H 4.87%, N 19.17%.

#### 1-((1-(4-Chlorophenyl)-3-(4-ethoxyphenyl)-1*H*-pyrazol-4-yl)methyl)-1*H*-1,2,4-triazole (12d)

Prepared from 4-(chloromethyl)-1-(4-chlorophenyl)-3-(4-ethoxyphenyl)-1*H*-pyrazole (10d) (0.49 g, 1.41 mmol). The pure compound was eluted with CH_2_Cl_2_–MeOH 98 : 2 v/v and obtained as a light brown solid. Yield 0.32 g (60%), m.p. 122–124 °C, TLC (CH_2_Cl_2_–MeOH 95 : 5 v/v) R*f* 0.49. ^1^H NMR (DMSO-d_6_): *δ* 8.58 (s, 1H, triazole), 8.54 (s, 1H, pyrazole), 7.98 (s, 1H, triazole), 7.90 (d, *J* = 8.5 Hz, 2H, Ar), 7.66 (d, *J* = 9.0 Hz, 2H, Ar), 7.57 (d, *J* = 9.0 Hz, 2H, Ar), 7.01 (d, *J* = 9.0 Hz, 2H, Ar), 5.48 (s, 2H, CH_2_-triazole), 4.08 (q, *J* = 7.0 Hz, 2H, CH_2_CH_3_), 1.35 (t, *J* = 6.5 Hz, 3H, CH_2_CH_3_). ^13^C NMR (DMSO-d_6_): *δ* 159.15 (C, Ar), 152.01 (CH, triazole), 151.15 (C, pyrazole), 144.39 (CH, triazole), 138.58 (C, Ar), 130.89 (C, Ar), 130.34 (CH, pyrazole), 129.96 (2 × CH, Ar), 129.39 (2 × CH, Ar), 124.78 (C, pyrazole), 120.31 (2 × CH, Ar), 116.05 (C, Ar), 115.04 (2 × CH, Ar), 63.58 (CH_2_CH_3_), 43.80 (CH_2_), 15.11 (CH_2_CH_3_). Anal. calcd. for C_20_H_18_ClN_5_O (379.8480): C 63.24%, H 4.78%, N 18.43%. Found: C 62.91%, H 4.63%, N 18.29%.

#### 1-((1-(4-Fluorophenyl)-3-(4-propoxyphenyl)-1*H*-pyrazol-4-yl)methyl)-1*H*-1,2,4-triazole (12e)

Prepared from 4-(chloromethyl)-1-(4-fluorophenyl)-3-(4-propoxyphenyl)-1*H*-pyrazole (10e) (0.30 g, 0.87 mmol). The pure compound was eluted with CH_2_Cl_2_–MeOH 98.5 : 1.5 v/v and obtained as a light orange solid. Yield 0.185 g (58%), m.p. 120–122 °C, TLC (CH_2_Cl_2_–MeOH 95 : 5 v/v) R*f* 0.51. ^1^H NMR (DMSO-d_6_): *δ* 8.53 (s, 1H, pyrazole), 8.53 (s, 1H, triazole), 7.98 (s, 1H, triazole), 7.90 (m, 2H, Ar), 7.66 (d, *J* = 9.0 Hz, 2H, Ar), 7.36 (t, *J* = 9.0 Hz, 2H, Ar), 7.03 (d, *J* = 9.0 Hz, 2H, Ar), 5.48 (s, 2H, CH_2_-triazole), 3.98 (t, *J* = 6.5 Hz, 2H, OCH_2_), 1.76 (m, 2H, CH_2_), 1.0 (t, *J* = 7.0 Hz, 3H, CH_3_). ^19^F NMR: (DMSO-d_6_): −116.45 (F–Ar). ^13^C NMR (DMSO-d_6_): *δ* 161.76 and 159.82 (d, ^1^*J*_CF_ = 243.9 Hz, C–F, Ar), 159.24 (C, Ar), 151.99 (CH, triazole), 150.91 (C, pyrazole), 144.36 (CH, triazole), 136.39 and 136.37 (d, ^4^*J*_CF_ = 2.5 Hz, C, Ar), 130.37 (CH, pyrazole), 129.36 (2 × CH, Ar), 124.90 (C, pyrazole), 120.81 and 120.75 (d, ^3^*J*_CF_ = 7.5 Hz, 2 × CH, Ar), 116.86 and 116.67 (d, ^2^*J*_CF_ = 23.9 Hz, 2 × CH, Ar), 115.68 (C, Ar), 115.06 (2 × CH, Ar), 69.46 (OCH_2_), 43.82 (triazole-CH_2_), 22.50 (CH_2_), 10.88 (CH_3_). Anal. calcd. for C_21_H_20_FN_5_O (377.4234): C 66.83%, H 5.34%, N 18.55%. Found: C 66.67%, H 5.20%, N 18.67%.

#### 1-((1-(4-Chlorophenyl)-3-(4-propoxyphenyl)-1*H*-pyrazol-4-yl)methyl)-1*H*-1,2,4-triazole (12f)

Prepared from 4-(chloromethyl)-1-(4-chlorophenyl)-3-(4-propoxyphenyl)-1*H*-pyrazole (10f) (0.3 g, 0.83 mmol). The pure compound was eluted with CH_2_Cl_2_–MeOH 98.5 : 1.5 v/v and obtained as a light orange solid. Yield 0.23 g (72%), m.p. 114–116 °C, TLC (CH_2_Cl_2_–MeOH 95 : 5 v/v) R*f* 0.69. ^1^H NMR (DMSO-d_6_): *δ* 8.58 (s, 1H, triazole), 8.54 (s, 1H, pyrazole), 7.98 (s, 1H, triazole), 7.91 (d, *J* = 9.0 Hz, 2H, Ar), 7.67 (d, *J* = 9.0 Hz, 2H, Ar), 7.58 (d, *J* = 9.0 Hz, 2H, Ar), 7.03 (d, *J* = 9.0 Hz, 2H, Ar), 5.48 (s, 2H, CH_2_-triazole), 3.98 (t, *J* = 6.5 Hz, 2H, OCH_2_), 1.76 (m, 2H, CH_2_), 1.0 (t, *J* = 7.0 Hz, 3H, CH_3_). ^13^C NMR (DMSO-d_6_): *δ* 159.31 (C, Ar), 152.01 (CH, triazole), 151.15 (C, pyrazole), 144.40 (CH, triazole), 138.59 (C, Ar), 130.88 (C, Ar), 130.34 (CH, pyrazole), 129.96 (2v × CH, Ar), 129.38 (2 × CH, Ar), 124.78 (C, pyrazole), 120.31 (2 × CH, Ar), 116.06 (C, Ar), 115.08 (2 × CH, Ar), 69.47 (OCH_2_), 43.81 (triazole-CH_2_), 22.50 (CH_2_), 10.88v (CH_3_). Anal. calcd. for C_21_H_20_ClN_5_O (393.8750): C 64.04%, H 5.12%, N 17.77%. Found: C 63.94%, H 5.02%, N 17.87%.

#### 1-((1-(4-Fluorophenyl)-3-(4-isopropoxyphenyl)-1*H*-pyrazol-4-yl)methyl)-1*H*-1,2,4-triazole (12g)

Prepared from 4-(chloromethyl)-1-(4-fluorophenyl)-3-(4-isopropoxyphenyl)-1*H*-pyrazole (10g) (0.15 g, 0.44 mmol). The pure compound was eluted with CH_2_Cl_2_–MeOH 98.5 : 1.5 v/v and obtained as a light brown solid. Yield 0.088 g (53%), m.p. 118–120 °C, TLC (CH_2_Cl_2_–MeOH 95 : 5 v/v) R*f* 0.52. ^1^H NMR (DMSO-d_6_): *δ* 8.54 (s, 1H, pyrazole), 8.53 (s, 1H, triazole), 7.99 (s, 1H, triazole), 7.90 (m, 2H, Ar), 7.65 (d, *J* = 9.0 Hz, 2H, Ar), 7.36 (t, *J* = 9.0 Hz, 2H, Ar), 7.01 (d, *J* = 9.0 Hz, 2H, Ar), 5.48 (s, 2H, CH_2_-triazole), 4.67 (m, 1H, CH), 1.30 (d, *J* = 6.0 Hz, 6H, 2 × CH_3_). ^19^F NMR: (DMSO-d_6_): −116.46 (F–Ar). ^13^C NMR (DMSO-d_6_): *δ* 161.75 and 159.82 (d, ^1^*J*_CF_ = 242.7 Hz, C–F, Ar), 158.03 (C, Ar), 152.0 (CH, triazole), 150.93 (C, pyrazole), 144.37 (CH, triazole), 136.39 and 136.37 (d, ^4^*J*_CF_ = 3.77 Hz, C, Ar), 130.33 (CH, pyrazole), 129.41 (2 × CH, Ar), 124.74 (C, pyrazole), 120.80 and 120.73 (d, ^3^*J*_CF_ = 8.8 Hz, 2 × CH, Ar), 116.86 and 116.67 (d, ^2^*J*_CF_ = 23.9 Hz, 2 × CH, Ar), 116.10 (2 × CH, Ar), 115.69 (C, Ar), 69.66 (CH), 43.82 (CH_2_), 22.29 (CH_3_). Anal. calcd. for C_21_H_20_FN_5_O (377.4234): C 66.83%, H 5.34%, N 18.55%. Found: C 66.81%, H 5.16%, N 18.68%.

#### 1-((1-(4-Chlorophenyl)-3-(4-isopropoxyphenyl)-1*H*-pyrazol-4-yl)methyl)-1*H*-1,2,4-triazole (12h)

Prepared from 4-(chloromethyl)-1-(4-chlorophenyl)-3-(4-isopropoxyphenyl)-1*H*-pyrazole (10h) (0.3 g, 0.83 mmol). The pure compound was eluted with CH_2_Cl_2_–MeOH 97.5 : 2.5 v/v and obtained as a brown oil. Yield 0.26 g (81%), TLC (CH_2_Cl_2_–MeOH 95 : 5 v/v) R*f* 0.48. ^1^H NMR (DMSO-d_6_): *δ* 8.57 (s, 1H, triazole), 8.54 (s, 1H, pyrazole), 7.99 (s, 1H, triazole), 7.91 (d, *J* = 9.0 Hz, 2H, Ar), 7.66 (d, *J* = 9.0 Hz, 2H, Ar), 7.58 (d, *J* = 9.0 Hz, 2H, Ar), 7.01 (d, *J* = 9.0 Hz, 2H, Ar), 5.48 (s, 2H, CH_2_-triazole), 4.68 (m, 1H, CH), 1.30 (d, *J* = 6.0 Hz, 6H, 2 × CH_3_). ^13^C NMR (DMSO-d_6_): *δ* 158.10 (C, Ar), 152.02 (CH, triazole), 151.16 (C, pyrazole), 144.40 (CH, triazole), 138.58 (C, Ar), 130.87 (C, Ar), 130.30 (CH, pyrazole), 129.96 (2 × CH, Ar), 129.44 (2 × CH, Ar), 124.61 (C, pyrazole), 120.29 (2 × CH, Ar), 116.10 (2 × CH, Ar), 116.06 (C, Ar), 69.67 (CH), 40.81 (CH_2_), 22.29 (2 × CH_3_). HPLC: 97.0%, RT = 4.88 min. HRMS (ESI) *m*/*z*: calculated: 394.1435 [M + H]^+^, found: 394.1443 [M + H]^+^.

### Mtb MIC assay

Spot culture growth inhibition (SPOTi) assay was performed as previously described.^13^ Briefly, each well of a flat 96-well plate was spotted with 2 μL compound, serially diluted two-fold in DMSO from 150 μg mL^−1^. 200 μL Middlebrook 7H10 agar supplemented with 0.5% (v/v) glycerol and 10% OADC was dispensed into each well of a flat 96-well plate and the plate shaken to homogenise the compound. The agar was allowed to set before storage at 4 °C and used in the SPOTi assay within 24 h. To inoculate the plate, *M. tuberculosis* H37Rv was first cultured with shaking at 37 °C in Middlebrook 7H9 media supplemented with 0.5% (v/v) glycerol, 10% OADC and 0.025% Tween 80 up to an OD of 1. The culture was then diluted 1 : 100 in 7H9 media and 2 μL spotted onto each well of the SPOTi plate. Cultures were incubated at 37 °C for five weeks before MIC_90_ was recorded for each compound.

### CYP121A1 binding affinity assay

Recombinant CYP121A1 protein that was used in spectral binding assays was expressed and purified as described previously.^24^ Compounds were first screened for binding on a dual-beam Shimadzu 2700 spectrophotometer, in which ligand-free spectra of 1 μM CYP121A1 (50 mM Tris HCl, 300 mM NaCl, pH 7.4) were compared with spectra in the presence of 250 μM of each compound following a 15 min incubation period in a 1 cm quartz cuvette. The shape and intensity of preliminary difference spectra indicated peak maxima between 420–435 nm and minima between 380–415 nm and were consistent with a type-II ligand response.

Full titrations were performed in triplicate at ambient temperature using an Agilent BioTek Cytation 5 monochromator-based multimode plate reader. Protein samples at 1 μM concentration and in 200 μL volume were prepared in Corning® 96-well black flat bottom polystyrene 96 well plates. Individual wells were used to acquire 12 intermediate concentration points spanning 0 to 150 μM of compound. *K*_d_ values were calculated by plotting the inhibitor concentrations against the blue-shifted type-II response. Data fitting was performed as described previously^24^ using a single binding mode equation for hyperbolic fitting in Prism GraphPad v7.05. As additional controls, cYY substrate was included in the plate reader assay and all compounds were validated with a single manual titration on the Shimadzu 2700 spectrophotometer.

### Protein expression, purification, and sample preparation for NMR

The mutant version of CYP121A1 protein containing C171S was expressed, purified, and labelled for NMR detection as described previously.^18^ Following purification, the protein was ^19^F labelled using 3-bromo-1,1,1-trifluoroacetone (BTFA). Briefly, the protein was diluted to 2 μM in 50 mM Tris HCl, pH 7.4, and 300 mM NaCl supplemented with 10 mM BTFA and 5 mM DTT and incubated overnight at 4 °C. Unreacted BTFA and DTT were removed by size exclusion chromatography. The protein was exchanged into the NMR buffer (50 mM potassium phosphate, pH 7.4, 50 mM NaCl, and 10% D_2_O) using a 10 kDa molecular weight cut-off filter. Protein was aliquoted into separate NMR samples at 100 μM and 450 μL volume. Compounds were added from a DMSO stock to a final concentration of 300 μM and allowed to incubate for 15 min prior to transfer to NMR sample tubes. Spectra were acquired at 25 °C on a Bruker AVANCE III 600 MHz spectrometer for 10 000 scans per experiment. The data were processed and analyzed in TopSpin, version 4.1.1.

### Computational studies

Molecular docking and molecular dynamics simulations were performed as previously described.^25,26^ Briefly docking studies, using the crystal structure of Mtb CYP121A1 co-crystallised with cYY (pdb 3G5H^4^) were performed to generate PDB Mtb CYP121A1–ligand complexes, using MOE^22^ until a RMSD gradient of 0.01 kcal mol^−1^ Å^−1^ with the MMFF94 forcefield (ligands) and partial charges were automatically calculated. The charge of the haem iron at physiological pH was set to 3^+^ (geometry d^2^sp^3^) through the atom manager in MOE. Docking was performed using the Alpha Triangle placement to determine the poses, refinement of the results was done using the MMFF94 forcefield, and rescoring of the refined results using the London Δ*G* scoring function was applied. Molecular dynamics simulations were run on the Mtb CYP121A–ligand complexes as previously described^26^ using the Desmond programme of Schödinger.^23^ Overlapping water molecules were deleted, and the systems were neutralised with Na^+^ ions and salt concentration 0.15 M. Force-field parameters for the complexes were assigned using the OPLS_2005 forcefield, that is, a 150 ns molecular dynamic run in the NPT ensemble (*T* 1/4 300 K) at a constant pressure of 1 bar. Energy and trajectory atomic coordinate data were recorded at each 1.2 ns.

## Author contributions

Research concept designed by CS, DFE, SJW and SB. LAA performed the chemistry and analysis of all compounds supervised by CS. AK developed the methodology and performed the Mtb CYP121A1 and ^19^F NMR binding affinity studies supervised by DFE. SJW performed the SPOTi assay, with support and training from GS and SB. Computational studies and visualisation performed by CS. Manuscript reviewed by all authors.

## Conflicts of interest

There are no conflicts to declare.

## Supplementary Material

MD-013-D2MD00155A-s001
