# Supplementary material for: Synthesis, biological evaluation and computational studies of pyrazole derivatives as Mycobacterium tuberculosis CYP121A1 inhibitors
Source: RSC Med Chem. 2022 Aug 16;13(11):1350–60. doi: 10.1039/d2md00155a (PMC9667784; doi:10.1039/d2md00155a)
Supplement: MD-013-D2MD00155A-s001 [file MD-013-D2MD00155A-s001.pdf]

Electronic Supplementary Material (ESI) for RSC Medicinal Chemistry.
This journal is © The Royal Society of Chemistry 2022

SUPPORTING INFORMATION

Synthesis, biological evaluation and computational studies of pyrazole derivatives as *Mycobacterium tuberculosis* CYP121A1 inhibitors

Lama A. Alshabani,[a] Amit Kumar,[b] Sam J. Willcocks,[c,d] Gayathri Srithiran,[d] Sanjib Bhakta,[d] D. Fernando Estrada[b] and Claire Simons *[a]

Contents

Chemical synthesis and analysis of hydrazines **7**	**S2**
Chemical synthesis and analysis of aldehydes **8**	**S2-S4**
Chemical synthesis and analysis of alcohols **9**	**S4-S5**
Chemical synthesis and analysis of chlorides **10**	**S5-S6**
HPLC-MS for triazole derivative **12h**	**S7**
NMR (^{1}H, ^{13}C and ^{19}F) for compounds **11** and **12**	**S8-S27**
**Figure S1**. Binding difference spectra of pyrazole derivatives **11a, 11c-11h** and **12b**	**S28**
**Figure S2**. ^{19}F NMR spectra of BTFA-labelled CYP121_S171C (blue) alone, with cYY bound (red) and with exemplar pyrazole ligands (green)	**S28**
**Figure S3**. Ligand-protein complex stability through protein-ligand RMSD over 150 ns MD simulation for compounds **11**. Ligand RMSD in red and protein RMSD in blue.	**S29**
**Figure S4**. Ligand-protein complex stability through protein-ligand RMSD over 150 ns MD simulation for compounds **12**. Ligand RMSD in red and protein RMSD in blue.	**S30**

General method for the synthesis of hydrazine derivatives (7). To a solution of phenylhydrazine hydrochloride (**5** or **6**) (1-2 equiv.) in dry EtOH (1 mL/mmol) was added Et_3N (2 equiv.) and the reaction stirred at room temperature for 30 min. A solution of acetophenone (**1**-**4**) (1 equiv.) in dry EtOH (1 mL/mmol) and acetic acid (0.2 mL/mmol) was then added to the reaction, which was heated at 83 ^oC for 1 h. The solvent was then evaporated under reduced pressure and ice-H_2O added to the remaining residue. The resulting precipitate was collected by filtration, dried, and used in the next reaction without further purification.

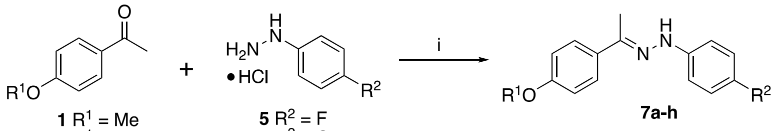

1 R^1 = Me
2 R^1 = Et
3 R^1 = Pr
4 R^1 = ^iPr

5 R^2 = F
6 R^2 = Cl

7a-h

(E)-1-(4-Fluorophenyl)-2-(1-(4-methoxyphenyl)ethylidene)hydrazine (**7a**, R^1=CH_3, R^2=F). Prepared from 4-methoxyacetophenone (**1**) (0.32 g, 2.15 mmol) and 4-fluorophenylhydrazine hydrochloride (**5**) (0.7 g, 4.30 mmol). Yield 0.55 g (100%) as an orange solid. m.p: 90 - 92° C, R_f: 0.80 (petroleum ether - EtOAc 1:2 v/v). ^1H NMR (DMSO-d_6): 9.12 (s,1H, NH), 7.72 (d, *J* = 9.0 Hz, 2H, Ar), 7.20 (m, 2H, Ar), 7.05 (m, 2H, Ar), 6.94 (d, *J* = 9.0 Hz, 2H, Ar), 3.78 (s, 3H, OCH_3), 2.22 (s, 3H, CH_3). ^{19}F NMR: (DMSO-d_6): δ -126.54 (F - Ar)

(E)-1-(4-Chlorophenyl)-2-(1-(4-methoxyphenyl)ethylidene)hydrazine (**7b**, R^1=CH_3, R^2=Cl). Prepared from 4-methoxyacetophenone (**1**) (0.75 g, 5 mmol) and 4-chlorophenylhydrazine hydrochloride (**6**) (1.8 g, 10 mmol). Yield 1.374 g (100%) as an orange solid. m.p: 112 - 114° C (lit. mp[1] 128 - 130° C), R_f: 0.89 (petroleum ether-EtOAc 1:2 v/v). ^1H NMR (DMSO-d_6): 9.27 (s,1H, NH), 7.73 (d, *J* = 9.0 Hz, 2H, Ar), 7.22 (m, 4H, Ar), 6.95 (d, *J* = 9.0 Hz, 2H, Ar), 3.07 (s, 3H, OCH_3), 2.23 (s, 3H, CH_3).

(E)-1-(1-(4-Ethoxyphenyl)ethylidene)-2-(4-fluorophenyl)hydrazine (**7c**, R^1=CH_2H_3, R^2=F). Prepared from 4-ethoxyacetophenone (**2**) (2.46 g, 15 mmol) and 4-fluorophenylhydrazine hydrochloride (**5**) (2.44 g, 15 mmol). Yield 3.85 g (94%) as a pale yellow solid. m.p: 76 - 78° C, R_f: 0.53 (petroleum ether - EtOAc 4:1 v/v). ^1H NMR (DMSO-d_6): δ 9.11 (s, 1H, NH), 7.70 (d, *J* = 8.9 Hz, 2H, Ar), 7.20 (m, 2H, Ar), 7.04 (m, 2H, Ar), 6.92 (d, *J* = 8.9 Hz, 2H, Ar), 4.05 (q, *J* = 7.0 Hz, 2H, CH_2CH_3), 2.21 (s, 3H, CH_3), 1.34 (t, *J* = 7.0 Hz, 3H, CH_2CH_3). ^{19}F NMR: (DMSO-d_6): δ -116.52 (F - Ar)

(E)-1-(4-Chlorophenyl)-2-(1-(4-ethoxyphenyl)ethylidene)hydrazine (**7d**, R^1=CH_2H_3, R^2=CL). Prepared from 4-ethoxyacetophenone (**2**) (0.821 g, 5 mmol) and 4-chlorophenylhydrazine hydrochloride (**6**) (0.895 g, 5 mmol). Yield 1.134 g (79%) as light brown solid. m.p: 133 - 136° C, R_f: 0.47 (petroleum ether - EtOAc 4:1 v/v). ^1H NMR (DMSO-d_6): 9.26 (s,1H, NH), 7.71 (d, *J* = 8.9 Hz, 2H, Ar), 7.22(m, 4H, Ar), 6.93 (d, *J* = 8.9 Hz, 2H, Ar), 4.05 (q, *J* = 7.0 Hz, 2H, CH_2CH_3), 2.22 (s, 3H, CH_3), 1.34 (t, *J* = 7.0 Hz, 3H, CH_2CH_3).

(E)-1-(4-Fluorophenyl)-2-(1-(4-propoxyphenyl)ethylidene)hydrazine (**7e**, R^1= $CH_2CH_2CH_3$, R^2=F). Variation to method – stirred at room temperature. Prepared from 4-propoxyacetophenone (**3**) (0.8 g, 4.5 mmol) and 4-fluorophenylhydrazine hydrochloride (**5**) (1.46 g, 9 mmol). Yield 1.28 g (100%) as an orange semisolid. R_f: 0.90 (petroleum ether - EtOAc 2:1 v/v). ^1H NMR (DMSO-d_6): δ 9.11 (s,1H, NH), 7.71 (d, *J* = 9.0 Hz, 2H, Ar), 7.19 (m, 2H, Ar), 7.05 (t, *J* = 9.0 Hz, 2H, Ar), 6.94 (d, *J* = 9.0 Hz, 2H, Ar), 3.94 (t, *J* = 7.0 Hz, 2H, OCH_2), 2.21 (s, 3H, CH_3), 1.75 (m, 2H, CH_3CH_2), 0.99 (d, *J* = 7.50 Hz, 3H, CH_2CH_3). ^{19}F NMR: (DMSO-d_6): δ -126.56 (F-Ar)

(E)-1-(4-Chlorophenyl)-2-(1-(4-propoxyphenyl)ethylidene)hydrazine (**7f**, R^1=$CH_2CH_2CH_3$, R^2=Cl). Prepared from 4-propoxyacetophenone (**3**) (0.8 g, 4.5 mmol) and 4-fluorophenylhydrazine hydrochloride (**6**) (1.60 g, 9 mmol). Yield 1.36 g (100%) as a light brown solid. m.p: 130 - 132° C, R_f: 0.90 (petroleum ether - EtOAc 2:1 v/v). ^1H NMR (DMSO-d_6): δ 9.27 (s, 1H, NH), 7.72 (d, *J* = 9.0 Hz, 2H, Ar), 7.23 (m, 4H, Ar), 6.94 (d, *J* = 8.50 Hz, 2H, Ar), 3.95 (t, *J* = 6.50 Hz, 2H, OCH_2), 2.22 (s, 3H, CH_3), 1.75 (m, 2H, CH_3CH_2), 0.99 (d, *J* = 7.50 Hz, 3H, CH_2CH_3).

(E)-1-(4-Fluorophenyl)-2-(1-(4-isopropoxyphenyl)ethylidene)hydrazine (**7g**, R^1=$CH(CH_3)_2$, R^2=F). Variation to method – stirred at room temperature. Prepared from 4-isopropoxyacetophenone (**4**) (0.3 g, 1.7 mmol) and 4-fluorophenylhydrazine hydrochloride (**5**) (0.54 g, 3.3 mmol). Yield 1.10 g (98%) as an orange semisolid. R_f: 0.72 (petroleum ether - EtOAc 2:1 v/v). ^1H NMR (DMSO-d_6): δ 9.11 (s, 1H, NH), 7.70 (d, *J* = 9.0 Hz, 2H, Ar), 7.19 (m, 2H, Ar), 7.04 (t, *J* = 9.0 Hz, 2H, Ar), 6.92 (d, *J* = 9.0 Hz, 2H, Ar), 4.63 (m, 1H, CH), 2.21 (s, 3H, CH_3), 1.28 (d, *J* = 6.0 Hz, 6H, 2 x CH_3).

(E)-1-(4-Chlorophenyl)-2-(1-(4-isopropoxyphenyl)ethylidene)hydrazine (**7h**, R^1=$CH(CH_3)_2$, R^2=Cl). Prepared from 4-isopropoxyacetophenone (**4**) (0.7 g, 3.9 mmol) and 4-chlorophenylhydrazine hydrochloride (**6**) (1.4 g, 7.8 mmol). Yield 1.18 g (100%) as a yellow solid. m.p: 84 - 86° C, R_f: 0.70 (petroleum ether - EtOAc 2:1 v/v). ^1H NMR (DMSO-d_6): δ 9.26 (s, 1H, NH), 7.71 (d, *J* = 8.9 Hz, 2H, Ar), 7.22 (m, 4H, Ar), 6.93 (d, *J* = 8.9 Hz, 2H, Ar), 4.64 (m, 1H, CH), 2.22 (s, 3H, CH_3), 1.28 (d, *J* = 6.0 Hz, 6H, 2 x CH_3).

General method for the synthesis of aldehyde derivatives (8). To a mixture of dry DMF (3 equiv.) and $POCl_3$ (3 equiv.) was cooled at 0° C for 5 min, then stirred at room temperature where a solution of hydrazine (**7**) (1 equiv.) in dry DMF (0.5

mL/mmol) was added dropwise to the Vilsmeier-Haack reagent, allowed to warm to room temperature, and then heated at 60° C for 3 h. The reaction was brought to pH 8.0 with cold saturated K_2CO_3 solution and the precipitate collected by filtration, washed with water, dried, and used immediately in subsequent reactions without further purification.

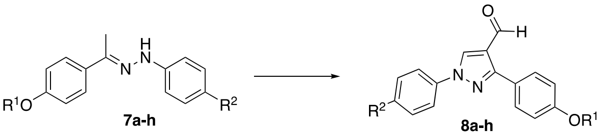

1-(4-Fluorophenyl)-3-(4-methoxyphenyl)-1H-pyrazole-4-carbaldehyde (**8a** R^1=CH_3, R^2=F). Prepared from (*E*)-1-(4-fluorophenyl)-2-(1-(4-methoxyphenyl)ethylidene)hydrazine (**7a**) (0.56 g, 2.17 mmol). Yield 0.61 g (95%) as an off-white solid. m.p: 144 - 146° C, R_f: 0.45 (petroleum ether - EtOAc 4:1 v/v). ^{1}H NMR (DMSO-d_6): δ 9.97 (s, 1H, CHO), 9.28 (s, 1H, pyrazole), 8.03 (m, 2H, Ar), 7.90 (d, *J* = 8.5 Hz, 2H, Ar), 7.43 (m, 2H, Ar), 7.07 (d, *J* = 9.0 Hz, 2H, Ar), 3.83 (s, 3H, OCH_3). ^{19}F NMR: (DMSO-d_6): δ -114.66 (F - Ar). ^{13}C NMR (DMSO-d_6): δ 185.07 (C, aldehyde), 162.49 (C, Ar), 160.55 and 162.49 (d, $^1J_{CF}$ = 244.0 Hz, C-F, Ar), 160.54 (C, Ar), 152.97 (C, pyrazole), 135.68 (d, $^4J_{CF}$ = 2.5 Hz, C, Ar), 135.57 (CH, pyrazole), 130.53 (2 x CH, Ar), 124.01 (C, Ar), 122.44 (C, pyrazole), 121.84 and 121.91 (d, $^3J_{CF}$ = 8.8 Hz, 2 x CH, Ar), 116.87 and 117.05 (d, $^2J_{CF}$ = 22.6 Hz, 2 x CH, Ar), 114.46 (2 x CH, Ar), 55.73 (CH_3).

1-(4-Chlorophenyl)-3-(4-methoxyphenyl)-1H-pyrazole-4-carbaldehyde (**8b** R^1=CH_3, R^2=Cl). Prepared from (*E*)-1-(4-chlorophenyl)-2-(1-(4-methoxyphenyl)ethylidene)hydrazine (**7b**) (1.3 g, 4.7 mmol). Yield 0.959 g (65%) as a buff solid. m.p: 142 - 144° C (lit. mp[1] 142-143[1]), R_f: 0.52 (petroleum ether - EtOAc 4:1 v/v). ^{1}H NMR (DMSO-d_6): δ 9.97 (s, 1H, CHO), 9.33 (s, 1H, pyrazole), 8.03 (d, *J* = 9.0 Hz, 2H, Ar), 7.90 (d, *J* = 9.0 Hz, 2H, Ar), 7.64 (d, *J* = 9.0 Hz, 2H, Ar), 7.07 (d, *J* = 9.0 Hz, 2H, Ar), 3.83 (s, 3H, OCH_3). ^{13}C NMR (DMSO-d_6): δ 185.07 (C, aldehyde), 160.60 (C-O, Ar), 153.07 (C, pyrazole), 137.92 (C, Ar), 135.68 (CH, pyrazole), 132.27 (C-Cl, Ar), 130.55 (2 x CH, Ar), 130.11 (2 x CH, Ar), 123.92 (C, pyrazole), 122.62 (C, Ar), 121.30 (2 x CH, Ar), 114.46 (2 x CH, Ar), 55.73 (CH_3).

3-(4-Ethoxyphenyl)-1-(4-fluorophenyl)-1H-pyrazole-4-carbaldehyde (**8c** R^1=CH_2CH_3, R^2=F). Prepared from (*E*)-1-(1-(4-ethoxyphenyl)ethylidene)-2-(4-fluorophenyl)hydrazine (**7c**) (3.0 g, 11 mmol). Yield 3.065 g (90%) as a white solid. m.p: 162 - 164° C, R_f: 0.39 (petroleum ether - EtOAc 4:1 v/v). ^{1}H NMR (DMSO-d_6): δ 9.97 (s, 1H, CHO), 9.28 (s, 1H, pyrazole), 8.03 (m, 2H, Ar), 7.89 (d, *J* = 9.0 Hz, 2H, Ar), 7.43 (m, 2H, Ar), 7.05 (d, *J* = 9.0 Hz, 2H, Ar), 4.10 (q, *J* = 7.0 Hz, 2H, CH_2CH_3), 1.37 (t, *J* = 7.0 Hz, 3H, CH_2CH_3). ^{19}F NMR: (DMSO-d_6): δ -114.68 (F - Ar). ^{13}C NMR (DMSO-d_6): δ 185.07 (C, aldehyde), 160.55 and 162.49 (d, $^1J_{CF}$ = 244.0 Hz, C-F, Ar), 159.84 (C, Ar), 152.99 (C, pyrazole), 135.69 (d, $^4J_{CF}$ = 2.5 Hz, C, Ar), 135.58 (CH, pyrazole), 130.53 (2 x CH, Ar), 123.87 (C, Ar), 122.43 (C, pyrazole), 121.83 and 121.89 (d, $^3J_{CF}$ = 7.5 Hz, 2x CH, Ar), 116.87 and 117.05 (d, $^2J_{CF}$ = 22.6 Hz, 2 x CH, Ar), 114.88 (2 x CH, Ar), 63.65 (CH_2), 15.10 (CH_3).

1-(4-Chlorophenyl)-3-(4-ethoxyphenyl)-1H-pyrazole-4-carbaldehyde (**8d** R^1=CH_2CH_3, R^2=Cl). Prepared from (*E*)-1-(4-chlorophenyl)-2-(1-(4-ethoxyphenyl)ethylidene)hydrazine (**7d**) (0.5 g, 1.7 mmol). Yield 0.525 g (93%) as a white solid. m.p: 138 - 140° C, R_f: 0.39 (petroleum ether - EtOAc 4:1 v/v). ^{1}H NMR (DMSO-d_6): δ 9.97 (s, 1H, CHO), 9.32 (s, 1H, pyrazole), 8.01 (d, *J* =9.0 Hz, 2H, Ar), 7.89 (d, *J* = 9.0 Hz, 2H, Ar), 7.64 (d, *J* = 9.0 Hz, 2H, Ar), 7.05 (d, *J* = 9.0 Hz, 2H, Ar), 4.10 (q, *J* = 7.0 Hz, 2H, CH_2CH_3), 1.37 (t, *J* = 7.0 Hz, 3H, CH_2CH_3).

1-(4-Fluorophenyl)-3-(4-propoxyphenyl)-1H-pyrazole-4-carbaldehyde (**8e** R^1=$CH_2CH_2CH_3$, R^2=F). Prepared from (*E*)-1-(4-fluorophenyl)-2-(1-(4-propoxyphenyl)ethylidene)hydrazine (**7e**) (1.2 g, 4.2 mmol). Yield 1.31 g (96%) as a yellow solid. m.p: 140 - 142° C, R_f: 0.41 (petroleum ether - EtOAc 4:1 v/v). ^{1}H NMR (DMSO-d_6): δ 9.97 (s, 1H, CHO), 9.27 (s, 1H, pyrazole), 8.04 (m, 2H, Ar), 7.90 (d, *J* = 9.0 Hz, 2H, Ar), 7.43 (t, *J* = 8.50 Hz, 2H, Ar), 7.07 (d, *J* =9.0 Hz, 2H, Ar), 4.0 (t, *J* = 6.5 Hz, 2H, OCH_2), 1.77 (m, 2H, CH_3CH_2), 1.01 (d, *J* = 7.50 Hz, 3H, CH_3). ^{19}F NMR: (DMSO-d_6): δ -114.67 (F-Ar). ^{13}C NMR (DMSO-d_6): δ 185.07 (C, aldehyde), 162.49 and 160.54 (d, $^1J_{CF}$ = 245.3 Hz, C-F, Ar), 160.0 (C, Ar), 152.99 (C, pyrazole), 135.69 (d, $^4J_{CF}$ = 2.5 Hz, C, Ar), 135.56 (CH, pyrazole), 130.52 (2 x CH, Ar), 123.86 (C, pyrazole), 122.43 (C, Ar), 121.89 and 121.82 (d, $^3J_{CF}$ = 8.80 Hz, 2 x CH, Ar), 117.05 and 116.86 (d, $^2J_{CF}$ = 23.90 Hz, 2 x CH, Ar), 114.92 (2 x CH, Ar), 69.52 (OCH_2), 22.48 (CH_2CH_3), 10.87 (CH_3).

1-(4-Chlorophenyl)-3-(4-propoxyphenyl)-1H-pyrazole-4-carbaldehyde (**8f** R^1=$CH_2CH_2CH_3$, R^2=Cl). Prepared from (*E*)-1-(4-chlorophenyl)-2-(1-(4-propoxyphenyl)ethylidene)hydrazine (**7f**) (1.3 g, 4.3 mmol). Yield 1.47 g (100%) as a yellow solid. m.p: 132 - 134° C, R_f: 0.46 (petroleum ether - EtOAc 4:1 v/v). ^{1}H NMR (DMSO-d_6): δ 9.97 (s, 1H, CHO), 9.33 (s, 1H, pyrazole), 8.03 (d, *J* = 9.0 Hz, 2H, Ar), 7.90 (d, *J* = 9.0 Hz, 2H, Ar), 7.65 (d, *J* = 9.0 Hz, 2H, Ar), 7.06 (d, *J* = 9.0 Hz, 2H, Ar), 4.0 (t, *J* = 6.50 Hz, 2H, OCH_2), 1.77 (m, 2H, CH_3CH_2), 1.0 (d, *J* = 7.50 Hz, 3H, CH_3). ^{13}C NMR (DMSO-d_6): δ 185.06 (C, aldehyde), 160.05 (C-O, Ar), 153.08 (C, pyrazole), 137.92 (C, Ar), 135.69 (CH, pyrazole), 132.26 (C-Cl, Ar), 130.53 (2 x CH, Ar), 130.10 (2 x CH, Ar), 123.77 (C, pyrazole), 122.61 (C, Ar), 121.29 (2 x CH, Ar), 114.92 (2 x CH, Ar), 69.53 (OCH_2), 22.48 (CH_2CH_3), 10.86 (CH_3).

1-(4-Fluorophenyl)-3-(4-isopropoxyphenyl)-1H-pyrazole-4-carbaldehyde (**8g**, R^1=$CH(CH_3)_2$, R^2=F). Variation from method – reaction stirred at room temperature overnight. Prepared from (*E*)-1-(4-fluorophenyl)-2-(1-(4-isopropoxyphenyl)ethylidene)hydrazine (**7g**) (1.5 g, 5.2 mmol). Yield 1.65 g (98%) as a yellow solid. m.p: 137 - 139° C, R_f: 0.40 (petroleum ether - EtOAc 4:1 v/v). ^{1}H NMR (DMSO-d_6): δ 9.97 (s, 1H, CHO), 9.27 (s, 1H, pyrazole), 8.04 (m, 2H, Ar), 7.88 (d, *J*

= 9.0 Hz, 2H, Ar), 7.42 (t, *J* = 9.0 Hz, 2H, Ar), 7.05 (d, *J* =8.50 Hz, 2H, Ar), 4.71 (m, 1H, CH), 1.31 (d, *J* = 6.0 Hz, 6H, 2 x CH_3). ^{19}F NMR: (DMSO-d_6): δ -114.68 (F-Ar). ^{13}C NMR (DMSO-d_6): δ 185.08 (C, aldehyde), 162.49 and 160.54 (d, $^1J_{CF}$ = 245.3 Hz, C-F, Ar), 158.81 (C, Ar), 153.01 (C, pyrazole), 135.70 (d, $^4J_{CF}$ = 2.5 Hz, C, Ar), 135.54 (CH, pyrazole), 130.57 (2 x CH, Ar), 123.69 (C, pyrazole), 122.42 (C, Ar), 121.88 and 121.82 (d, $^3J_{CF}$ = 7.55 Hz, 2 x CH, Ar), 117.05 and 116.87 (d, $^2J_{CF}$ = 22.6 Hz, 2 x CH, Ar), 115.91 (2 x CH, Ar), 69.73 (CH), 22.28 (2 x CH_3).

1-(4-Chlorophenyl)-3-(4-isopropoxyphenyl)-1H-pyrazole-4-carbaldehyde (**8h**, R^1=$CH(CH_3)_2$, R^2=Cl). Prepared from (*E*)-1-(4-chlorophenyl)-2-(1-(4-isopropoxyphenyl)ethylidene)hydrazine (**7h**) (1.18 g, 3.8 mmol). Yield 1.14 g (88%) as a yellow solid. m.p: 144 - 146° C, R_f: 0.52 (petroleum ether - EtOAc 4:1 v/v). ^{1}H NMR (DMSO-d_6): δ 9.97 (s, 1H, CHO), 9.32 (s, 1H, pyrazole), 8.03 (d, *J* = 9.0 Hz, 2H, Ar), 7.89 (d, *J* = 9.0 Hz, 2H, Ar), 7.65 (d, *J* = 9.0 Hz, 2H, Ar), 7.05 (d, *J* = 9.0 Hz, 2H, Ar), 4.71 (m, 1H, CH), 1.32 (d, *J* = 6.0 Hz, 6H, 2 x CH_3). ^{13}C NMR (DMSO-d_6): δ 185.08 (C, aldehyde), 158.85 (C-O, Ar), 153.10 (C, pyrazole), 137.93 (C, Ar), 135.67 (CH, pyrazole), 132.26 (C-Cl, Ar), 130.58 (2 x CH, Ar), 130.11 (2 x CH, Ar), 123.59 (C, pyrazole), 122.60 (C, Ar), 121.29 (2 x CH, Ar), 115.91 (2 x CH, Ar), 69.74 (CH), 22.29 (2x CH_3).

General method for the synthesis of alcohol derivatives (9). To an ice-cooled solution of aldehyde (**8**) (1 equiv.) in dry EtOH (3 mL/mmol) was added $NaBH_4$ (1 equiv.) in portions, and then the reaction was stirred at room temperature for 1 h. The solvent was evaporated and H_2O (7 mL/mmol) was added slowly and the reaction stirred for 30 min [2]. The reaction mixture was extracted with EtOAc (2 x 10 mL/mmol), then the combined organic layers washed with H_2O (3 x 10 mL/mmol), dried ($MgSO_4$) and evaporated under reduced pressure to give the crude alcohol (**9**).

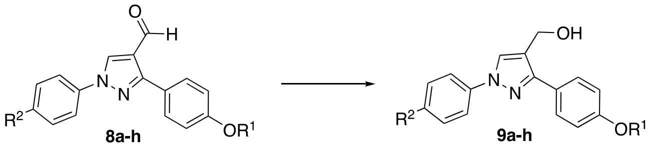

(1-(4-Fluorophenyl)-3-(4-methoxyphenyl)-1H-pyrazol-4-yl)methanol (**9a** R^1=CH_3, R^2=F). Prepared from 1-(4-fluorophenyl)-3-(4-methoxyphenyl)-1*H*-pyrazole-4-carbaldehyde (**8a**) (0.6 g, 2 mmol). Yield 0.537 g (90%) as a light orange solid. m.p: 142 - 144° C, R_f: 0.08 (petroleum ether - EtOAc 4:1 v/v). ^{1}H NMR (DMSO-d_6): δ 8.45 (s, 1H, pyrazole), 7.90 (m, 2H, Ar), 7.82 (d, *J* = 9.0 Hz, 2H, Ar), 7.35 (m, 2H, Ar), 7.02 (d, *J* = 9.0 Hz, 2H, Ar), 5.19 (t, *J* = 5.0 Hz, 1H, OH), 4.52 (d, *J* = 5.0 Hz, 2H, CH_2OH), 3.81 (s, 3H, OCH_3). ^{19}F NMR: (DMSO-d_6): δ -117.13 (F - Ar). ^{13}C NMR (DMSO-d_6): δ 159.56 and 161.49 (d, $^1J_{CF}$ = 242.7 Hz, C-F, Ar), 159.58 (C, Ar), 150.78 (C, pyrazole), 135.67 (d, $^4J_{CF}$ = 2.5 Hz, C, Ar), 129.24 (CH, pyrazole), 129.11 (2 x CH, Ar), 125.88 (C, Ar), 122.02 (C, pyrazole), 120.34 and 120.41 (d, $^3J_{CF}$ = 8.8 Hz, 2 x CH, Ar), 116.62 and 116.80 (d, $^2J_{CF}$ = 22.6 Hz, 2 x CH, Ar), 114.46 (2 x CH, Ar), 55.61 (OCH_3), 54.57 (CH_2OH).

(1-(4-Chlorophenyl)-3-(4-methoxyphenyl)-1H-pyrazol-4-yl)methanol (**9b** R^1=CH_3, R^2=Cl). Prepared from 1-(4-chlorophenyl)-3-(4-methoxyphenyl)-1*H*-pyrazole-4-carbaldehyde (**8b**) (0.95 g, 3 mmol). Yield 0.88 g (93%) as an orange solid. m.p: 112 - 114° C, R_f: 0.06 (petroleum ether - EtOAc 4:1 v/v). ^{1}H NMR (DMSO-d_6): δ 8.51 (s, 1H, pyrazole), 7.93 (d, *J* = 9.0 Hz, 2H, Ar), 7.83 (d, *J* =9.0 Hz, 2H, Ar), 7.57 (d, *J* = 9.0 Hz, 2H, Ar), 7.05 (d, *J* = 9.0 Hz, 2H, Ar), 5.20 (t, *J* = 5.0 Hz, 1H, OH), 4.53 (d, *J* = 5.0 Hz, 2H, CH_2OH), 3.81 (s, 3H, OCH_3). ^{13}C NMR (DMSO-d_6): δ 159.66 (C, Ar), 151.05 (C, pyrazole), 138.86 (C, Ar), 130.36 (C, Ar), 129.91 (2 x CH, Ar), 129.22 (CH, pyrazole), 129.14 (2 x CH, Ar), 125.74 (C, Ar), 122.37 (C, pyrazole), 119.97 (2 x CH, Ar), 114.48 (2 x CH, Ar), 55.62 (OCH_3), 54.57 (CH_2).

(3-(4-Ethoxyphenyl)-1-(4-fluorophenyl)-1H-pyrazol-4-yl)methanol (**9c** R^1=CH_2CH_3, R^2=F). Variation to method - The crude product was purified by gradient column chromatography and the pure compound eluted with CH_2Cl_2 – MeOH 99:1 v/v. Prepared from 3-(4-ethoxyphenyl)-1-(4-fluorophenyl)-1*H*-pyrazole-4-carbaldehyde (**8c**) (2.5 g, 8 mmol). Yield 1.687 g (68%) as a light brown solid. m.p: 106 - 108° C, R_f: 0.09 (petroleum ether - EtOAc 4:1 v/v). ^{1}H NMR (DMSO-d_6): δ 8.46 (s, 1H, pyrazole), 7.91 (m, 2H, Ar), 7.81 (d, *J* = 9.0 Hz, 2H, Ar), 7.35 (m, 2H, Ar), 7.02 (d, *J* = 9.0 Hz, 2H, Ar), 5.18 (t, *J* = 5.0 Hz, 1H, OH), 4.53 (d, *J* = 5.0 Hz, 2H, CH_2OH), 4.08 (q, *J* = 6.50 Hz, 2H, CH_2CH_3), 1.36 (t, *J* = 7.0 Hz, 3H, CH_2CH_3). ^{19}F NMR: (DMSO-d_6): -117.16 (F – Ar). ^{13}C NMR (DMSO-d_6): δ 159.55 and 161.48 (d, $^1J_{CF}$ = 242.7 Hz, C-F, Ar), 158.85 (C, Ar), 150.79 (C, pyrazole), 135.67 (d, $^4J_{CF}$ = 2.5 Hz, C, Ar), 129.24 (CH, pyrazole), 129.10 (2x CH, Ar), 125.75 (C, Ar), 122.0 (C, pyrazole), 120.32 and 120.39 (d, $^3J_{CF}$ = 8.8 Hz, 2 x CH, Ar), 116.61 and 116.79 (d, $^2J_{CF}$ = 22.6 Hz, 2 x CH, Ar), 114.90 (2 x CH, Ar), 63.51 (CH_2), 54.57 (CH_2OH), 15.10 (CH_3).

(1-(4-Chlorophenyl)-3-(4-ethoxyphenyl)-1H-pyrazol-4-yl)methanol (**9d** R^1=CH_2CH_3, R^2=Cl). Prepared from 1-(4-chlorophenyl)-3-(4-ethoxyphenyl)-1*H*-pyrazole-4-carbaldehyde (**8d**) (0.327 g, 1 mmol). Yield 0.296 g (90%) as a white crystalline solid. m.p: 128 - 130° C, R_f: 0.1 (petroleum ether - EtOAc 4:1 v/v). ^{1}H NMR (DMSO-d_6): δ 8.50 (s, 1H, pyrazole), 7.92 (d, *J* = 9.0 Hz, 2H, Ar), 7.81 (d, *J* = 9.0 Hz, 2H, Ar), 7.56 (d, *J* = 9.0 Hz, 2H, Ar), 7.02 (d, *J* = 9.0 Hz, 2H, Ar), 5.20 (t, *J* = 4.95 Hz, 1H, OH), 4.51 (d, *J* = 4.9 Hz, 2H, CH_2OH), 4.08 (q, *J* = 7.0 Hz, 2H, CH_2CH_3), 1.36 (t, *J* = 7.0 Hz, 3H, CH_2CH_3). ^{13}C NMR (DMSO-

d_6): δ 158.93 (C, Ar), 151.06 (C, pyrazole), 138.86 (C, Ar), 130.35 (C, Ar), 129.91 (2 x CH, Ar), 129.22 (2 x CH, Ar), 129.14 (CH, pyrazole), 125.61 (C, Ar), 122.35 (C, pyrazole), 119.95 (2 x CH, Ar), 114.91 (2 x CH, Ar), 63.53 (CH_2), 54.57 (CH_2), 15.13 (CH_3).

(1-(4-Fluorophenyl)-3-(4-propoxyphenyl)-1H-pyrazol-4-yl)methanol (**9e** R^1=$CH_2CH_2CH_3$, R^2=F). Prepared from 1-(4-fluorophenyl)-3-(4-propoxyphenyl)-1*H*-pyrazole-4-carbaldehyde (**8e**) (0.90 g, 2.77 mmol). Yield 0.80 g (89%) as an orange solid. m.p: 110 - 112° C, R_f: 0.1 (petroleum ether - EtOAc 4:1 v/v). ^{1}H NMR (DMSO-d_6): δ 8.45 (s, 1H, pyrazole), 7.92 (m, 2H, Ar), 7.81 (d, *J* = 9.0 Hz, 2H, Ar), 7.35 (t, *J* = 9.0 Hz, 2H, Ar), 7.03 (d, *J* = 9.0 Hz, 2H, Ar), 5.18 (t, *J* = 5.0 Hz, 1H, OH), 4.53 (d, *J* = 5.0 Hz, 2H, CH_2OH), 3.98 (t, *J* = 6.5 Hz, 2H, OCH_2), 1.76 (m, 2H, CH_3CH_2), 1.0 (d, *J* = 7.5 Hz, 3H, CH_3). ^{19}F NMR: (DMSO-d_6): -117.16 (F-Ar). ^{13}C NMR (DMSO-d_6): δ 159.55 and 161.48 (d, $^1J_{CF}$ = 242.7 Hz, C-F, Ar), 159.02 (C, Ar), 150.79 (C, pyrazole), 136.68 (d, $^4J_{CF}$ = 2.5 Hz, C, Ar), 129.23 (CH, pyrazole), 129.10 (2x CH, Ar), 125.75 (C, Ar), 122.01 (C, pyrazole), 120.33 and 120.39 (d, $^3J_{CF}$ = 7.6 Hz, 2 x CH, Ar), 116.61 and 116.79 (d, $^2J_{CF}$ = 22.60 Hz, 2 x CH, Ar), 114.94 (2x CH, Ar), 69.42 (OCH_2), 54.58 (CH_2), 22.53 (CH_2CH_3), 10.89 (CH_3).

(1-(4-Chlorophenyl)-3-(4-propoxyphenyl)-1H-pyrazol-4-yl)methanol (**9f** R^1=$CH_2CH_2CH_3$, R^2=Cl). Prepared from 1-(4-chlorophenyl)-3-(4-propoxyphenyl)-1*H*-pyrazole-4-carbaldehyde (**8f**) (0.90 g, 2.77 mmol). Yield 0.64 g (80%) as a light brown solid. m.p: 100 - 102° C, R_f: 0.08 (petroleum ether - EtOAc 4:1 v/v). ^{1}H NMR (DMSO-d_6): δ 8.51 (s, 1H, pyrazole), 7.93 (d, *J* = 9.0 Hz, 2H, Ar), 7.82 (d, *J* = 9.0 Hz, 2H, Ar), 7.57 (d, *J* = 9.0 Hz, 2H, Ar), 7.03 (d, *J* = 9.0 Hz, 2H, Ar), 5.20 (t, *J* = 5.0 Hz, 1H, OH), 4.53 (d, *J* = 4.50 Hz, 2H, CH_2OH), 3.98 (t, *J* = 7.0 Hz, 2H, OCH_2), 1.77 (m, 2H, CH_3CH_2), 1.0 (d, *J* = 7.50 Hz, 3H, CH_3). ^{13}C NMR (DMSO-d_6): δ 159.10 (C, Ar), 151.06 (C, pyrazole), 138.86 (C, Ar), 130.35 (C, Ar), 129.91 (2 x CH, Ar), 129.21 (CH, pyrazole), 129.13 (2 x CH, Ar), 125.60 (C, Ar), 122.35 (C, pyrazole), 119.95 (2 x CH, Ar), 114.96 (2 x CH, Ar), 69.43 (OCH_2), 54.58 (CH_2), 22.53 (CH_2CH_3), 10.90 (CH_3).

(1-(4-Fluorophenyl)-3-(4-isopropoxyphenyl)-1H-pyrazol-4-yl)methanol (**9g** R^1=$CH(CH_3)_2$, R^2=F). Prepared from 1-(4-fluorophenyl)-3-(4-isopropoxyphenyl)-1*H*-pyrazole-4-carbaldehyde (**8g**) (0.80 g, 2.45 mmol). Yield 0.65 g (81%) as a buff solid. m.p: 110 - 112° C, R_f: 0.1 (petroleum ether - EtOAc 4:1 v/v). ^{1}H NMR (DMSO-d_6): δ 8.45 (s, 1H, pyrazole), 7.91 (m, 2H, Ar), 7.80 (d, *J* = 9.0 Hz, 2H, Ar), 7.35 (m, 2H, Ar), 7.0 (d, *J* = 9.0 Hz, 2H, Ar), 5.18 (t, *J* = 5.0 Hz, 1H, OH), 4.67 (m, 1H, CH), 4.52 (d, *J* = 4.5 Hz, 2H, CH_2OH), 1.30 (d, *J* = 6.0 Hz, 6H, 2 x CH_3). ^{19}F NMR: (DMSO-d_6): -117.16 (F – Ar). ^{13}C NMR (DMSO-d_6): δ 159.55 and 161.48 (d, $^1J_{CF}$ = 242.7 Hz, C-F, Ar), 157.79 (C, Ar), 150.81 (C, pyrazole), 136.69 (d, $^4J_{CF}$ = 2.5 Hz, C, Ar), 129.22 (CH, pyrazole), 129.15 (2 x CH, Ar), 125.60 (C, Ar), 121.99 (C, pyrazole), 120.31 and 120.38 (d, $^3J_{CF}$ = 8.8 Hz, 2 x CH, Ar), 116.61 and 116.80 (d, $^2J_{CF}$ = 23.9 Hz, 2 x CH, Ar), 116.0 (2 x CH, Ar), 69.60 (CH), 54.57 (CH_2), 22.32 (2x CH_3).

(1-(4-Chlorophenyl)-3-(4-isopropoxyphenyl)-1H-pyrazol-4-yl)methanol (**9h** R^1= $CH(CH_3)_2$, R^2=Cl). Prepared from 1-(4-chlorophenyl)-3-(4-isopropoxyphenyl)-1*H*-pyrazole-4-carbaldehyde (**8h**) (0.80 g, 2.3 mmol). Yield 0.77 g (98%) as a brown solid. m.p: 116 - 118° C, R_f: 0.09 (petroleum ether - EtOAc 4:1 v/v). ^{1}H NMR (DMSO-d_6): δ 8.51 (s, 1H, pyrazole), 7.91 (d, *J* = 9.0 Hz, 2H, Ar), 7.80 (d, *J* = 8.5 Hz, 2H, Ar), 7.56 (d, *J* = 9.0 Hz, 2H, Ar), 7.0 (d, *J* = 9.0 Hz, 2H, Ar), 5.19 (t, *J* = 5.0 Hz, 1H, OH), 4.68 (m, 1H, CH), 4.52 (d, *J* = 5.0 Hz, 2H, CH_2OH), 1.30 (d, *J* = 6.0 Hz, 6H, 2 x CH_3). ^{13}C NMR (DMSO-d_6): δ 157.87 (C, Ar), 151.08 (C, pyrazole), 138.87 (C, Ar), 130.34 (C, Ar), 129.91 (2 x CH, Ar), 129.21 (CH, pyrazole), 129.18 (2 x CH, Ar), 125.46 (C, Ar), 122.34 (C, pyrazole), 119.94 (2 x CH, Ar), 116.01 (2 x CH, Ar), 69.61 (CH), 54.57 (CH_2), 22.32 (2x CH_3).

General method for the synthesis of chloride derivatives (10). To an ice-cooled solution of alcohol (**9**) (1 equiv.) in dry CH_2Cl_2 (2 mL/mmol) was added thionyl chloride ($SOCl_2$) (10 equiv.) dropwise. The reaction was stirred at 40° C for 4 h. The solvent was evaporated under reduced pressure and then while cooling in an ice-bath, carefully quenched with saturated aqueous $NaHCO_3$ in portions until slightly basic (pH 8.0). The product was extracted with EtOAc (3 x 10 mL/mmol), and the combined organic layers washed with brine (3 x 4 mL/mmol), H_2O (2 x 4 mL/mmol), dried ($MgSO_4$) and evaporated under reduced pressure to give the crude chloride (**10**), which was used immediately in subsequent reactions without further purification.

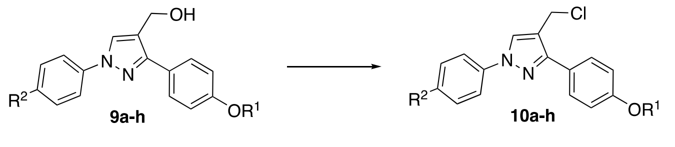

4-(Chloromethyl)-1-(4-fluorophenyl)-3-(4-methoxyphenyl)-1H-pyrazole (**10a** R^1=CH_3, R^2=F). Prepared from (1-(4-fluorophenyl)-3-(4-methoxyphenyl)-1*H*-pyrazol-4-yl)methanol carbaldehyde (**9a**) (0.50 g, 1.7 mmol). Yield 0.504 g (94%) as a brown solid. m.p: 116 - 118° C, R_f: 0.67 (petroleum ether - EtOAc 4:1 v/v). ^{1}H NMR (DMSO-d_6): δ 8.69 (s, 1H, pyrazole), 7.91 (m, 2H, Ar), 7.78 (d, *J* = 8.5 Hz, 2H, Ar), 7.38 (m, 2H, Ar), 7.08 (d, *J* = 9.0 Hz, 2H, Ar), 4.89 (s, 2H, CH_2Cl), 3.82 (s, 3H, OCH_3). ^{19}F NMR: (DMSO-d_6): -116.25 (F – Ar). ^{13}C NMR (DMSO-d_6): δ 159.89 and 161.83 (d, $^1J_{CF}$ = 244 Hz, C-F, Ar), 159.90 (C, Ar), 150.89 (C, pyrazole), 136.26 (d, $^4J_{CF}$ = 2.5 Hz, C, Ar), 130.78 (CH, pyrazole), 129.17 (2 x CH, Ar), 125.01 (C, Ar), 120.86 and 120.79 (d, $^3J_{CF}$ = 8.8 Hz, 2x CH, Ar), 117.67 (C, pyrazole), 116.91 and 116.72 (d, $^2J_{CF}$ = 23.8 Hz, 2 x CH, Ar), 114.70 (2 x CH, Ar), 55.67 (OCH_3), 38.40 (CH_2Cl).

4-(Chloromethyl)-1-(4-chlorophenyl)-3-(4-methoxyphenyl)-1H-pyrazole (**10b** $R^1=CH_3$, $R^2=Cl$). Prepared from (1-(4-chlorophenyl)-3-(4-methoxyphenyl)-1*H*-pyrazol-4-yl)methanol carbaldehyde (**9b**) (0.80 g, 2.5 mmol). Yield 0.825 g (99%) as a brown oil. R_f: 0.67 (petroleum ether - EtOAc 4:1 v/v). ^{1}H NMR (DMSO-d_6): δ 8.74 (s, 1H, pyrazole), 7.91 (d, *J* = 8.5 Hz, 2H, Ar), 7.78 (d, *J* = 9.0 Hz, 2H, Ar), 7.59 (d, *J* = 9.0 Hz, 2H, Ar), 7.08 (d, *J* = 9.0 Hz, 2H, Ar), 4.89 (s, 2H, CH_2Cl), 3.82 (s, 3H, CH_3). ^{13}C NMR (DMSO-d_6): δ 159.95 (C, Ar), 151.12 (C, pyrazole), 138.46 (C, Ar), 131.04 (C, Ar), 130.76 (CH, pyrazole), 130.0 (2 x CH, Ar), 129.20 (2 x CH, Ar), 124.88 (C, Ar), 120.34 (2 x CH, Ar), 118.0 (C, pyrazole), 114.72 (2 x CH, Ar), 55.68 (CH_3), 38.30 (CH_2Cl).

4-(Chloromethyl)-3-(4-ethoxyphenyl)-1-(4-fluorophenyl)-1H-pyrazole (**10c** $R^1=CH_2CH_3$, $R^2=F$). Prepared from (3-(4-Ethoxyphenyl)-1-(4-fluorophenyl)-1*H*-pyrazol-4-yl)methanol (**9c**) (0.30 g, 1.0 mmol). Yield 0.295 g (98%) as a brown oil. R_f: 0.64 (petroleum ether - EtOAc 4:1 v/v). ^{1}H NMR (DMSO-d_6): δ 8.69 (s, 1H, pyrazole), 7.91 (m, 2H, Ar), 7.76 (d, *J* = 9.0 Hz, 2H, Ar), 7.38 (m, 2H, Ar), 7.06 (d, *J* = 9.0 Hz, 2H, Ar), 4.89 (s, 2H, CH_2Cl), 4.09 (q, *J* = 7.0 Hz, 2H, CH_2CH_3), 1.36 (t, *J* = 7.0 Hz, 3H, CH_2CH_3). ^{19}F NMR: (DMSO-d_6): -116.27 (F – Ar). ^{13}C NMR (DMSO-d_6): δ 159.89 and 161.83 (d, $^{1}J_{CF}$ = 244.0 Hz, C-F, Ar), 159.17 (C, Ar), 150.90 (C, pyrazole), 136.27 (d, $^{4}J_{CF}$ = 2.5 Hz, C, Ar), 130.78 (CH, pyrazole), 129.16 (2 x CH, Ar), 124.87 (C, Ar), 120.85 and 120.78 (d, $^{3}J_{CF}$ = 8.8 Hz, 2 x CH, Ar), 117.65 (C, pyrazole), 116.91 and 116.73 (d, $^{2}J_{CF}$ = 22.6 Hz, 2 x CH, Ar), 115.13 (2 x CH, Ar), 63.59 (CH_2), 38.42 (CH_2Cl), 15.12 (CH_3).

4-(Chloromethyl)-1-(4-chlorophenyl)-3-(4-ethoxyphenyl)-1H-pyrazole (**10d** $R^1=CH_2CH_3$, $R^2=Cl$). Prepared from (1-(4-chlorophenyl)-3-(4-ethoxyphenyl)-1*H*-pyrazol-4-yl)methanol carbaldehyde (**9d**) (0.99 g, 3 mmol). Yield 0.97 g (93%) as a brown solid. m.p: 90 - 92° C, R_f: 0.65 (petroleum ether - EtOAc 4:1 v/v). ^{1}H NMR (DMSO-d_6): δ 8.75 (s, 1H, pyrazole), 7.92 (d, *J* = 9.0 Hz, 2H, Ar), 7.77 (d, *J* = 9.0 Hz, 2H, Ar), 7.59 (d, *J* = 9.0 Hz, 2H, Ar), 7.07 (d, *J* = 9.0 Hz, 2H, Ar), 4.89 (s, 2H, CH_2Cl), 4.10 (q, *J* = 7.0 Hz, 2H, CH_2CH_3), 1.36(t, *J* = 7.0 Hz, 3H, CH_2CH_3). ^{13}C NMR (DMSO-d_6): δ 159.24 (C, Ar), 151.12 (C, pyrazole), 138.47 (C, Ar), 131.02 (C, Ar), 130.77 (CH, pyrazole), 130.01 (2 x CH, Ar), 129.19 (2 x CH, Ar), 124.74 (C, Ar), 120.33 (2 x CH, Ar), 117.98 (C, pyrazole), 115.14 (2 x CH, Ar), 63.60 (CH_2CH_3), 38.32 (CH_2Cl), 15.12 (CH_2CH_3).

4-(Chloromethyl)-1-(4-fluorophenyl)-3-(4-propoxyphenyl)-1H-pyrazole (**10e** $R^1=CH_2CH_2CH_3$, $R^2=F$). Prepared from (1-(4-fluorophenyl)-3-(4-propoxyphenyl)-1*H*-pyrazol-4-yl)methanol (**9e**) (0.75 g, 2.3 mmol). Yield 0.79 g (100%) as a brown semisolid. R_f: 0.68 (petroleum ether - EtOAc 4:1 v/v). ^{1}H NMR (DMSO-d_6): δ 8.69 (s, 1H, pyrazole), 7.92 (m, 2H, Ar), 7.77 (d, *J* = 9.0 Hz, 2H, Ar), 7.37 (t, *J* = 9.0 Hz, 2H, Ar), 7.06 (d, *J* = 9.0 Hz, 2H, Ar), 4.89 (s, 2H, CH_2Cl), 3.99 (t, *J* = 7.0 Hz, 2H, OCH_2), 1.77 (m, 2H, CH_3CH_2), 1.0 (d, *J* = 7.50 Hz, 3H, CH_3). ^{19}F NMR: (DMSO-d_6): -116.91 (F-Ar). ^{13}C NMR (DMSO-d_6): δ 159.89 and 161.82 (d, $^{1}J_{CF}$ = 242.7 Hz, C-F, Ar), 159.33 (C, Ar), 150.90 (C, pyrazole), 136.28 (d, $^{4}J_{CF}$ = 2.5 Hz, C, Ar), 130.77 (CH, pyrazole), 129.16 (2 x CH, Ar), 124.87 (C, Ar), 120.84 and 120.77 (d, $^{3}J_{CF}$ = 8.80 Hz, 2 x CH, Ar), 117.65 (C, pyrazole), 116.90 and 116.72 (d, $^{2}J_{CF}$ = 22.64 Hz, 2 x CH, Ar), 115.17 (2 x CH, Ar), 69.48 (OCH_2), 38.41 (CH_2Cl), 22.50 (CH_2CH_3), 10.88 (CH_3).

4-(Chloromethyl)-1-(4-chlorophenyl)-3-(4-propoxyphenyl)-1H-pyrazole (**10f** $R^1=CH_2CH_2CH_3$, $R^2=Cl$). Prepared from (1-(4-chlorophenyl)-3-(4-propoxyphenyl)-1*H*-pyrazol-4-yl)methanol (**9f**) (0.64 g, 1.87 mmol). Yield 0.66 g (98%) as a brown semisolid. R_f: 0.67 (petroleum ether - EtOAc 4:1 v/v). ^{1}H NMR (DMSO-d_6): δ 8.75 (s, 1H, pyrazole), 7.90 (d, *J* = 9.0 Hz, 2H, Ar), 7.76 (d, *J* = 9.0 Hz, 2H, Ar), 7.60 (d, *J* = 9.0 Hz, 2H, Ar), 7.08 (d, *J* = 9.0 Hz, 2H, Ar), 4.89 (s, 2H, CH_2Cl), 4.0 (t, *J* = 6.50 Hz, 2H, OCH_2), 1.77 (m, 2H, CH_3CH_2), 1.0 (d, *J* = 7.50 Hz, 3H, CH_3). ^{13}C NMR (DMSO-d_6): δ 159.40 (C, Ar), 151.13 (C, pyrazole), 138.47 (C, Ar), 131.02 (C, Ar), 130.76 (CH, pyrazole), 130.0 (2 x CH, Ar), 129.18 (2 x CH, Ar), 124.74 (C, Ar), 120.33 (2 x CH, Ar), 117.98 (C, pyrazole), 115.18 (2 x CH, Ar), 69.49 (OCH_2), 38.32 (CH_2Cl), 22.50 (CH_2CH_3), 10.88 (CH_3).

4-(Chloromethyl)-1-(4-fluorophenyl)-3-(4-isopropoxyphenyl)-1H-pyrazole (**10g** $R^1= CH(CH_3)_2$, $R^2=F$). Prepared from (1-(4-fluorophenyl)-3-(4-isopropoxyphenyl)-1*H*-pyrazol-4-yl)methanol (**9g**) (0.6 g, 1.8 mmol). Yield 0.58 g (94%) as a brown oil. R_f: 0.67 (petroleum ether - EtOAc 4:1 v/v). ^{1}H NMR (DMSO-d_6): δ 8.69 (s, 1H, pyrazole), 7.91 (m, 2H, Ar), 7.76 (d, *J* = 8.5 Hz, 2H, Ar), 7.38 (t, *J* = 9.0 Hz, 2H, Ar), 7.06 (d, *J* = 8.50 Hz, 2H, Ar), 4.89 (s, 2H, CH_2Cl), 4.69 (m, 1H, CH), 1.31 (d, *J* = 6.0 Hz, 6H, 2 x CH_3). ^{19}F NMR: (DMSO-d_6): -116.95 (F-Ar). ^{13}C NMR (DMSO-d_6): δ 159.89 and 161.82 (d, $^{1}J_{CF}$ = 242.7 Hz, C-F, Ar), 158.12 (C, Ar), 150.91 (C, pyrazole), 136.28 (d, $^{4}J_{CF}$ = 2.5 Hz, C, Ar), 130.77 (CH, pyrazole), 129.20 (2 x CH, Ar), 124.70 (C, Ar), 120.82 and 120.76 (d, $^{3}J_{CF}$ = 7.6 Hz, 2 x CH, Ar), 117.64 (C, pyrazole), 116.91 and 116.72 (d, $^{2}J_{CF}$ = 23.8 Hz, 2 x CH, Ar), 116.18 (2 x CH, Ar), 69.68 (CH), 38.43 (CH_2Cl), 22.30 (2 x CH_3).

4-(Chloromethyl)-1-(4-chlorophenyl)-3-(4-isopropoxyphenyl)-1H-pyrazole (**10h** $R^1= CH(CH_3)_2$, $R^2=Cl$). Prepared from (1-(4-chlorophenyl)-3-(4-isopropoxyphenyl)-1*H*-pyrazol-4-yl)methanol (**9h**) (0.7 g, 2.0 mmol). Yield 0.68 g (94%) as a brown oil. R_f: 0.68 (petroleum ether - EtOAc 4:1 v/v). ^{1}H NMR (DMSO-d_6): δ 8.75 (s, 1H, pyrazole), 7.92 (d, *J* = 9.0 Hz, 2H, Ar), 7.76 (d, *J* = 9.0 Hz, 2H, Ar), 7.60 (d, *J* = 9.0 Hz, 2H, Ar), 7.06 (d, *J* = 8.50 Hz, 2H, Ar), 4.89 (s, 2H, CH_2Cl), 4.69 (m, 1H, CH), 1.31 (d, *J* = 6.0 Hz, 6H, 2 x CH_3). ^{13}C NMR (DMSO-d_6): δ 158.20 (C, Ar), 151.13 (C, pyrazole), 138.48 (C, Ar), 131.01 (C, Ar), 130.76 (CH, pyrazole), 130.0 (2 x CH, Ar), 129.23 (2 x CH, Ar), 124.57 (C, Ar), 120.32 (2 x CH, Ar), 117.97 (C, pyrazole), 116.19 (2 x CH, Ar), 69.69 (CH), 38.33 (CH_2Cl), 22.30 (2 x CH_3).

Reference

1. F.A. Ragab, N.M. Abdel Gawad, H.H. Georgey, and M.F. Said. *Eur. J. Med. Chem.* 2013, **63**, 645–654.

HPLC analysis

The purity of the final compound **12h** was determined by HPLC UV with correlating MS in Positive ionization mode on a Zorbax Eclipse Plus C18 Rapid Resolution 2.1 x 50 mm, 1.8 µm particle size using a 7.5-minute gradient method 5:95 v/v water: methanol with 0.1% formic acid as additive.

The HPLC data of compound **12h (**HPLC purity 97.0 %. Peak at 4.88 min**):**

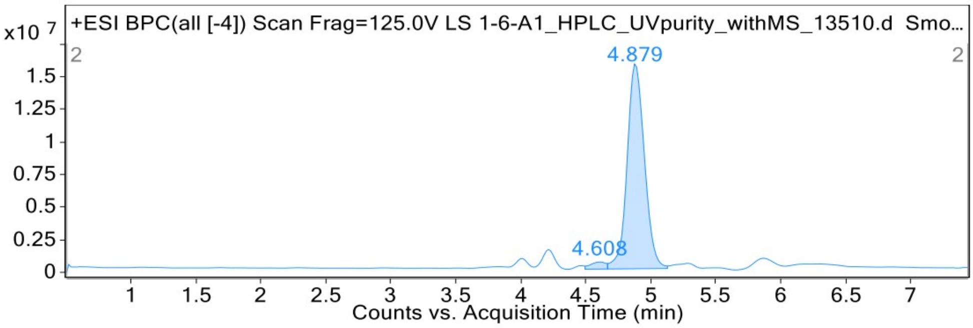

Figure 1: Base peak chromatogram

User Chromatogram Peak List

RT (min)	Area	Area %	Area Sum (%)	Base Peak (m/z)	Width (min)
4.61	4476097	3.06	2.97	388.0761	0.126
4.88	146185884	100.00	97.03	394.1378	0.148

Compound Table

Compound Label	RT (min)	Observed mass (m/z)	Neutral observed mass (Da)	Theoretical mass (Da)	Mass error (ppm)	Isotope match score (%)
Cpd 1: C21 H20 Cl N5 O	4.88	394.1443	393.1371	393.1356	3.74	92.41

Mass errors of between -5.00 and 5.00 ppm with isotope match scores above 60% are considered confirmation of molecular formulae

The ^{1}H- and ^{13}C-NMR spectra of all final compounds are shown here:

^{1}H NMR spectrum (500 MHz, DMSO-d_6) of **11a**:

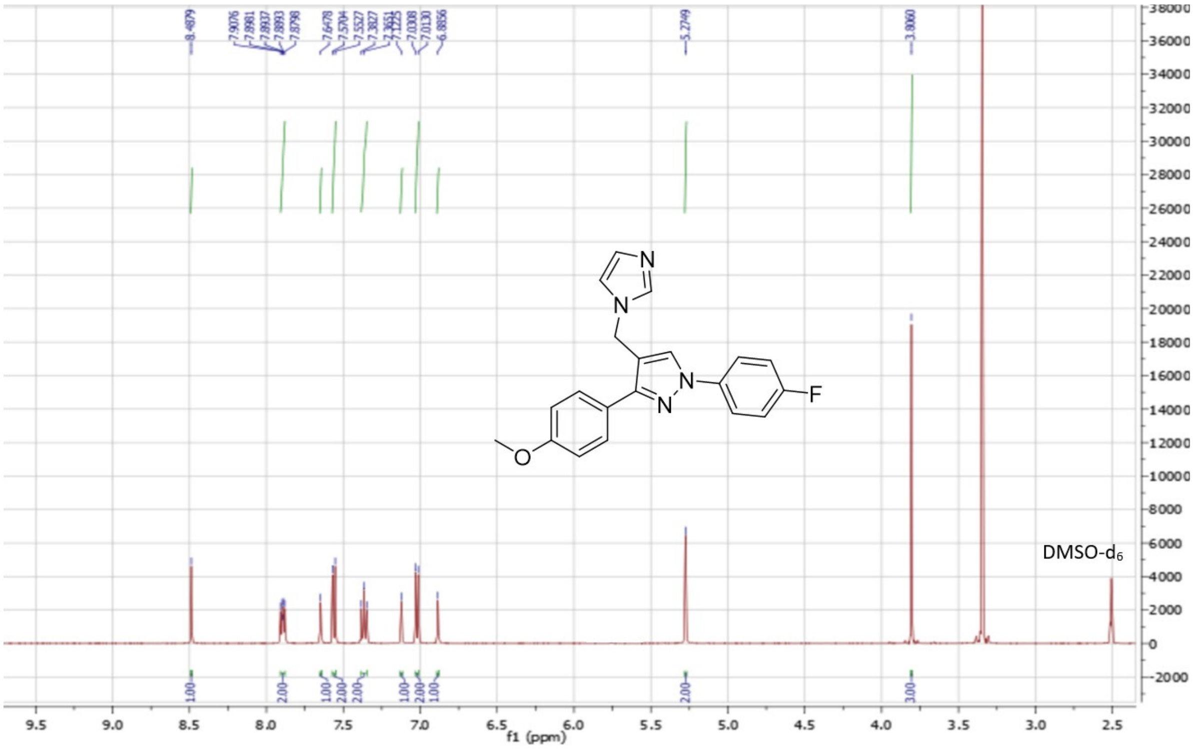

^{13}C NMR spectrum (125.75 MHz, DMSO-d_6) of **11a**:

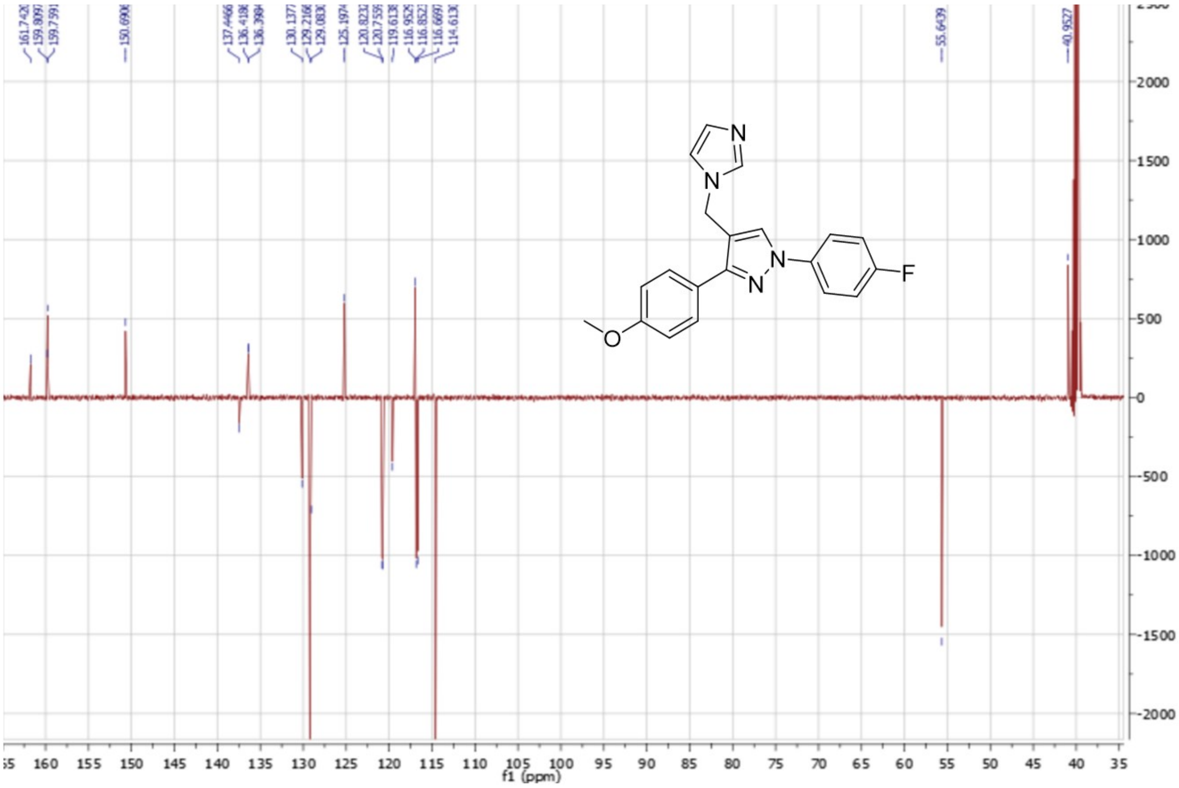

^{19}F NMR (470 MHz, DMSO-d$_6$) of **11a**:

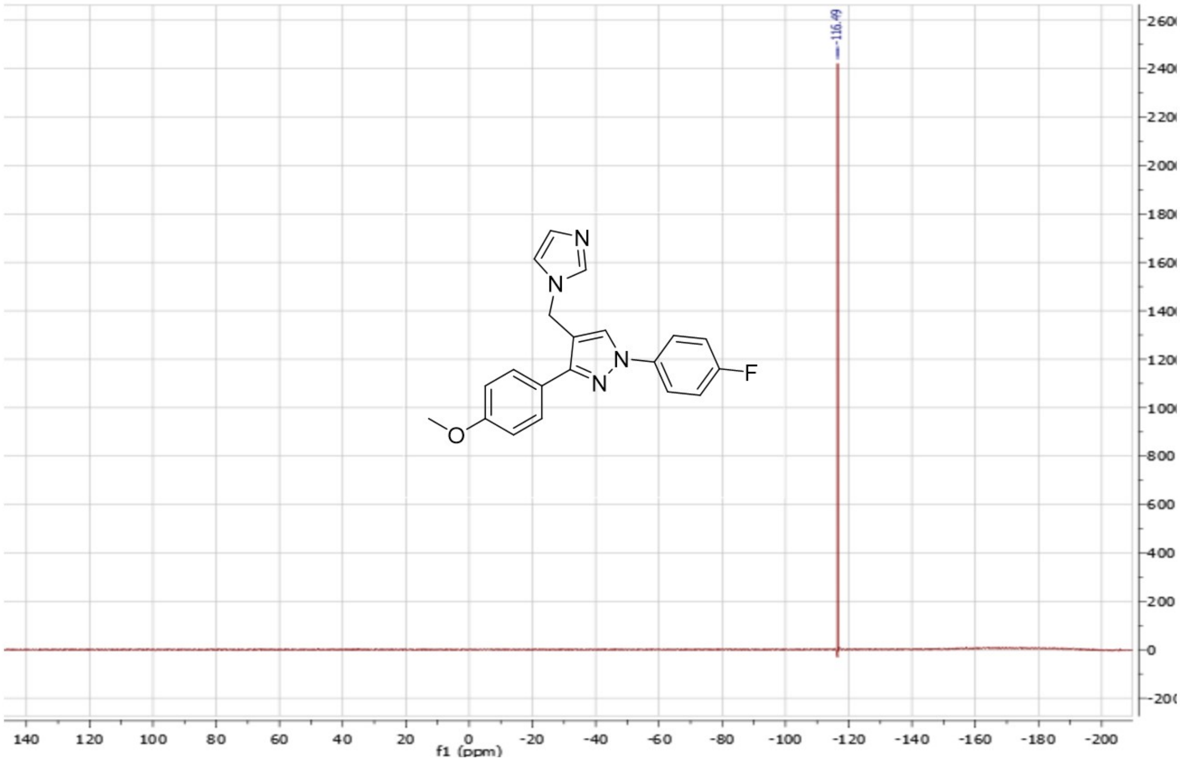

^{1}H NMR spectrum (500 MHz, DMSO-d$_6$) of **11b**:

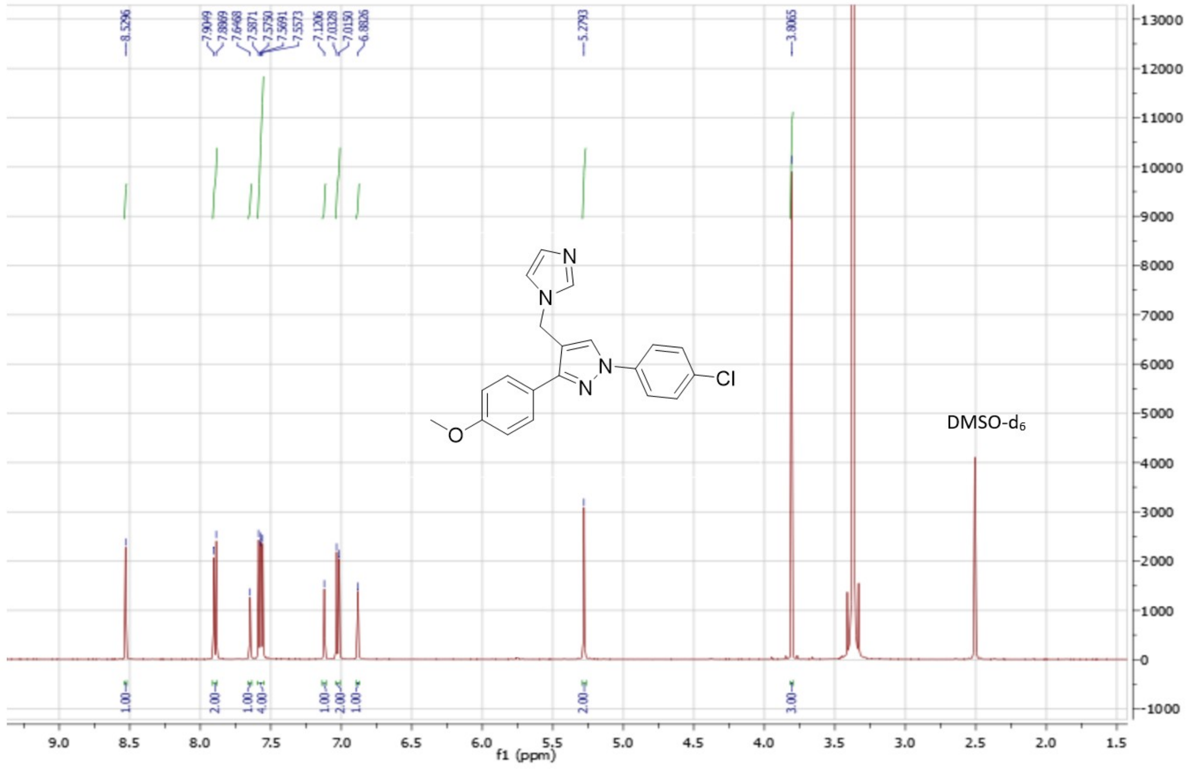

^{13}C NMR spectrum (125.75 MHz, DMSO-d_6) of **11b**:

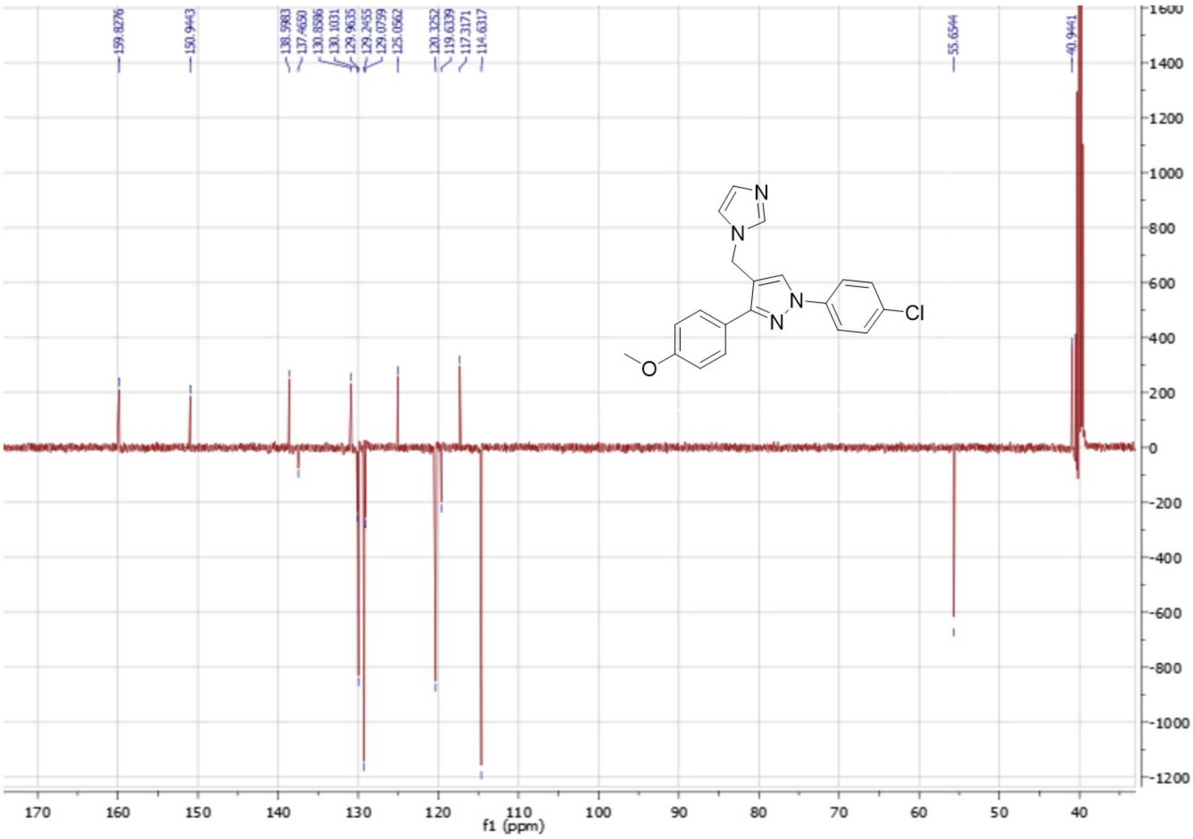

^{1}H NMR spectrum (500 MHz, DMSO-d_6) of **11c**:

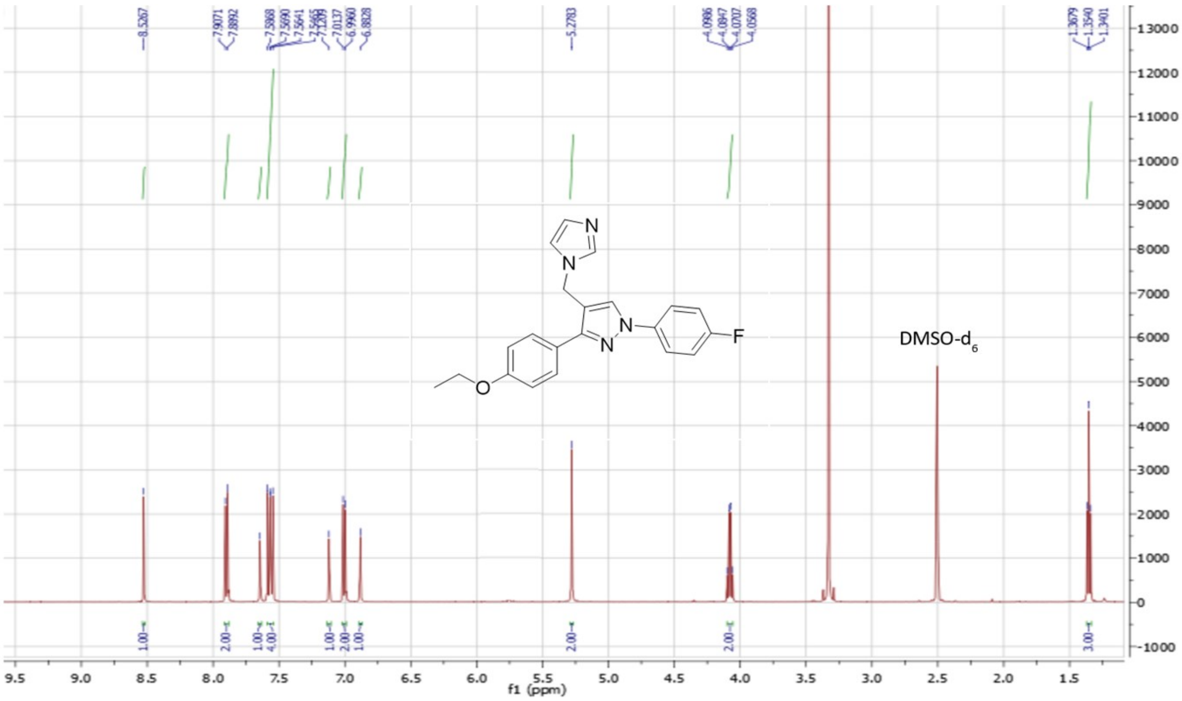

^{13}C NMR spectrum (125.75 MHz, DMSO-d$_6$) of **11c:**

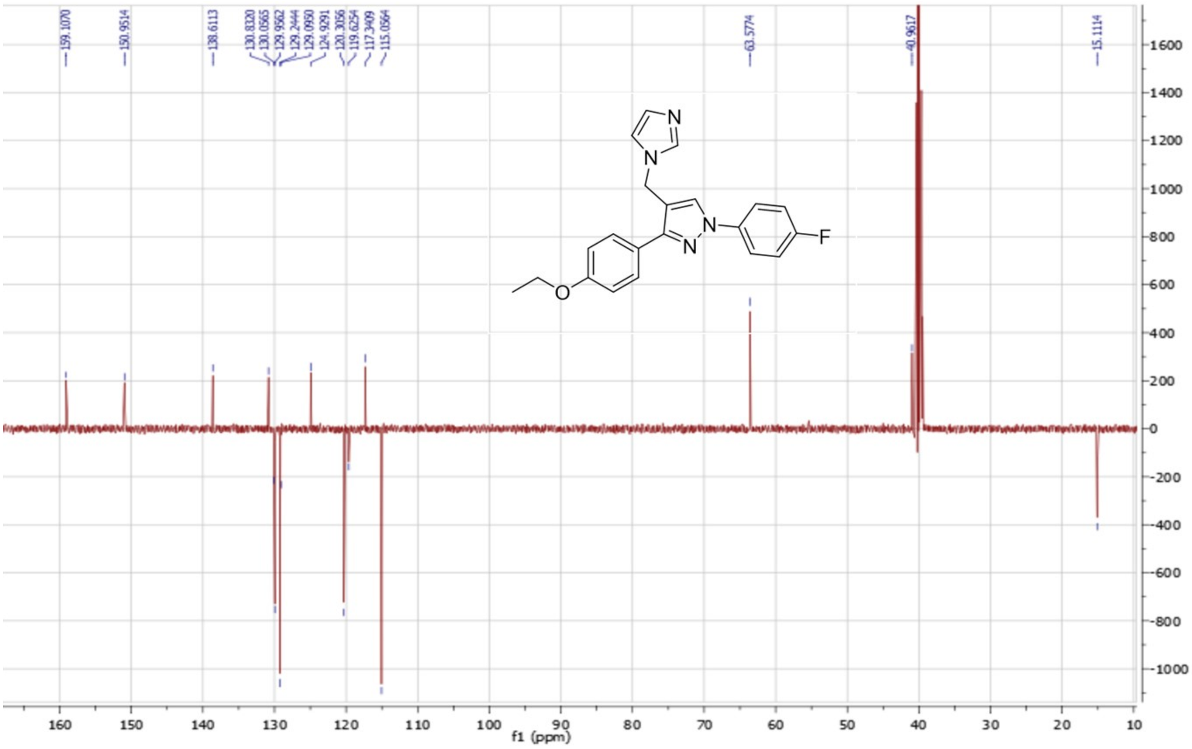

^{19}F NMR (470 MHz, DMSO-d$_6$) of **11c**:

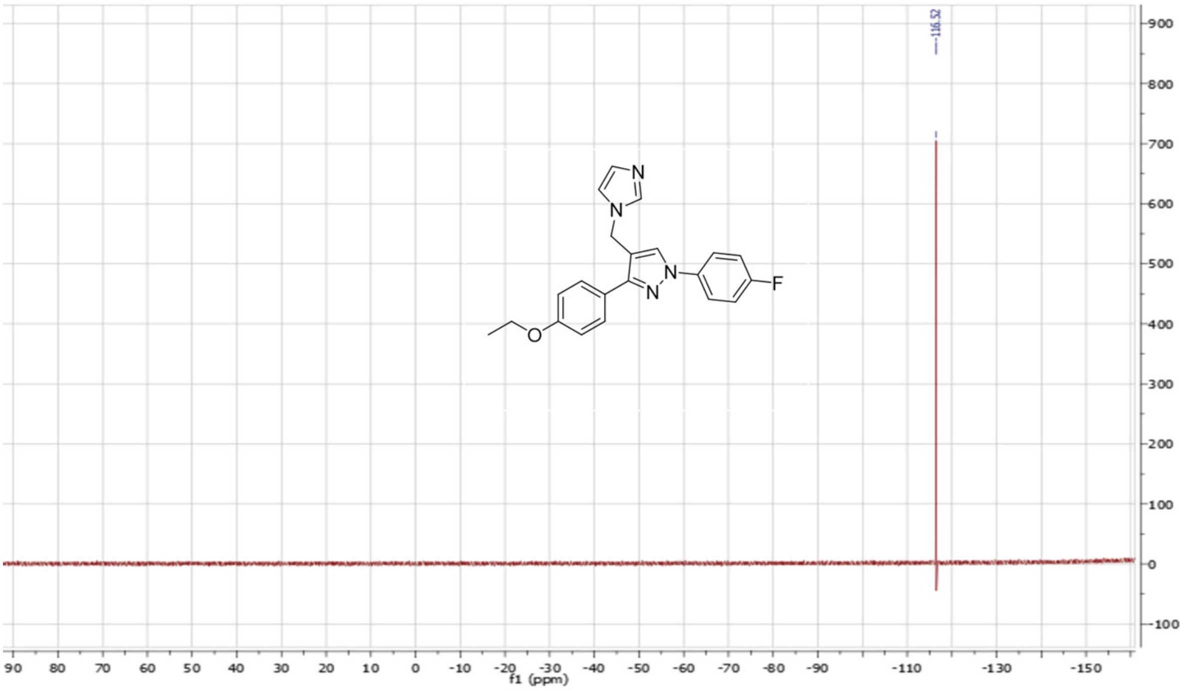

^{1}H NMR spectrum (500 MHz, DMSO-d_6) of **11d**:

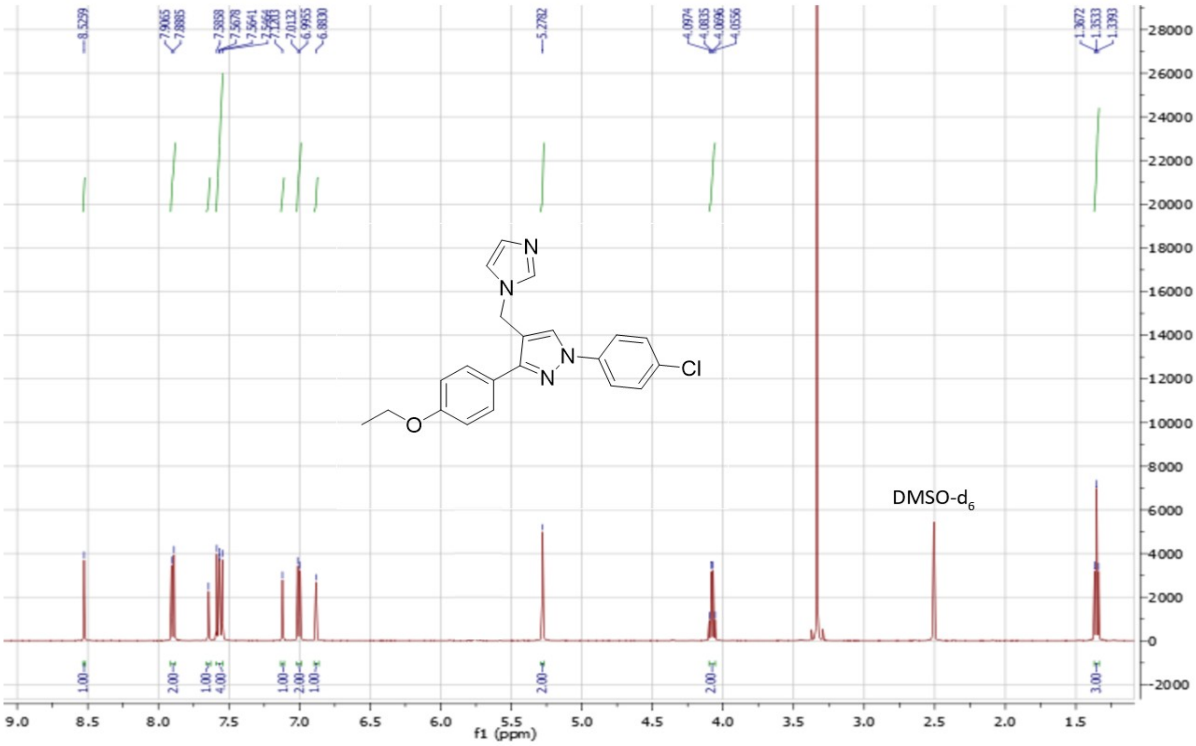

^{13}C NMR spectrum (125.75 MHz, DMSO-d_6) of **11d:**

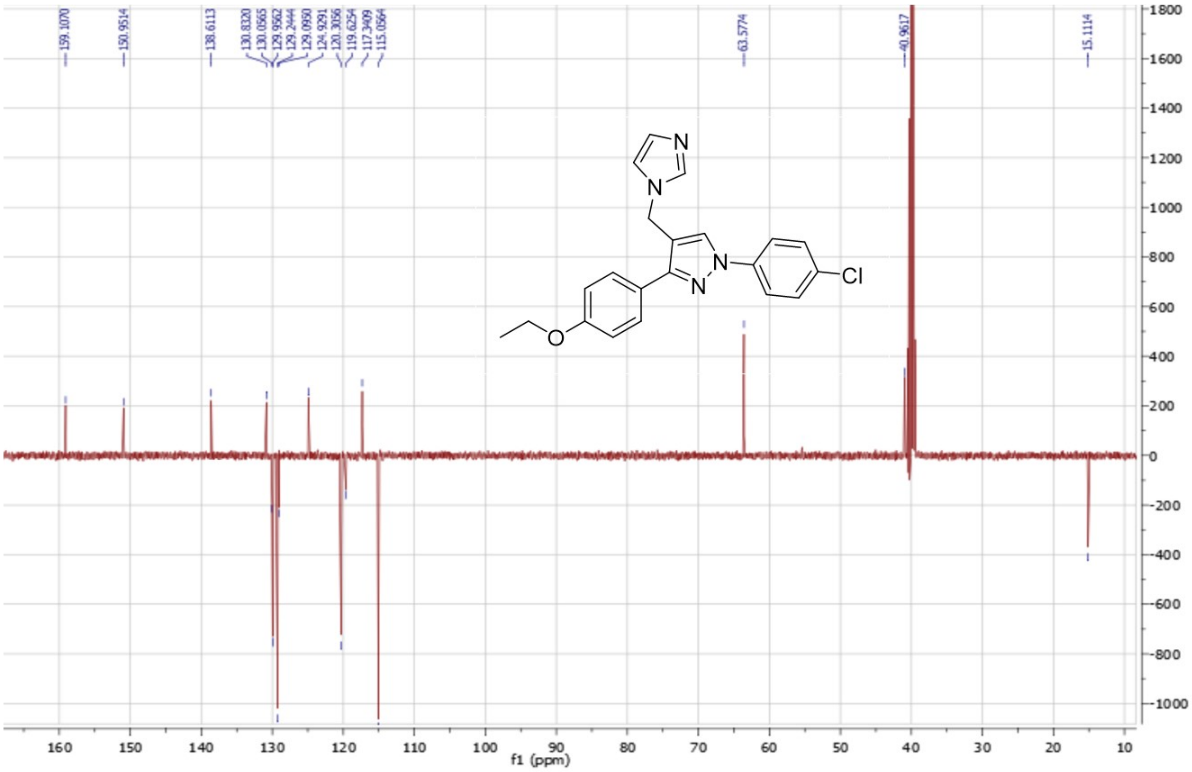

^{1}H NMR spectrum (500 MHz, DMSO-d$_6$) of **11e**:

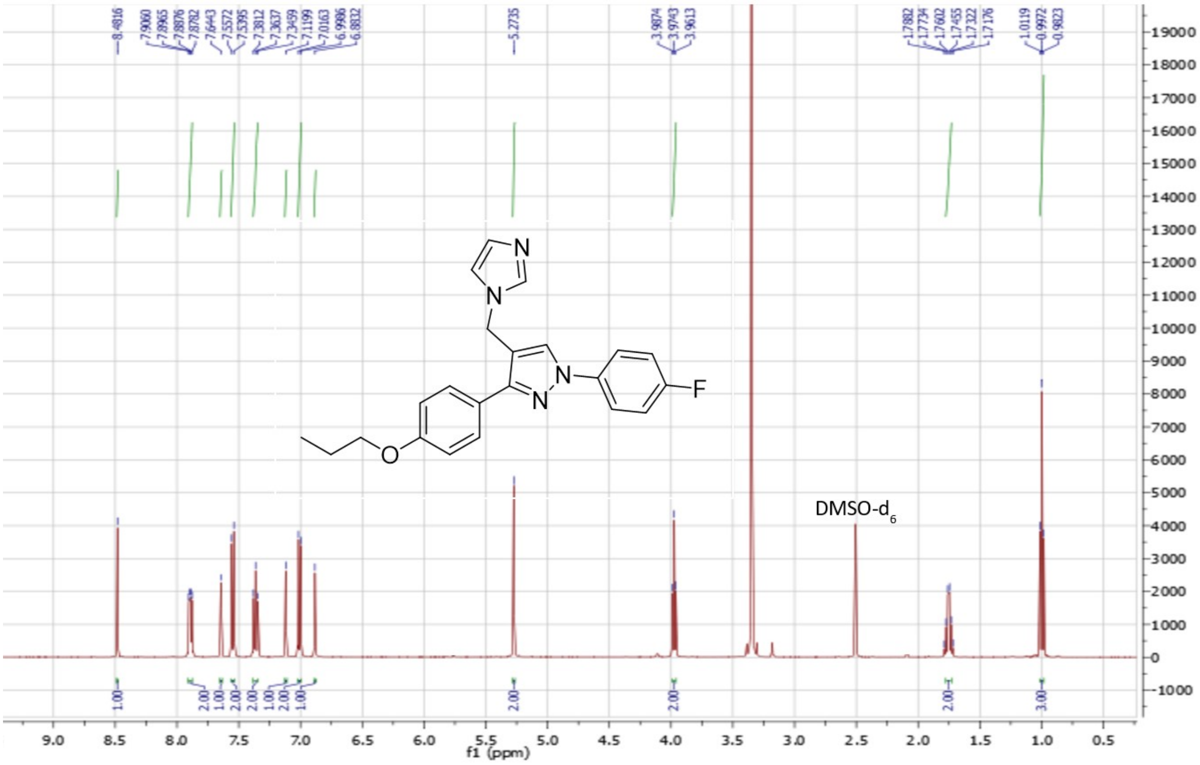

^{13}C NMR spectrum (125.75 MHz, DMSO-d$_6$) of **11e:**

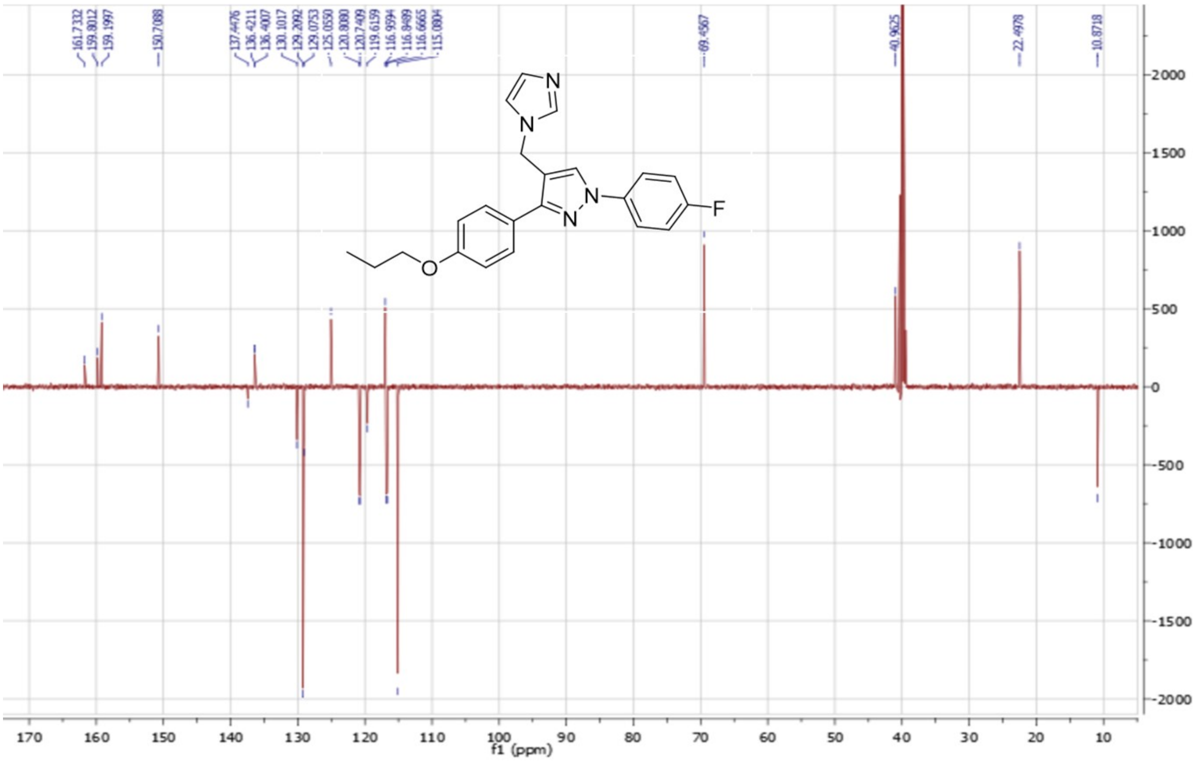

^{19}F NMR (470 MHz, DMSO-d$_6$) of **11e**:

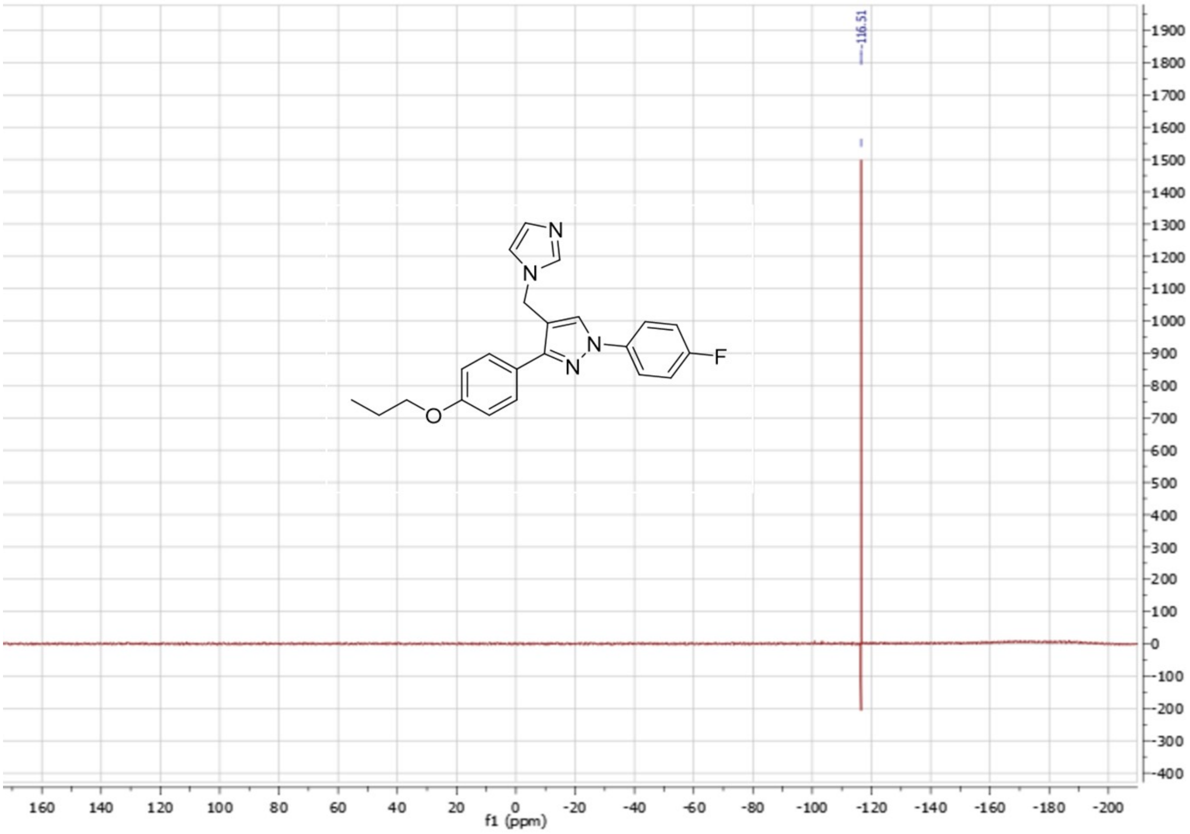

^{1}H NMR spectrum (500 MHz, DMSO-d$_6$) of **11f**:

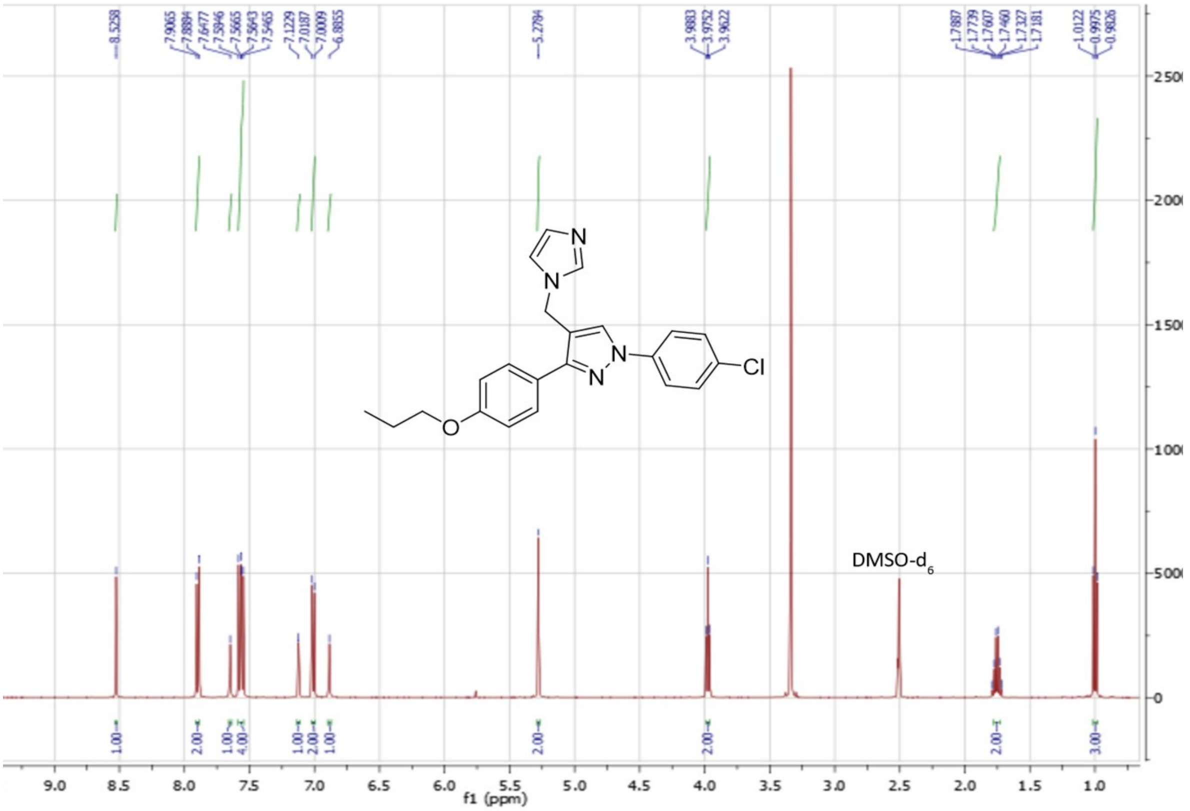

^{13}C NMR spectrum (125.75 MHz, DMSO-d$_6$) of **11f:**

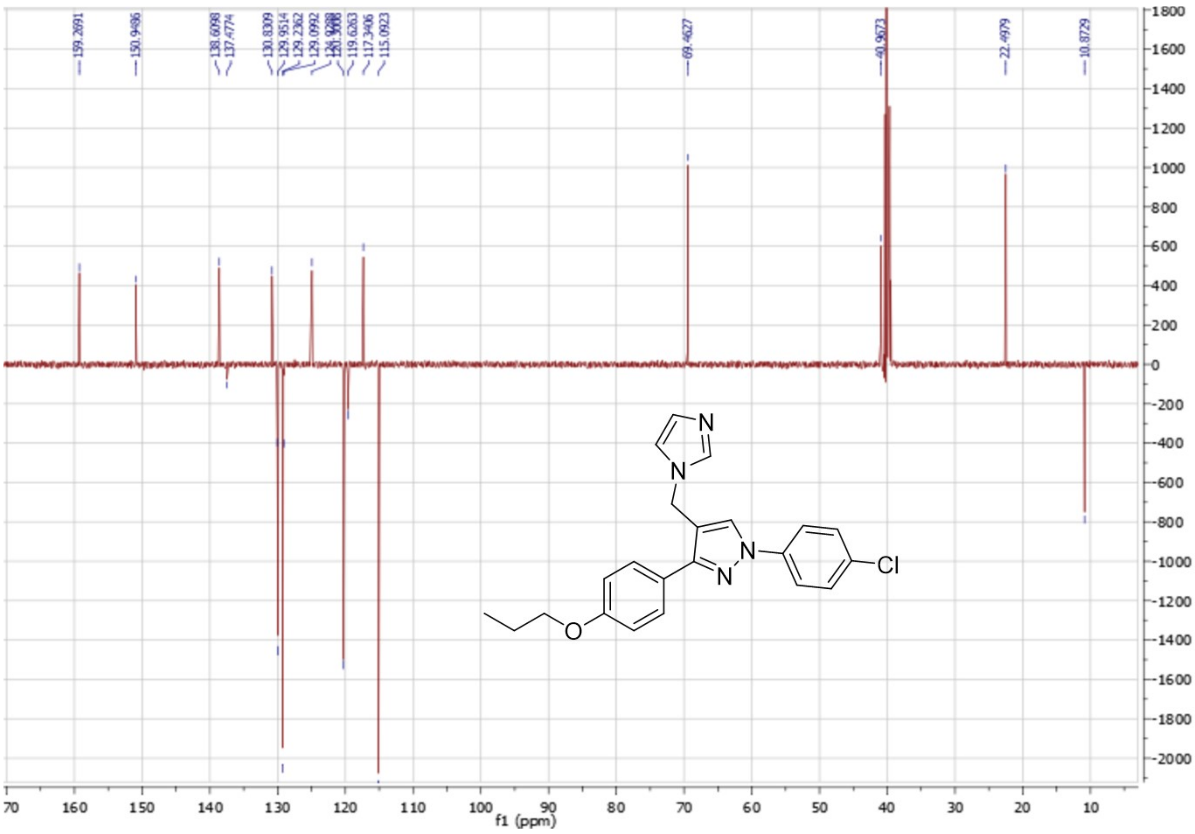

^{1}H NMR spectrum (500 MHz, DMSO-d_6) of **11g**:

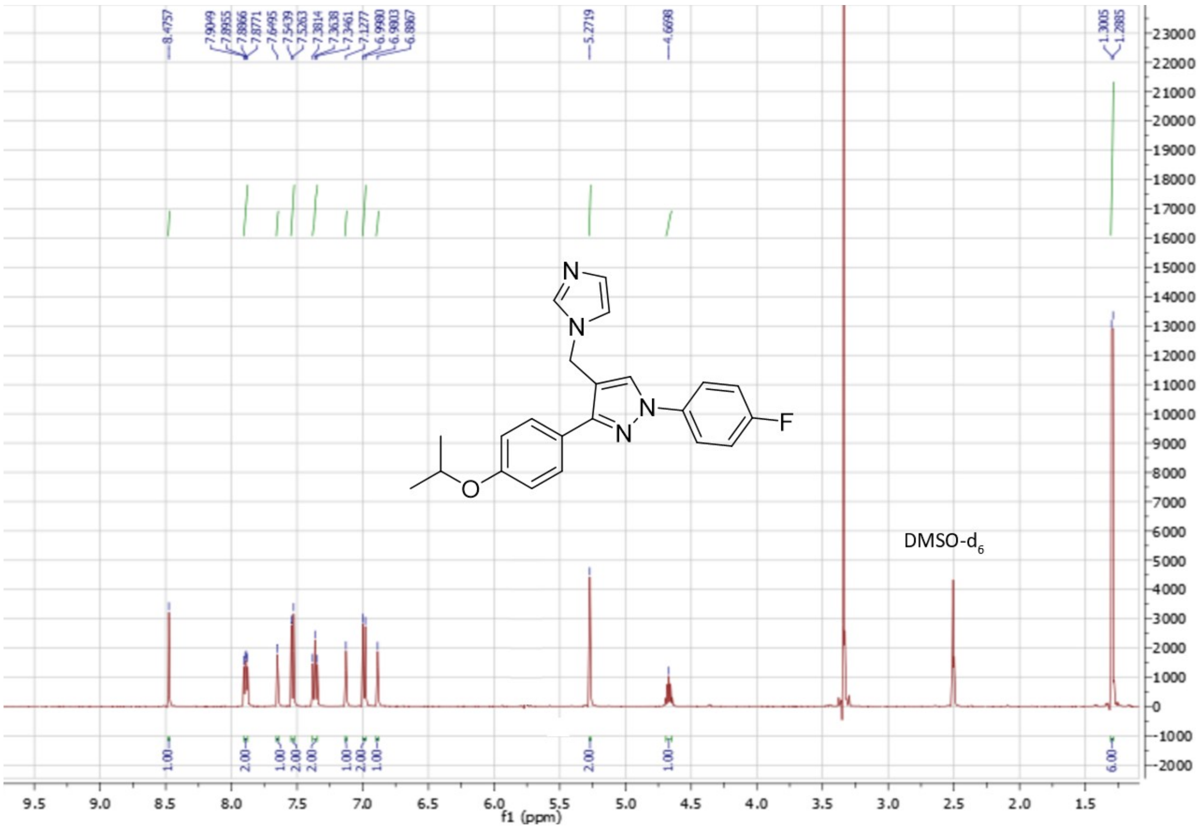

^{13}C NMR spectrum (125.75 MHz, DMSO-d_6) of **11g:**

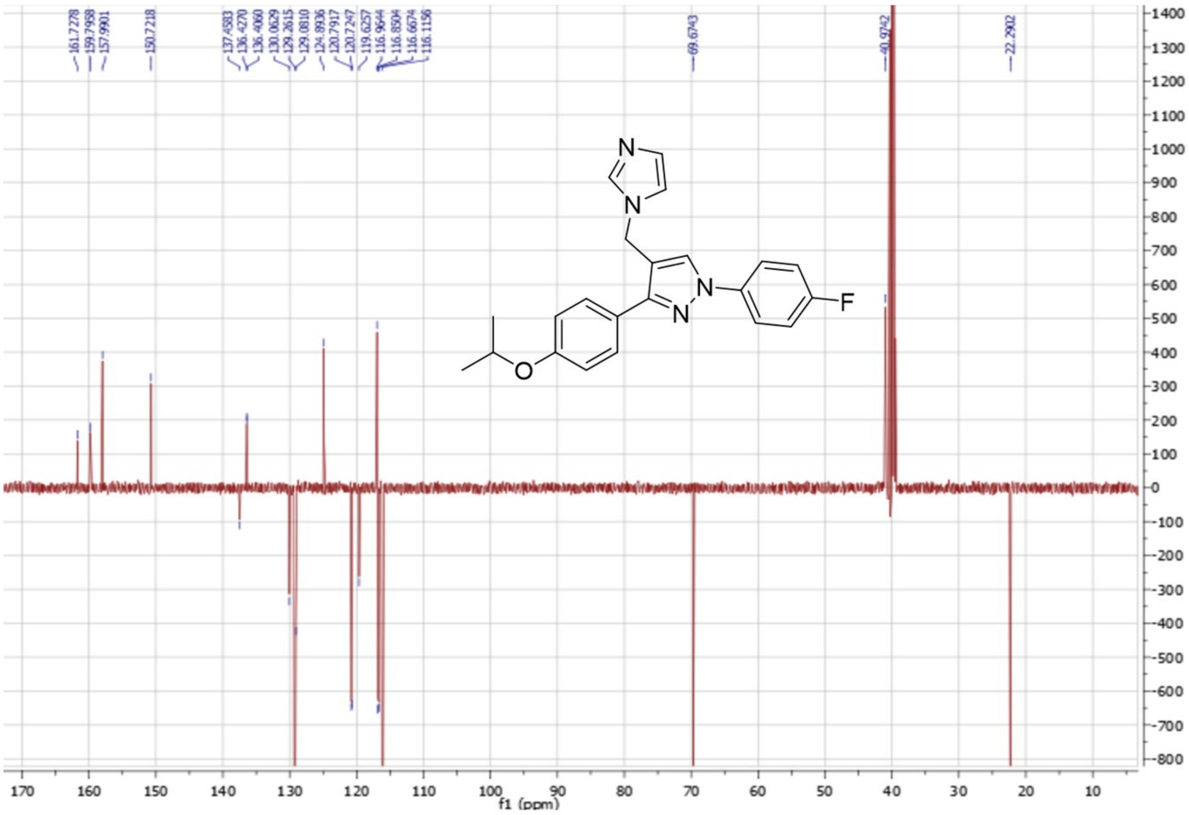

^{19}F NMR (470 MHz, DMSO-d$_6$) of **11g**:

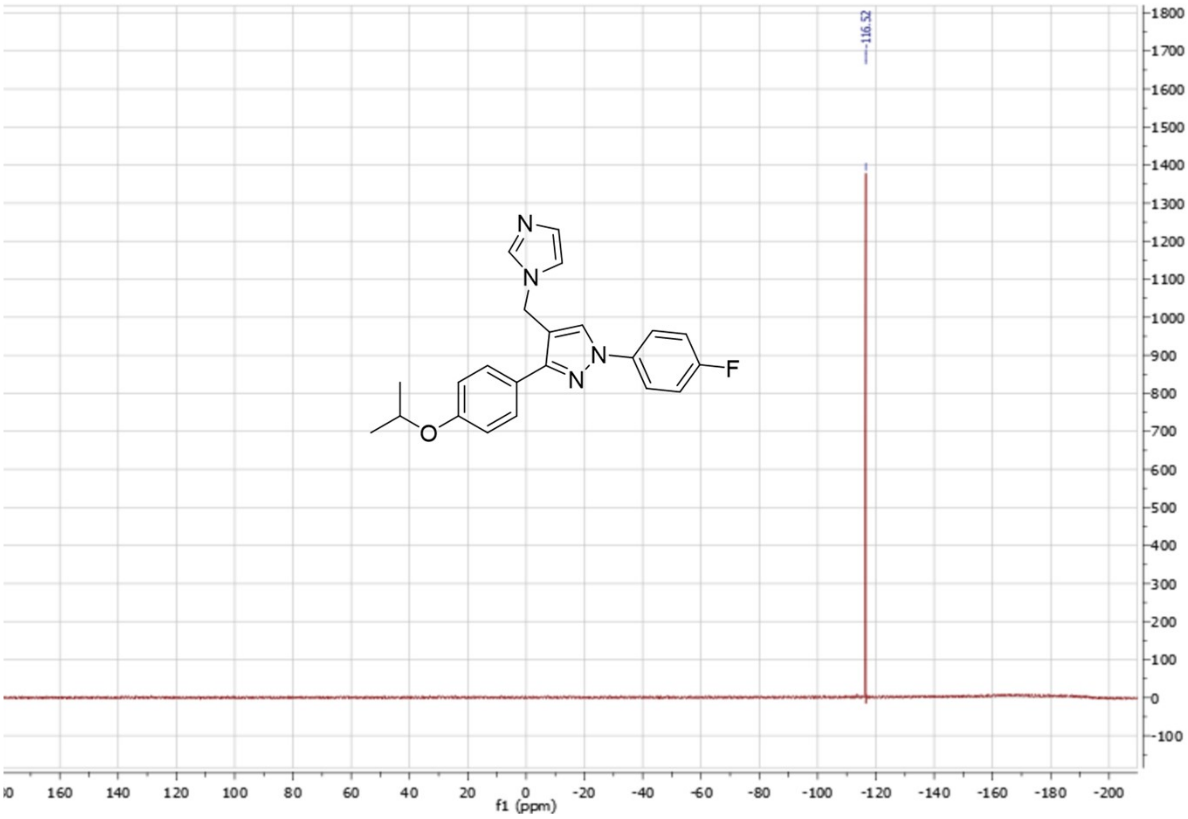

^{1}H NMR spectrum (500 MHz, DMSO-d$_6$) of **11h**:

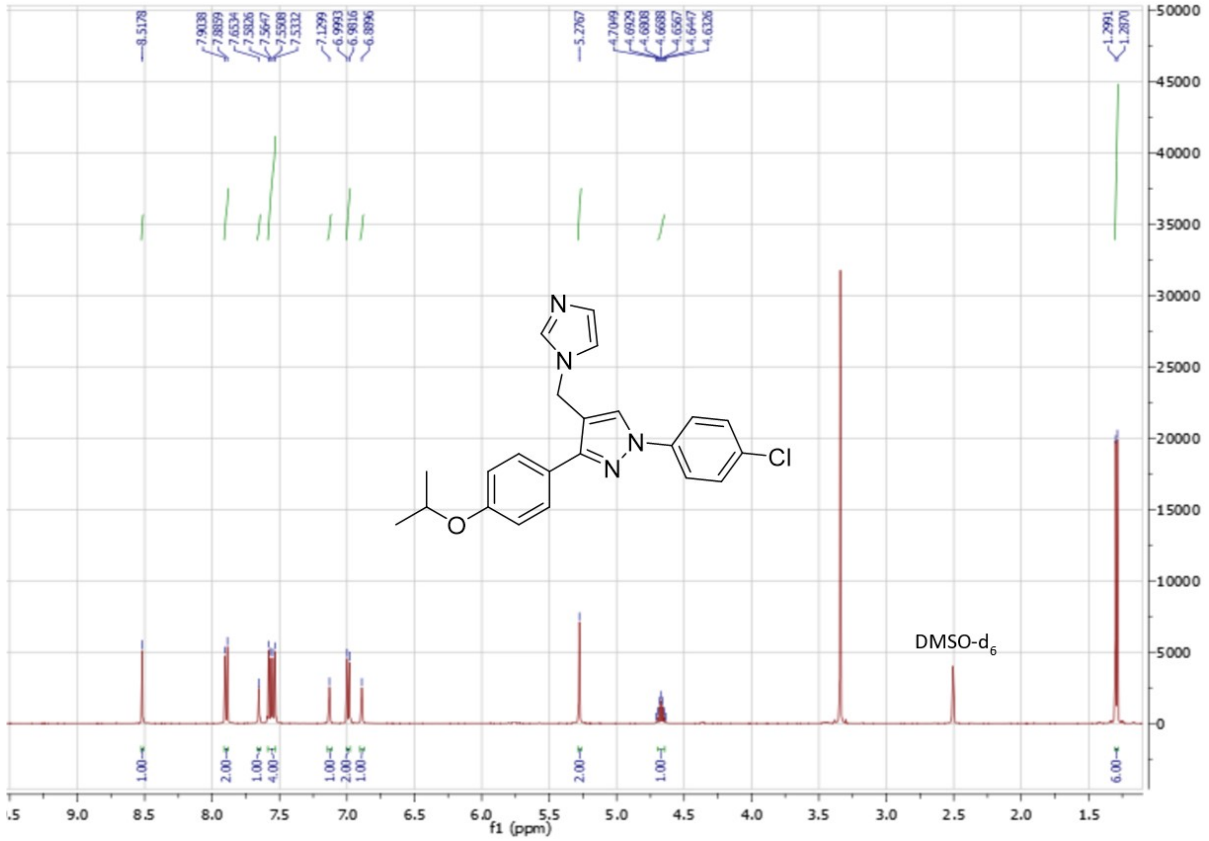

^{13}C NMR spectrum (125.75 MHz, DMSO-d$_6$) of **11h:**

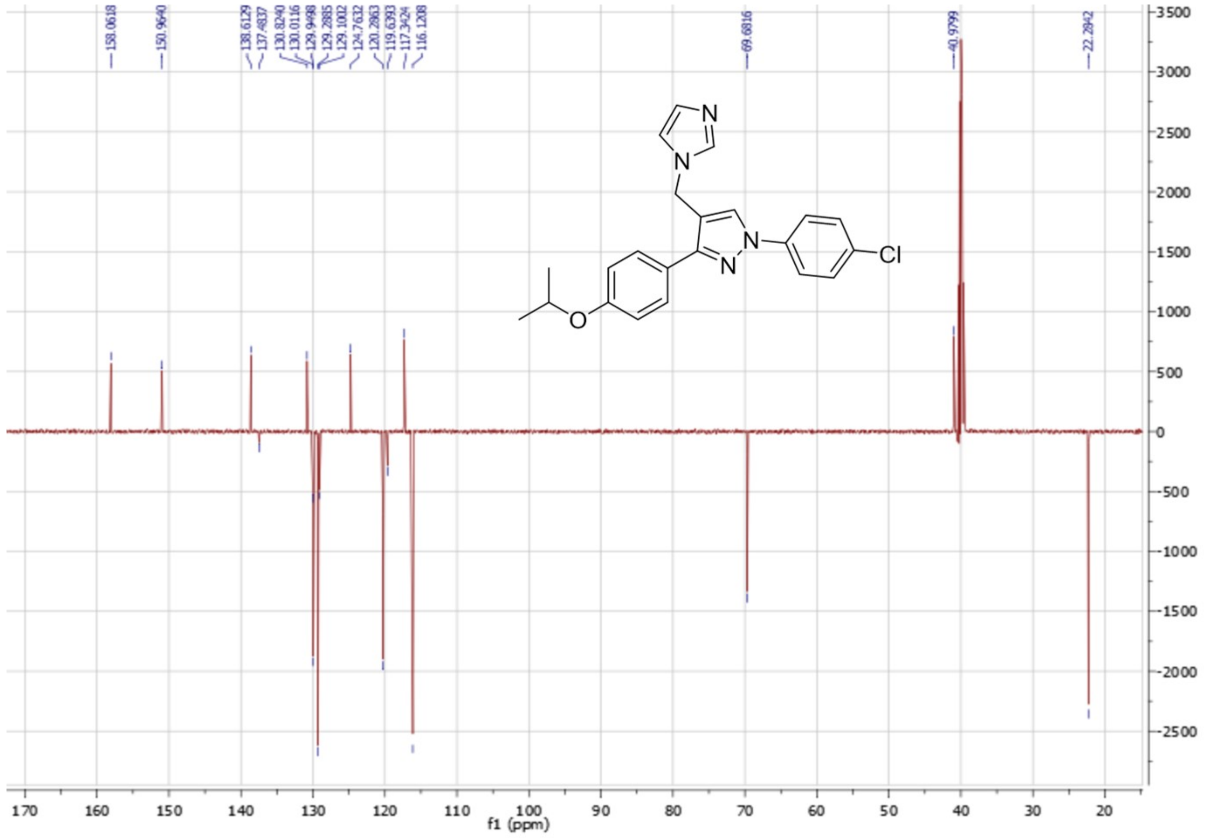

^{1}H NMR spectrum (500 MHz, DMSO-d$_6$) of **12a**:

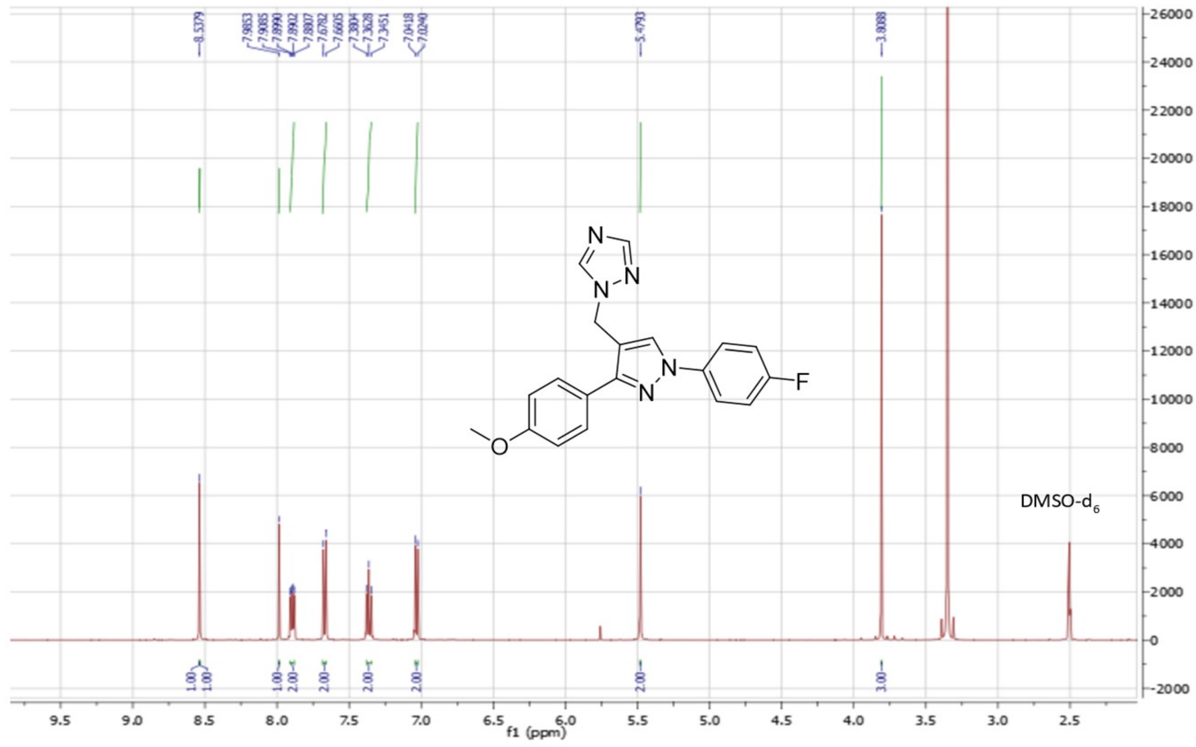

^{13}C NMR spectrum (125.75 MHz, DMSO-d$_6$) of **12a:**

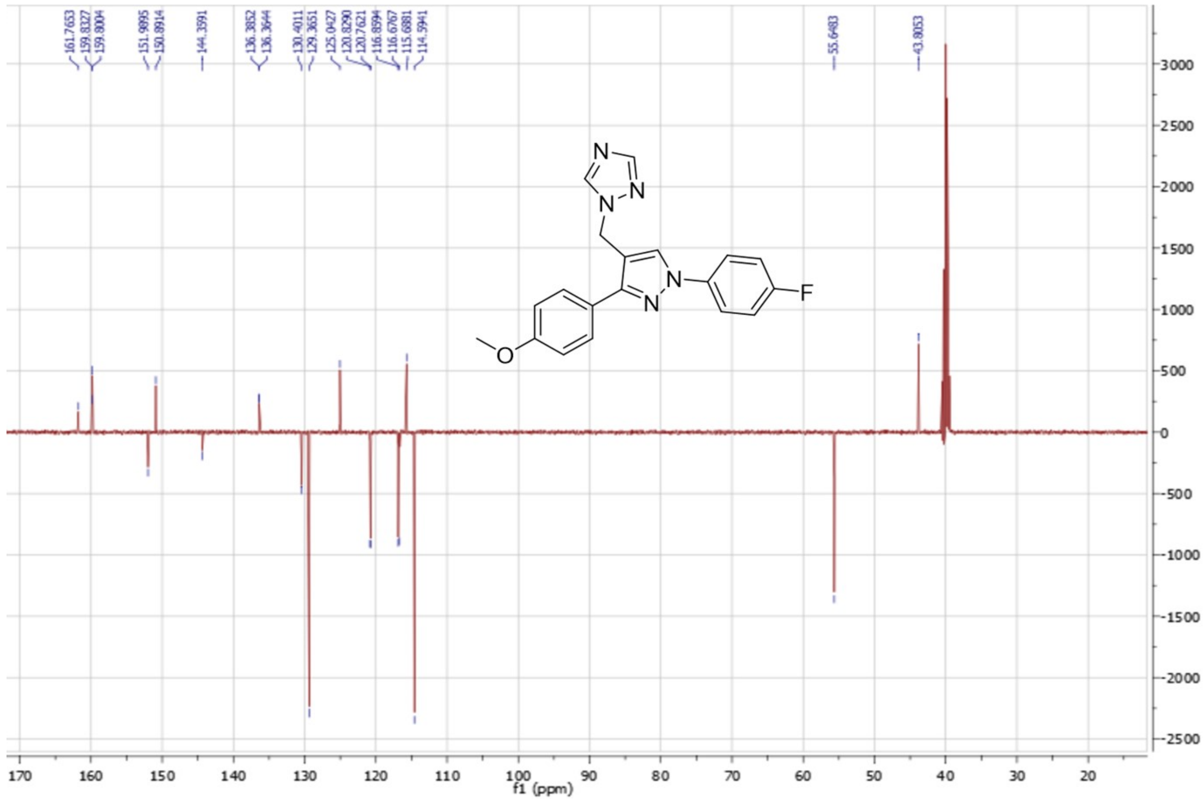

^{19}F NMR (470 MHz, DMSO-d$_6$) of **12a**:

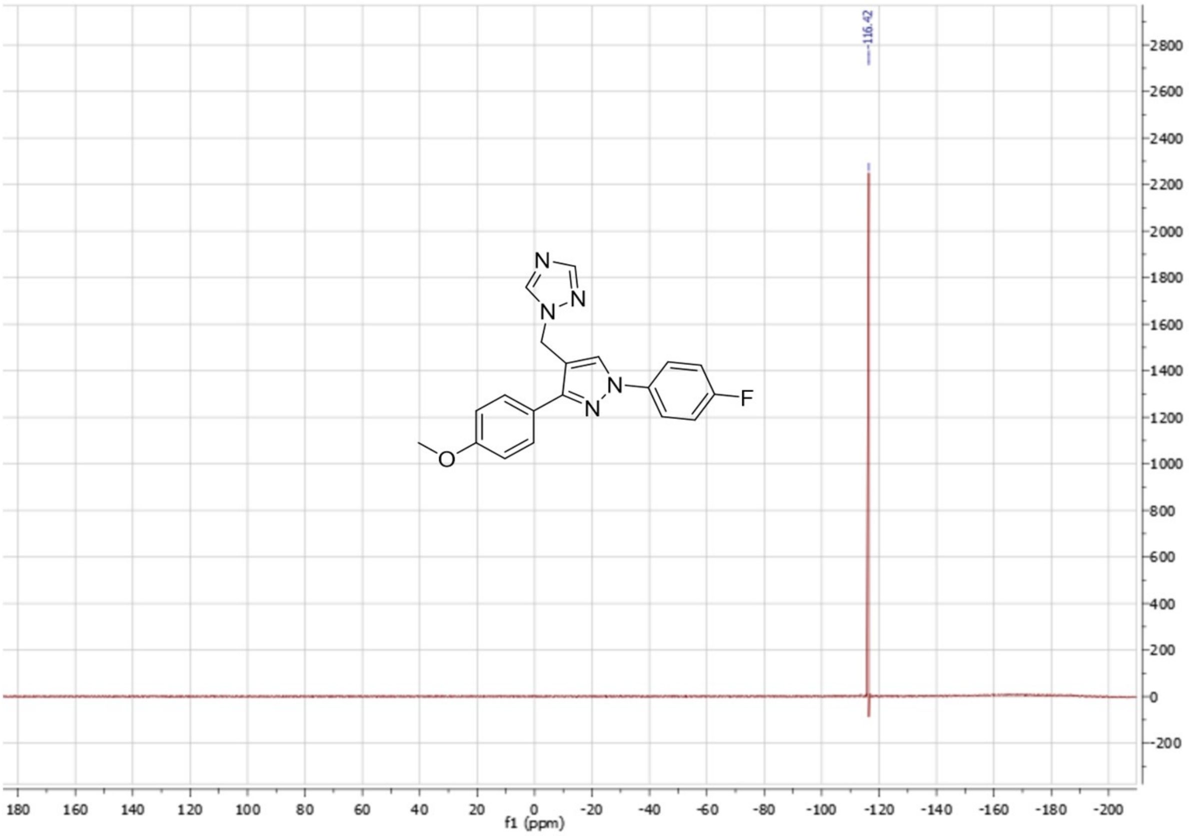

^{1}H NMR spectrum (500 MHz, DMSO-d_6) of **12b**:

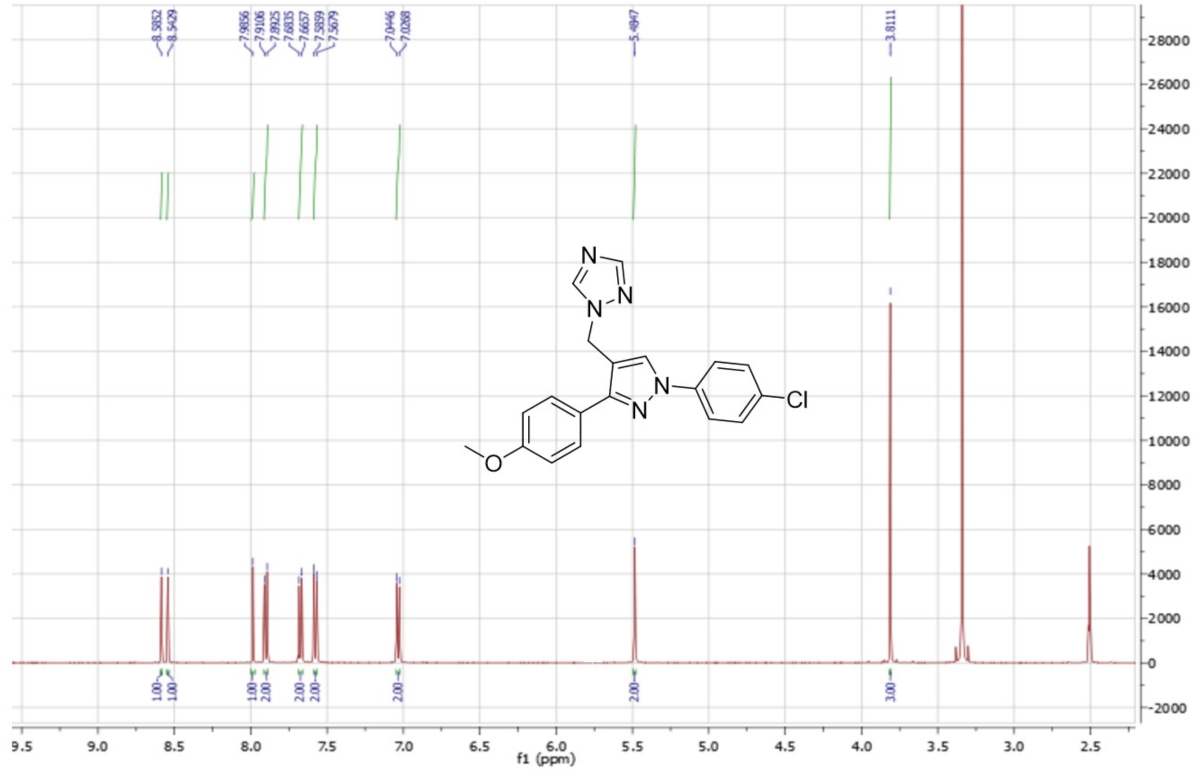

^{13}C NMR spectrum (125.75 MHz, DMSO-d_6) of **12b:**

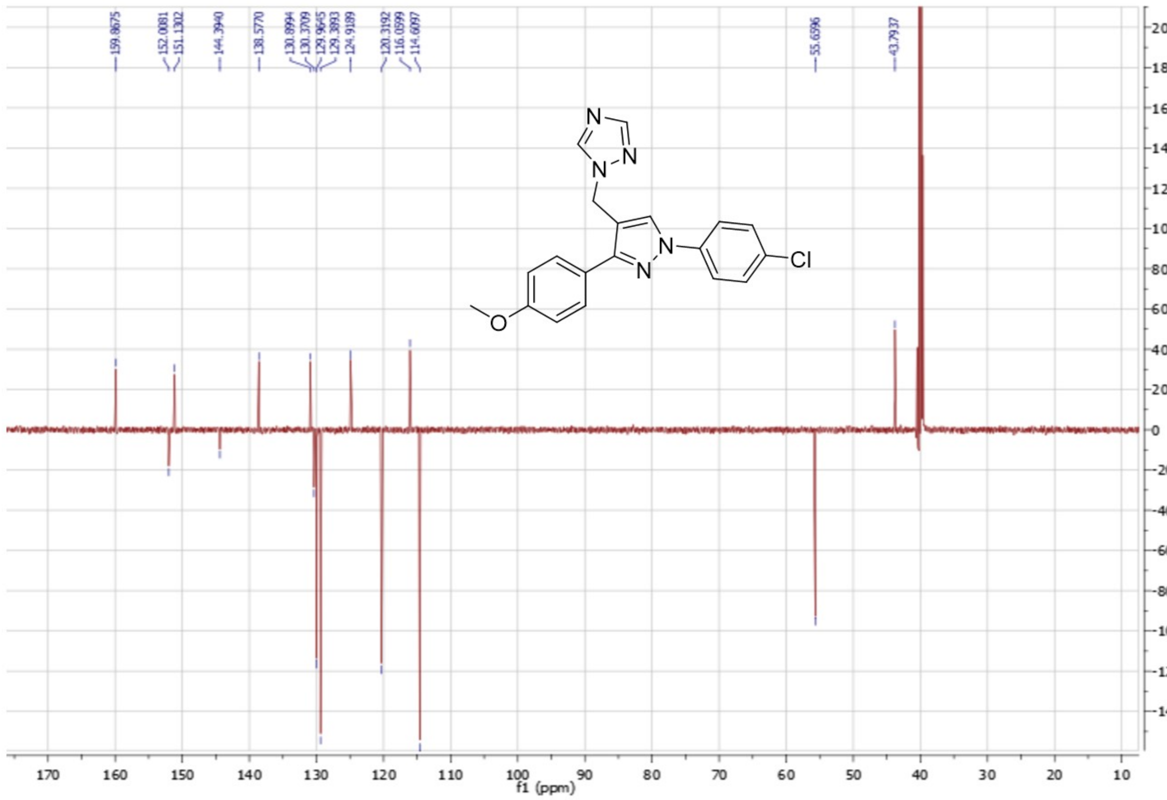

^{1}H NMR spectrum (500 MHz, DMSO-d_6) of **12c**:

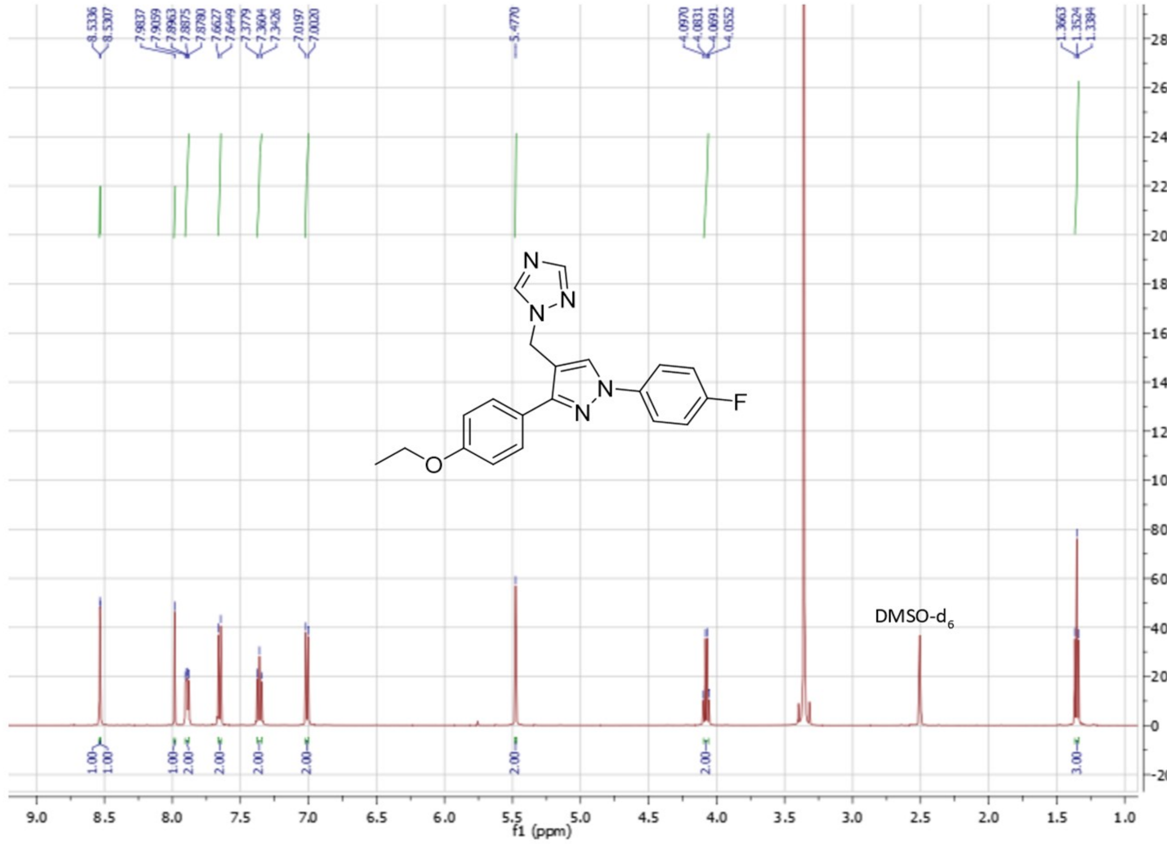

^{13}C NMR spectrum (125.75 MHz, DMSO-d_6) of **12c:**

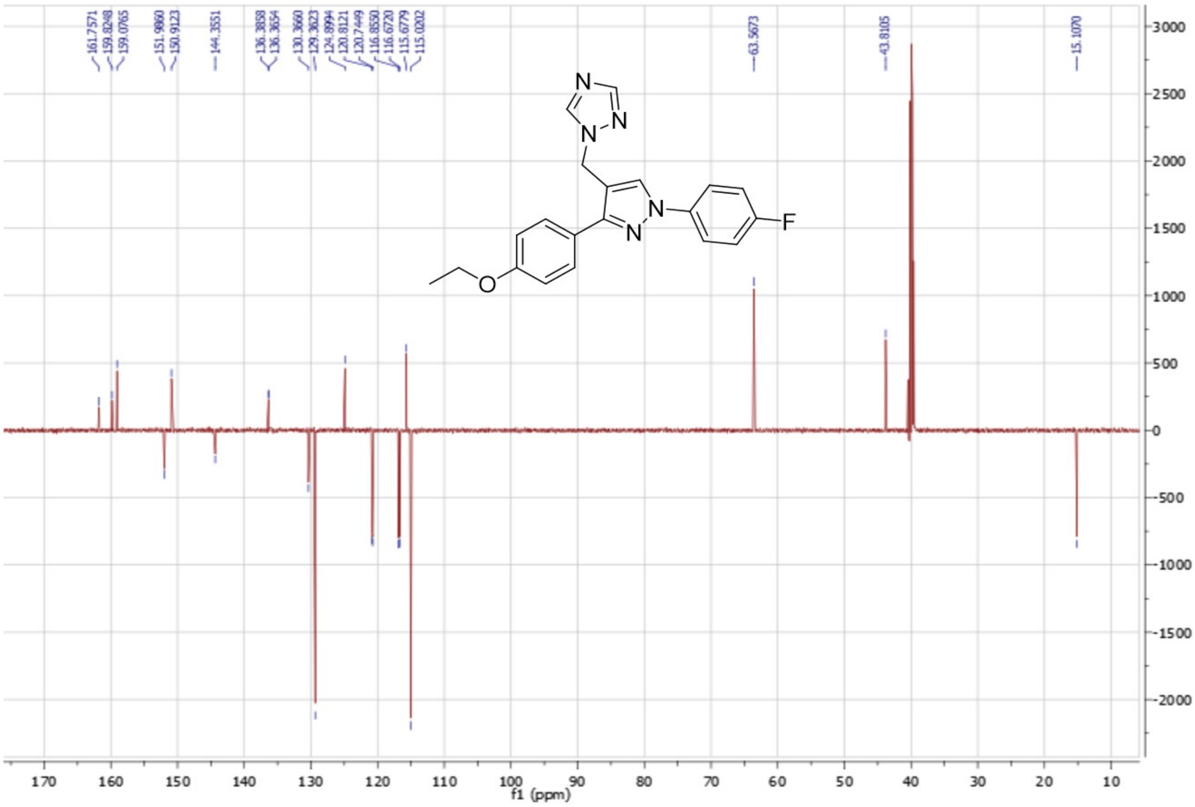

^{19}F NMR (470 MHz, DMSO-d$_6$) of **12c**:

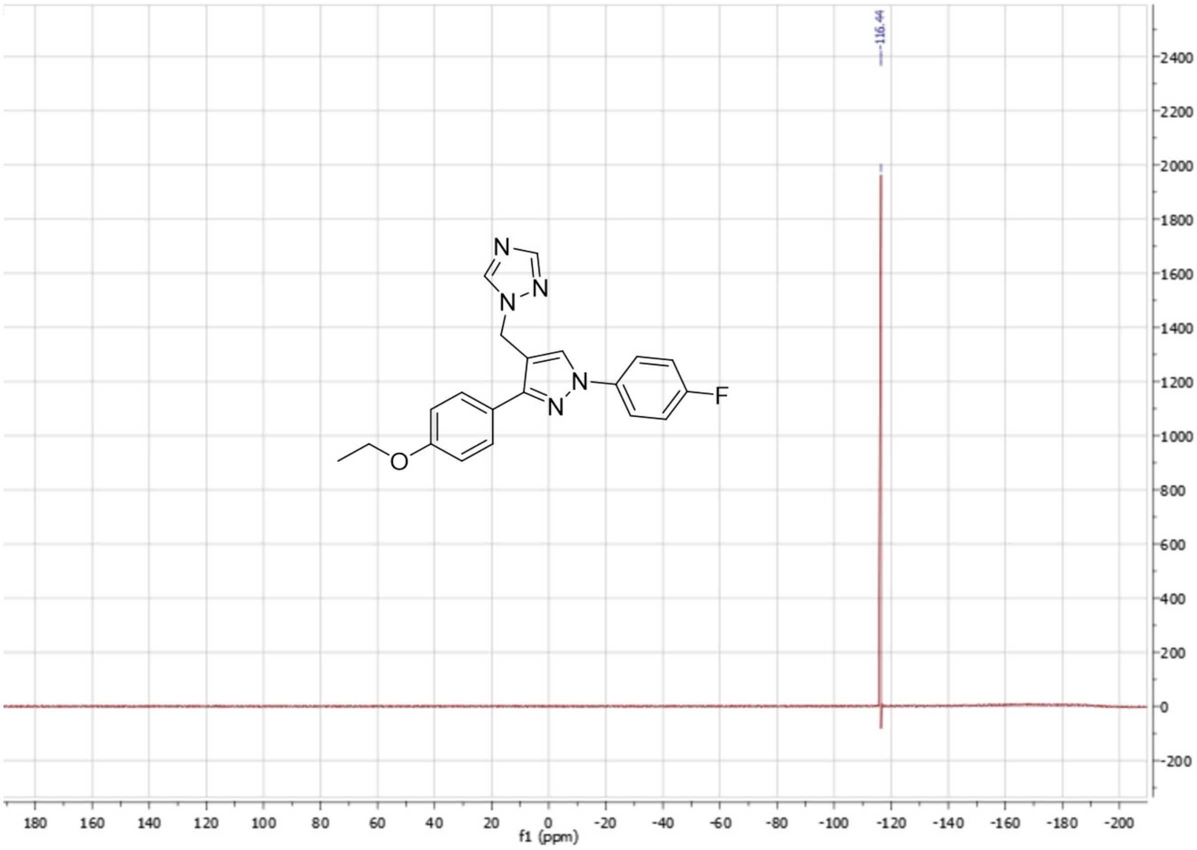

^{1}H NMR spectrum (500 MHz, DMSO-d$_6$) of **12d**:

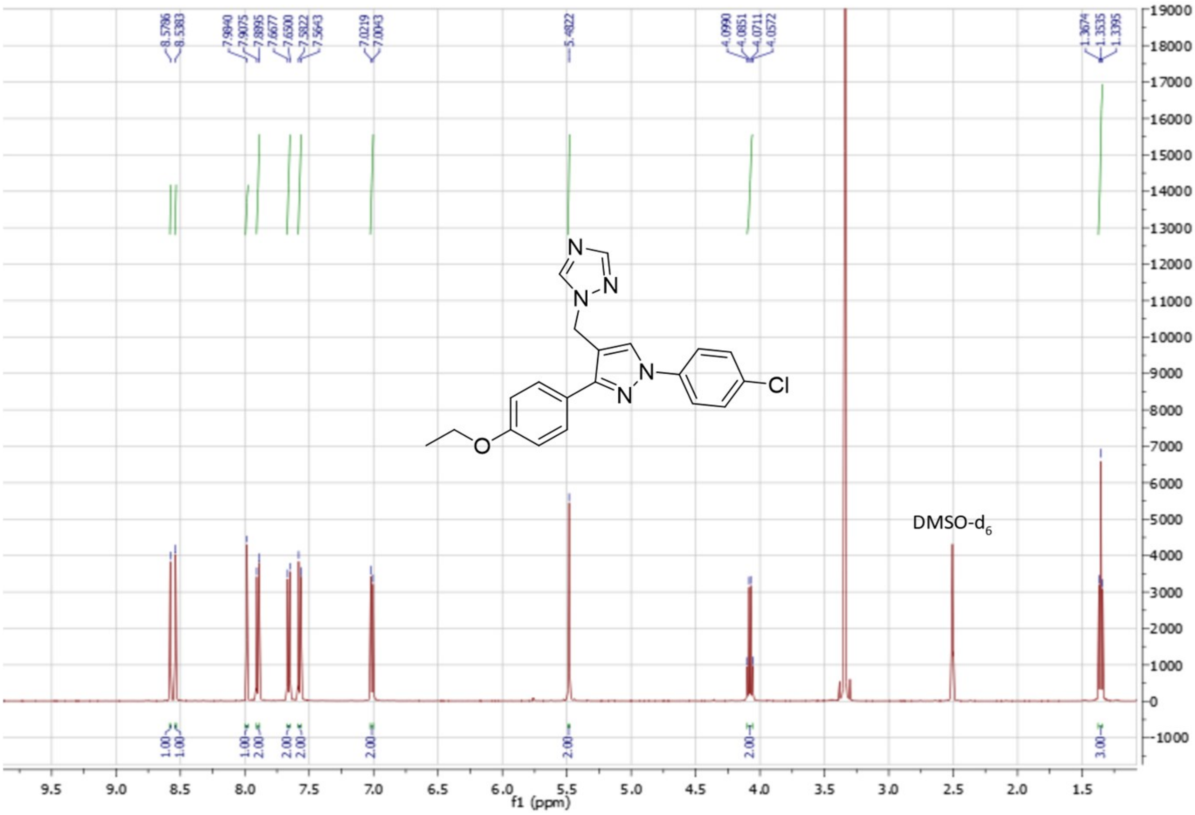

^{13}C NMR spectrum (125.75 MHz, DMSO-d_6) of **12d:**

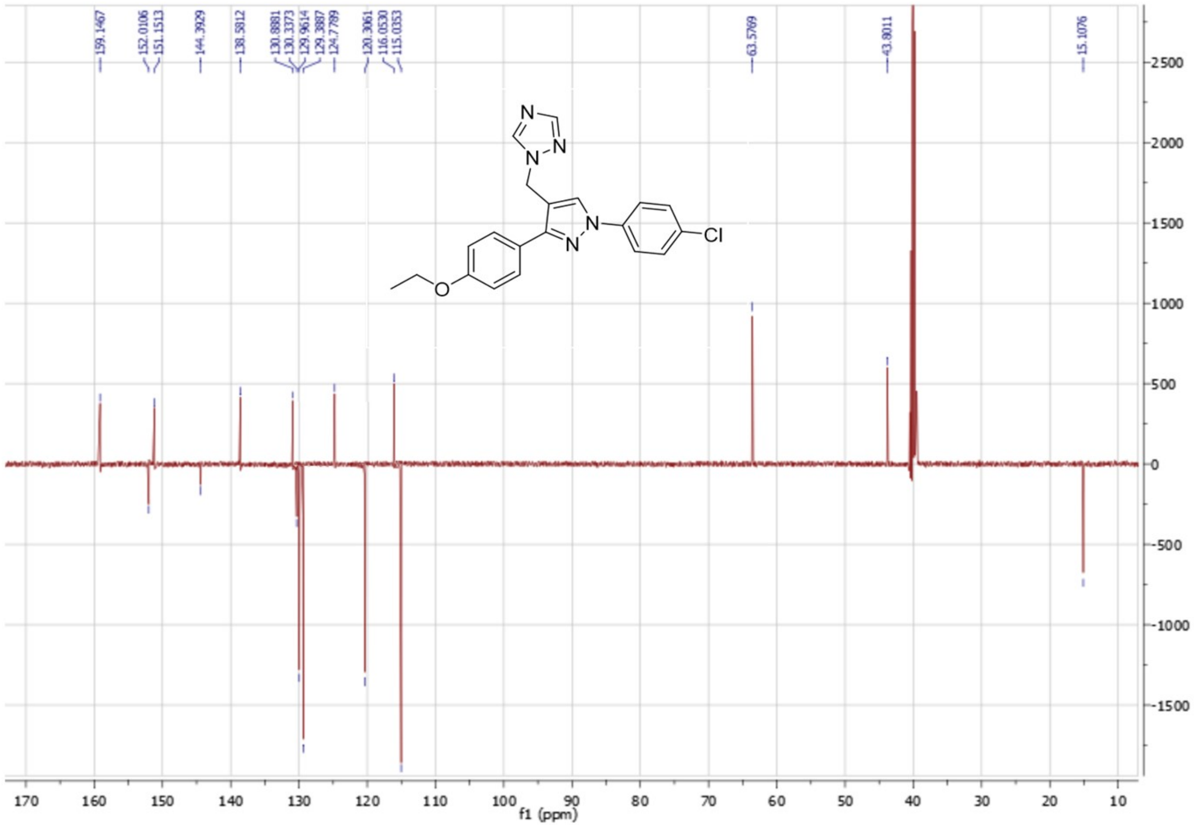

^{1}H NMR spectrum (500 MHz, DMSO-d_6) of **12e**:

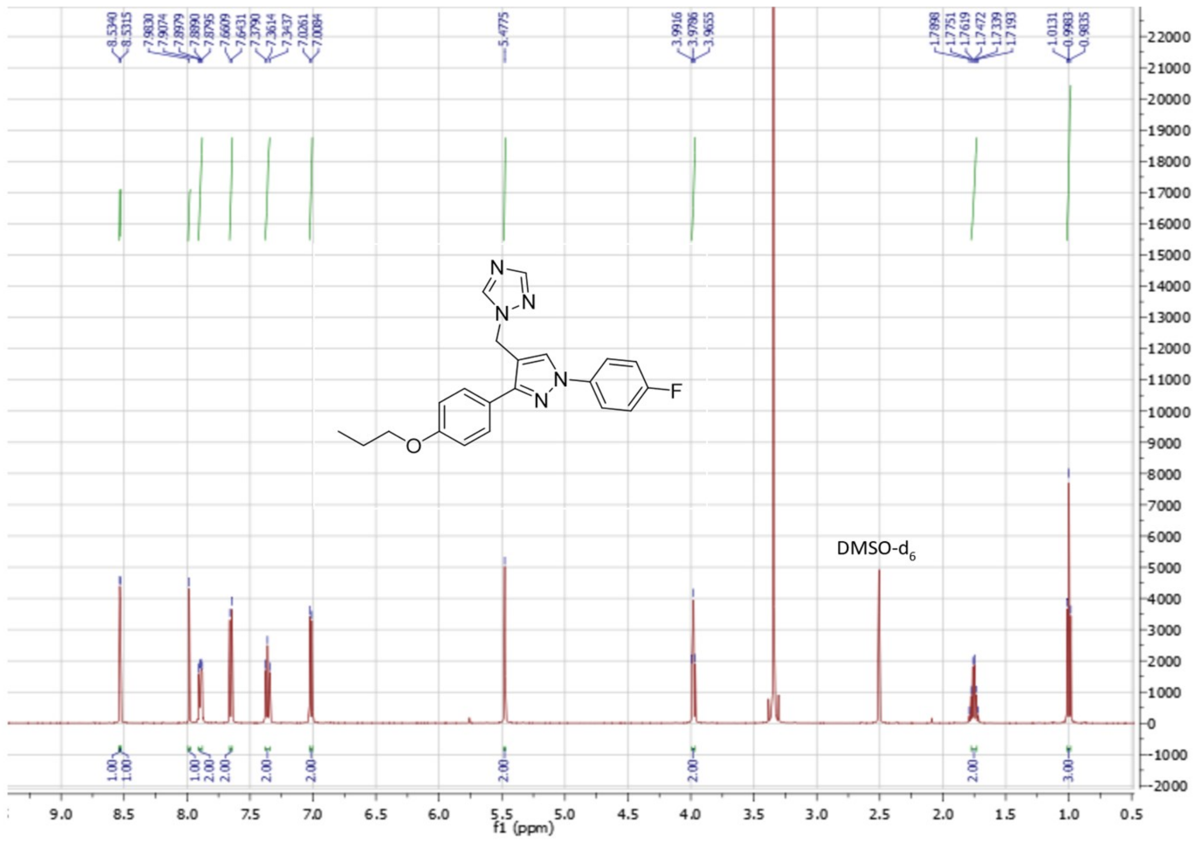

^{13}C NMR spectrum (125.75 MHz, DMSO-d$_6$) of **12e:**

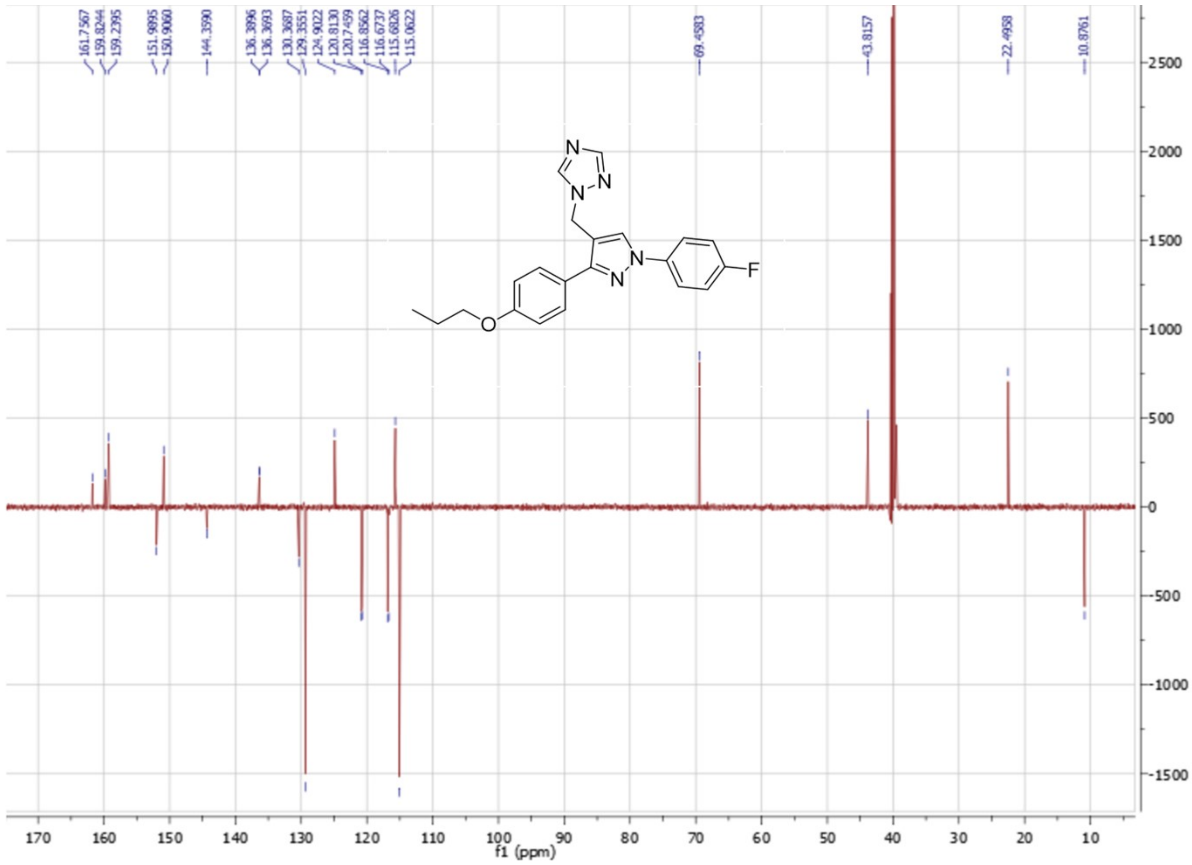

^{19}F NMR (470 MHz, DMSO-d$_6$) of **12e**:

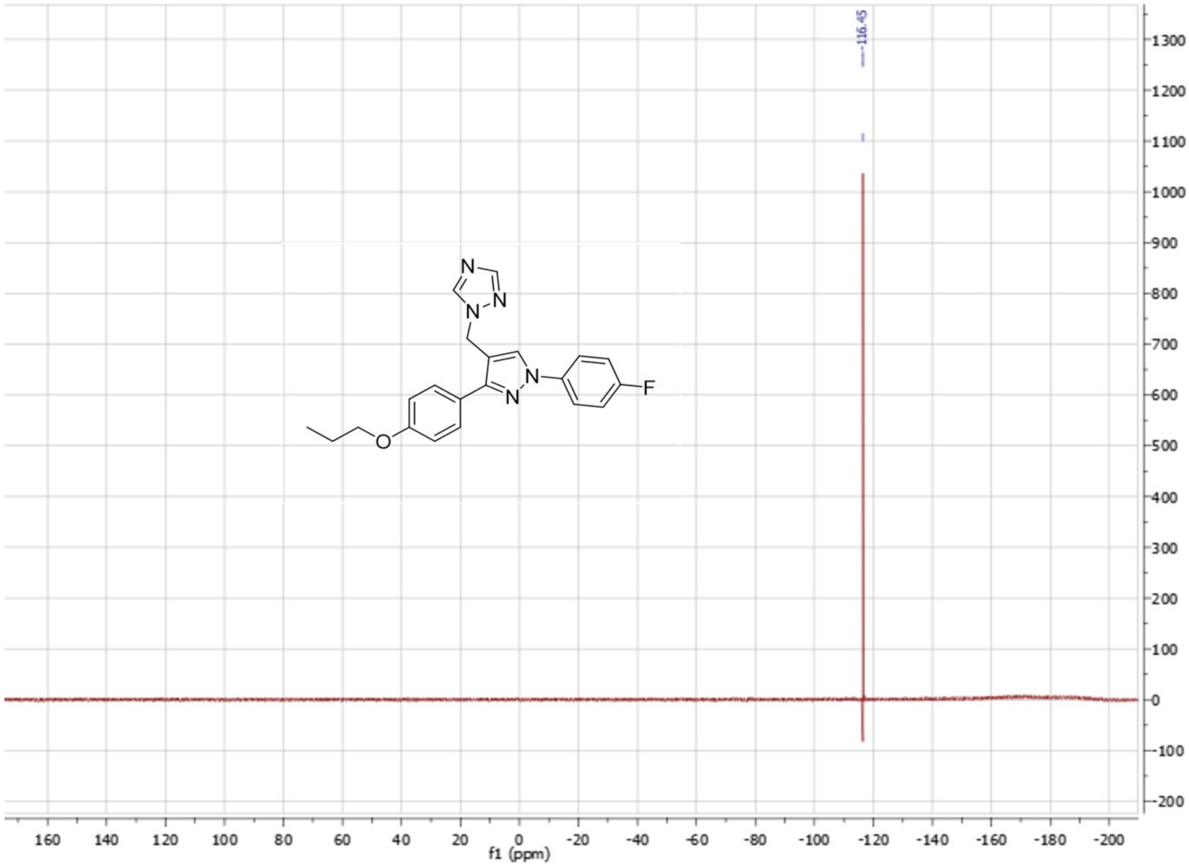

^{1}H NMR spectrum (500 MHz, DMSO-d_6) of **12f**:

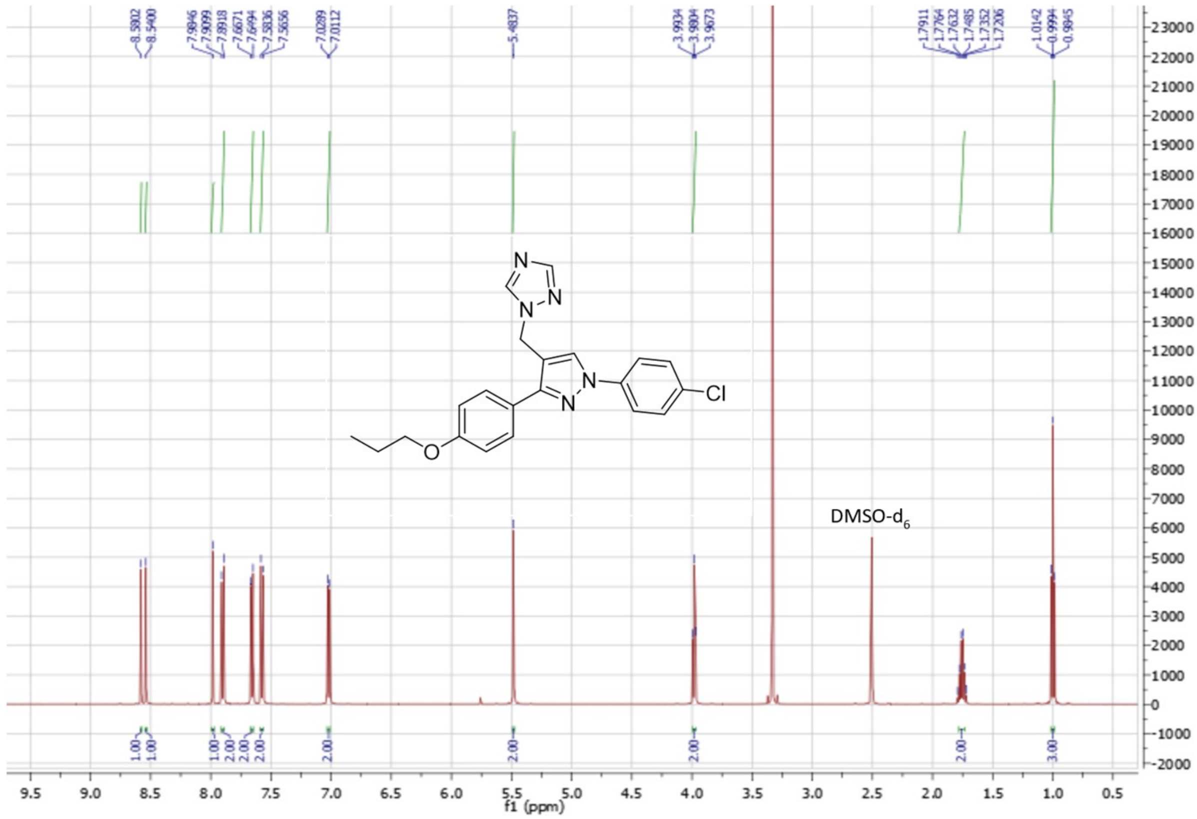

^{13}C NMR spectrum (125.75 MHz, DMSO-d_6) of **12f:**

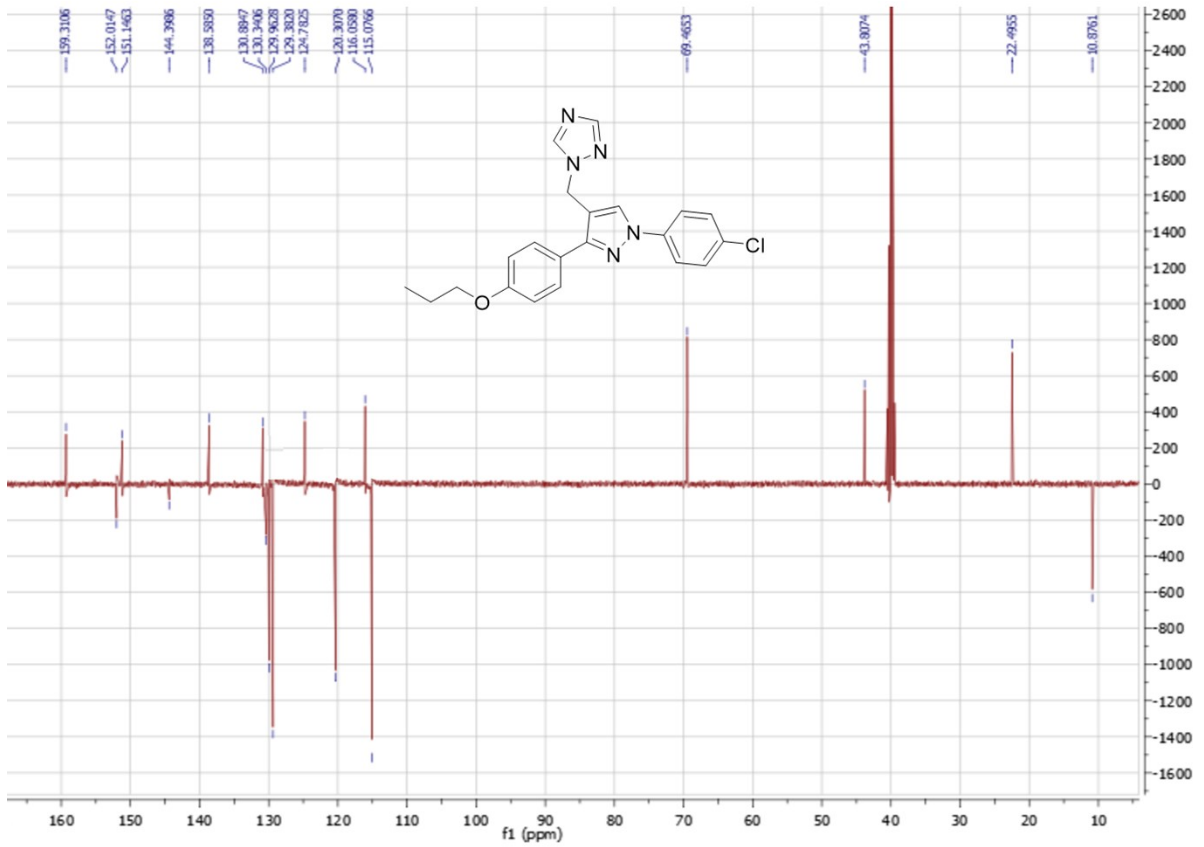

^{1}H NMR spectrum (500 MHz, DMSO-d_6) of **12g**:

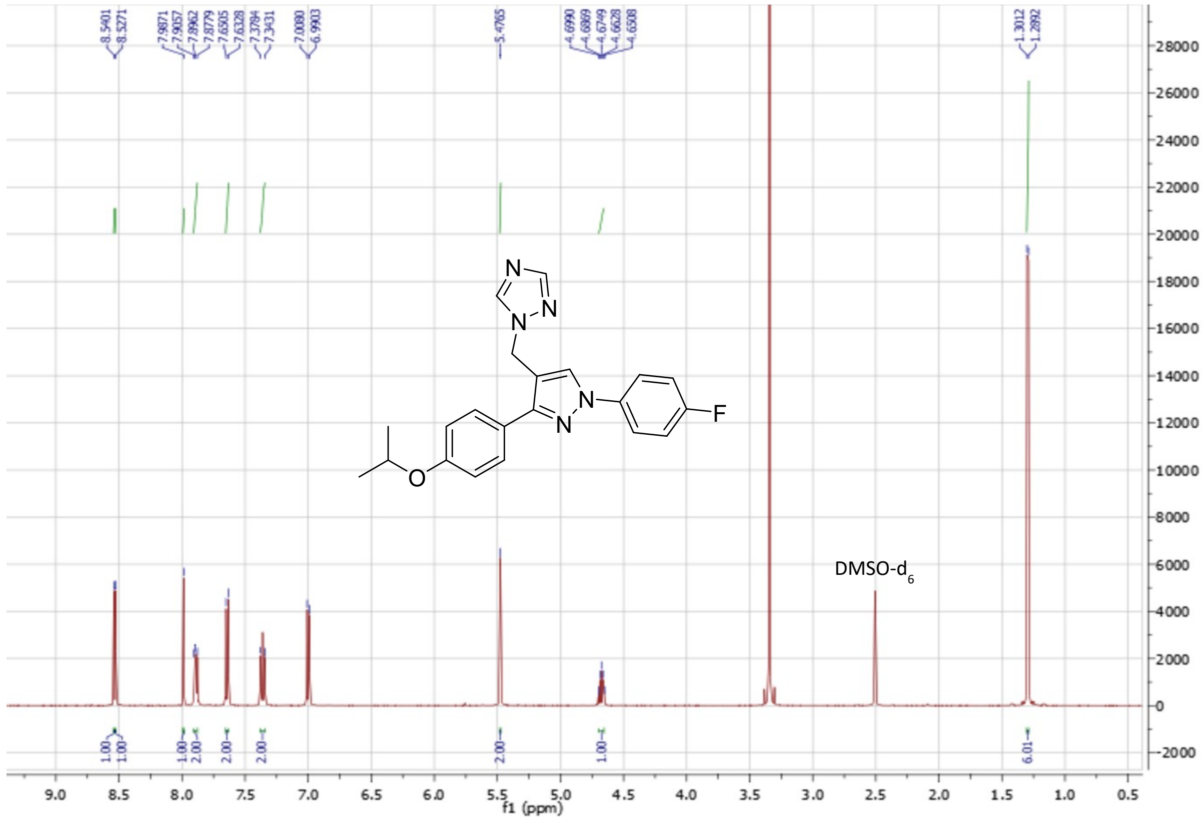

^{13}C NMR spectrum (125.75 MHz, DMSO-d_6) of **12g:**

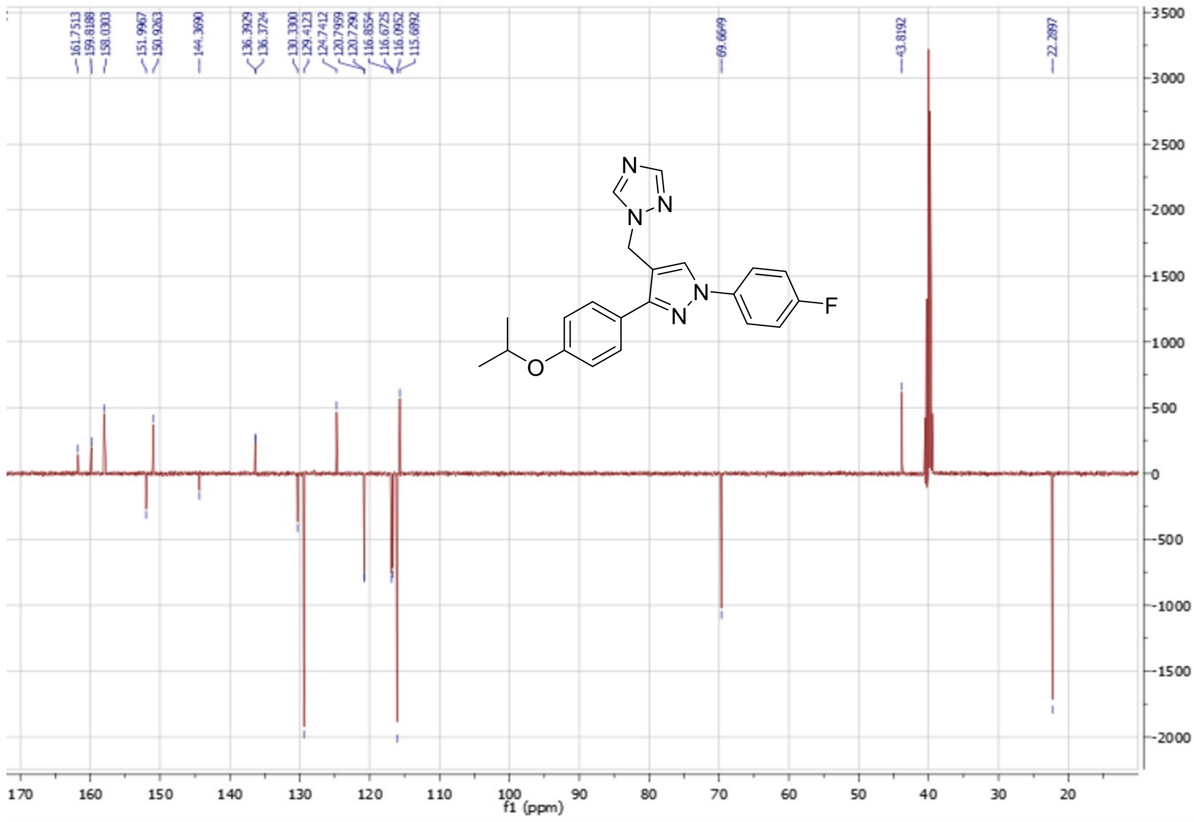

^{19}F NMR (470 MHz, DMSO-d$_6$) of **12g**:

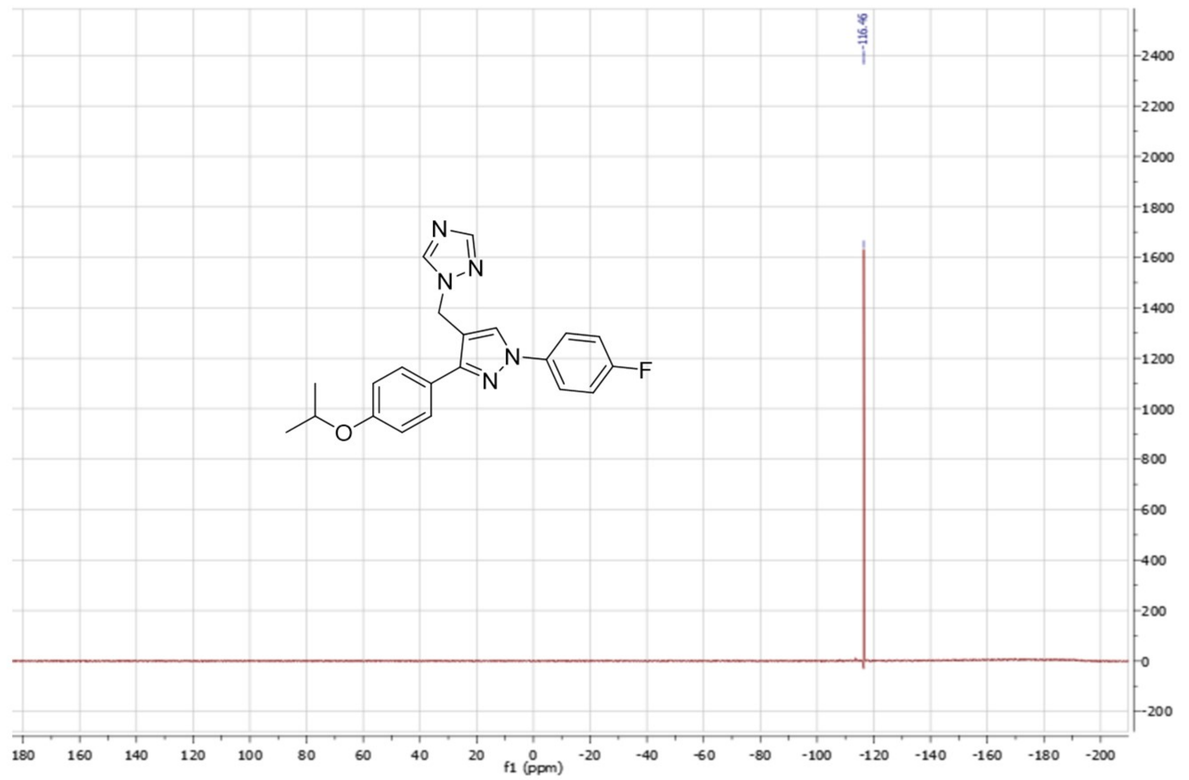

^{1}H NMR spectrum (500 MHz, DMSO-d$_6$) of **12h**:

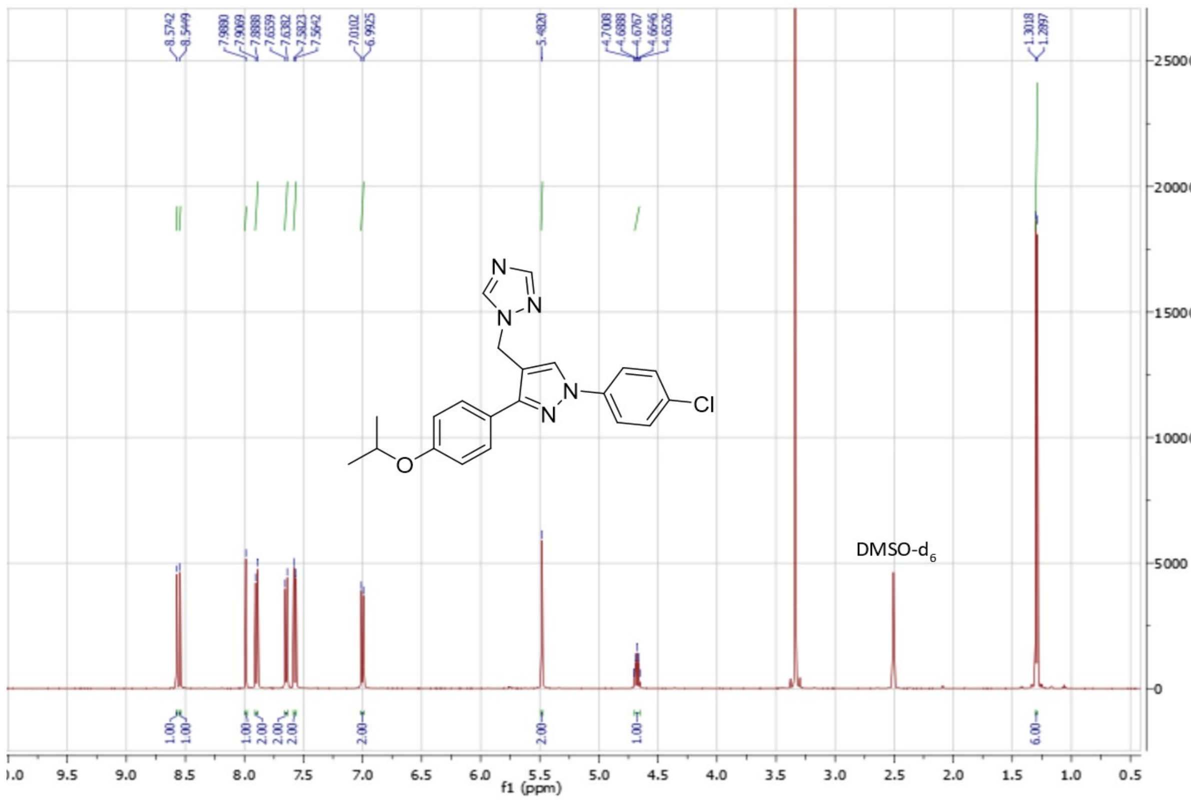

^{13}C NMR spectrum (125.75 MHz, DMSO-d$_6$) of **12h:**

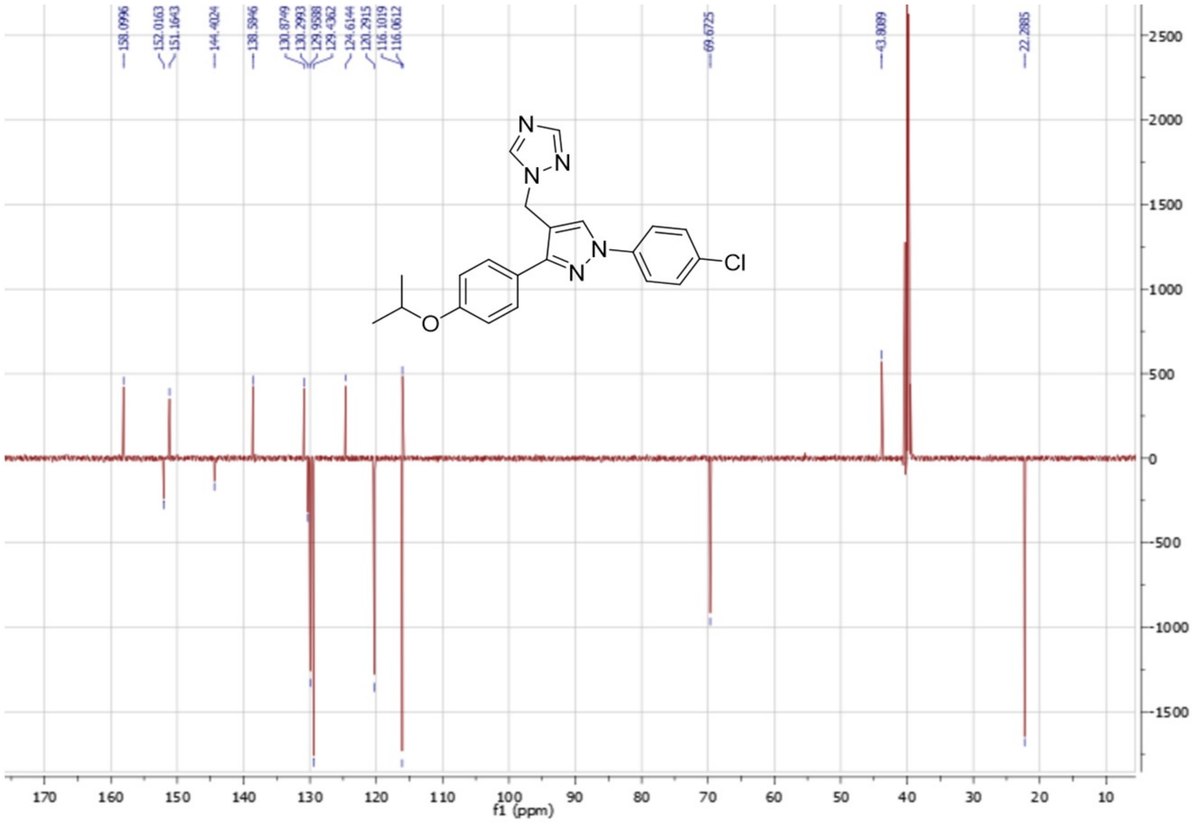

11a	11c	11d	11e

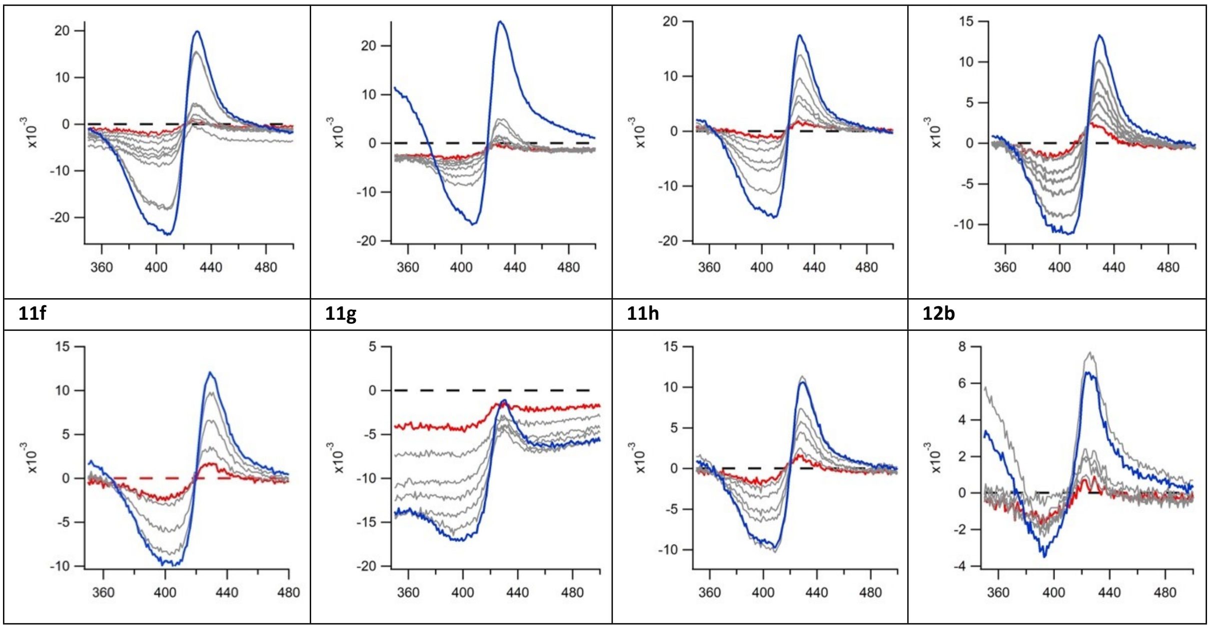

Figure S1. Binding difference spectra of pyrazole derivatives **11a, 11c-11h** and **12b**

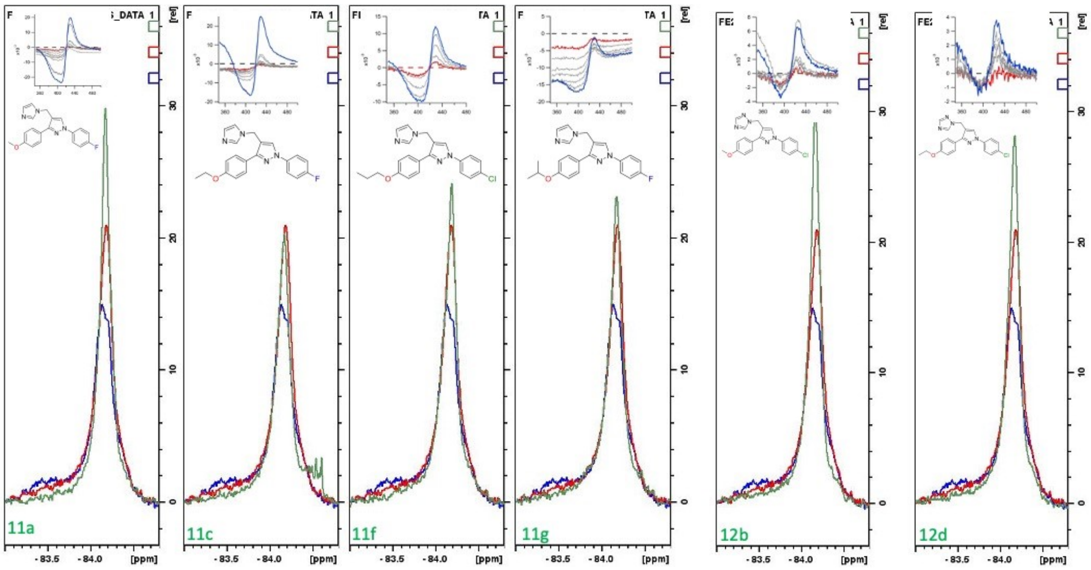

Figure S2. ^{19}F NMR spectra of BTFA-labelled CYP121_S171C (blue) alone, with cYY bound (red) and with exemplar pyrazole ligands (green)

11a

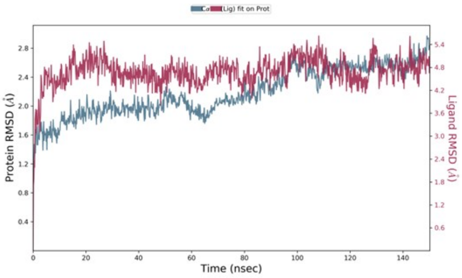

11b

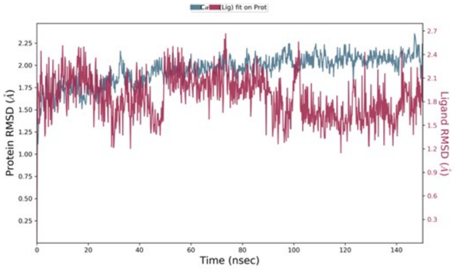

11c

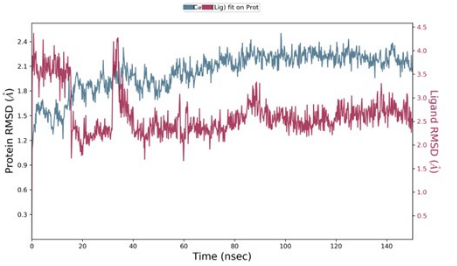

11d

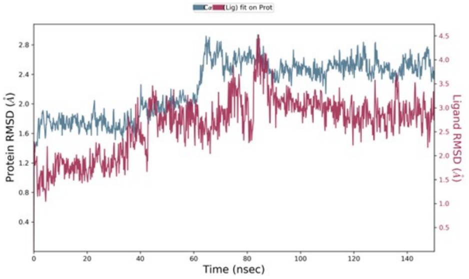

11e

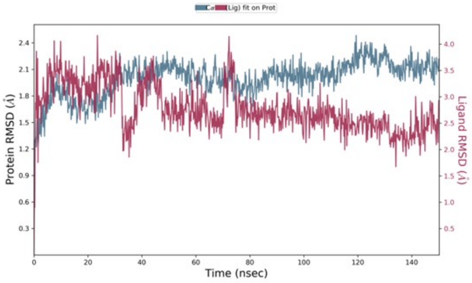

11f

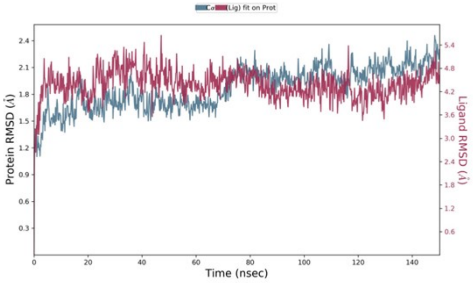

11g

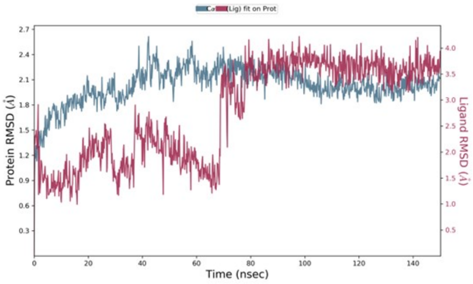

11h

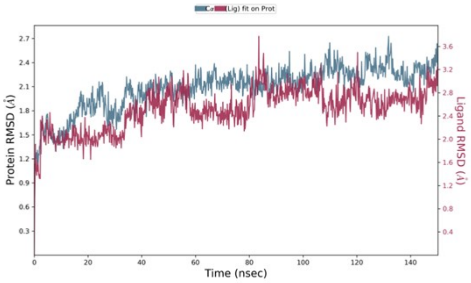

Figure S3. Ligand-protein complex stability through protein-ligand RMSD over 150 ns MD simulation for compounds **11**. Ligand RMSD in red and protein RMSD in blue.

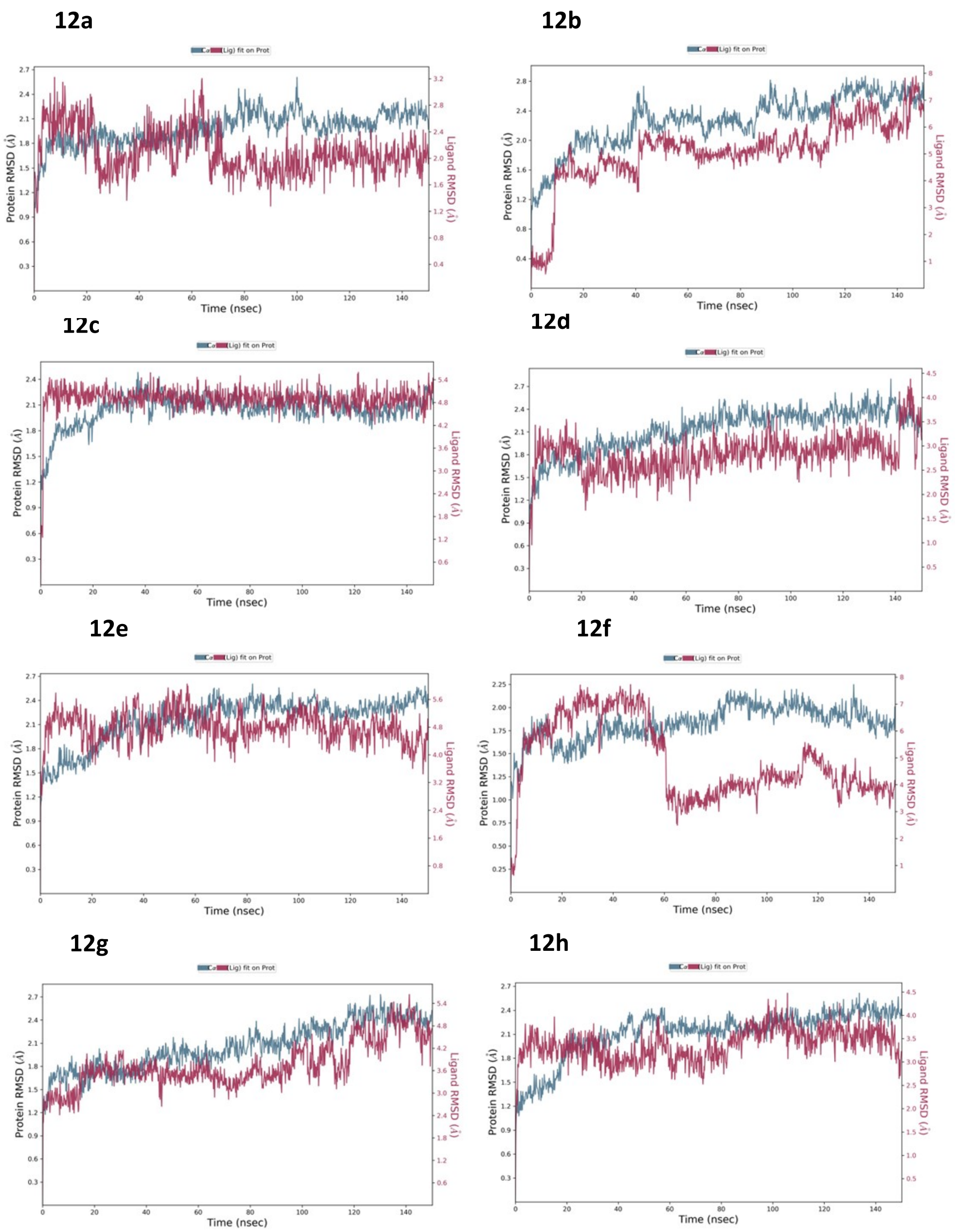

Figure S4. Ligand-protein complex stability through protein-ligand RMSD over 150 ns MD simulation for compounds **12**. Ligand RMSD in red and protein RMSD in blue.
